# Conductive Polyvinyl Alcohol-Based Hydrogels for Wearable Electronics: Preparation Strategies, Conductivity Enhancement, and Sensing Applications

**DOI:** 10.3390/gels12100889

**Published:** 2026-10-01

**Authors:** Xiaoxu Liang

**Affiliations:** School of Arts and Sciences, Guangzhou Maritime University, Guangzhou 510725, China; liangxiaoxu@gzmtu.edu.cn

**Keywords:** conductive PVA-based hydrogels, wearable electronics, conductivity enhancement, multifunctional materials, flexible sensors, physiological monitoring

## Abstract

Conductive poly(vinyl alcohol) (PVA)-based hydrogels combine electrical functionality with the mechanical compliance and biocompatibility required for wearable bioelectronics. However, simultaneously achieving mechanical robustness, stable electrical conductivity, and long-term hydration stability remains challenging. Adopting a performance trade-off perspective focused on wearable sensing, this review examines recent advances in five interconnected areas: (1) fabrication strategies, including freeze–thaw cycling, the Hofmeister effect, mechanical training, and green synthesis routes; (2) conductivity enhancement through electron-conducting pathways (carbon nanofillers, MXene, and conducting polymers), ionic routes (salt and electrolyte doping), and hybrid systems; (3) wearable sensing applications, including physiological monitoring (ECG/EMG/EEG), multimodal biosensing of biofluid analytes, and intelligent sensing devices; (4) environmental resilience and mechanical durability; and (5) regulatory, manufacturing, and clinical translation challenges. Representative studies report tensile strengths exceeding 19 MPa, electrical conductivities above 10 S/m, gauge factors above 10, and self-healing within 1 s; however, these values were obtained for different formulations and under different testing conditions. The review further identifies critical gaps in reproducibility, comparable testing protocols, device-level integration, and scalability, and outlines design considerations for advancing conductive PVA-based hydrogels toward reliable wearable health monitoring systems.

## 1. Introduction

The emergence of wearable electronic devices represents a paradigm shift in biomedical sensing platforms, enabling continuous, non-invasive real-time monitoring of human motion, physiological signals (ECG, EMG, EEG), and biochemical markers without invasive procedures [1,2,3,4,5,6]. However, conventional rigid electronics fundamentally fail to achieve the mechanical compliance, skin-like softness, and intimate tissue conformability essential for truly comfortable and long-term wearable and implantable applications—a critical limitation that has historically hindered the development of genuine wearable bioelectronics [7,8,9,10,11]. This fundamental mismatch between device rigidity and biological flexibility demands materials that simultaneously combine biocompatibility, mechanical flexibility, and electrical conductivity, a combination that traditional electronics cannot provide [12,13,14,15,16].

Polyvinyl alcohol (PVA)-based hydrogels have emerged as promising materials for addressing this challenge [17,18]. Unlike most alternatives, PVA offers a unique constellation of intrinsic advantages: inherent water solubility, abundant hydroxyl groups enabling extensive hydrogen-bonded networks, well-established biocompatibility and biodegradability, and readily tunable mechanical properties through controlled cross-linking [19,20,21,22]. Over the past decade, creative material engineering has systematically transformed PVA hydrogels from purely mechanical platforms into multifunctional conductive systems by incorporating diverse conductive fillers, conducting polymers, and ionic dopants while preserving structural integrity and hydration stability. The resulting class of materials now functions simultaneously as strain sensors, pressure sensors, bioelectrodes for the acquisition of electrophysiological signals (ECG, EMG, and EEG), and electrochemical biosensing platforms—all while maintaining the comfort, compliance, and biocompatibility of soft polymers [23,24,25,26,27].

Despite these advances, simultaneously achieving mechanical robustness, stable electrical conductivity, and long-term hydration stability remains challenging [19,28,29]. From a preparation perspective, freeze-thaw cycling has dominated the field, wherein repeated thermal cycles promote crystalline domain formation through intermolecular hydrogen bonding, now routinely achieving tensile strengths exceeding 19 MPa and fracture energies around 187.5 kJ/m^2^—establishing a robust mechanical foundation [30,31,32,33,34,35,36,37,38,39].

From a conductivity perspective, three complementary mechanisms enable electrical transport: (1) electron-conducting pathways using carbon-based nanofillers (carbon nanotubes, graphene, MXene) for high sensitivity and rapid response; (2) ion-conducting routes utilizing salt, zwitterionic, or ionic liquid doping for low-impedance bioelectrode interfaces; and (3) hybrid systems combining both mechanisms for enhanced versatility. However, each approach presents distinct trade-offs between conductivity magnitude, mechanical compliance, dehydration resistance, and sensing linearity—a fundamental tension that continues to constrain device performance [1,2,3,40,41,42,43,44,45,46,47,48,49,50,51,52].

Critical obstacles that continue to restrict deployment of high-performance PVA-based hydrogel devices include: (1) filler aggregation phenomena in aqueous environments driven by van der Waals interactions; (2) weak interfacial adhesion at inorganic filler-organic matrix boundaries compromising long-term stability; (3) inevitable conductivity degradation upon dehydration, a consequence of the hygroscopic nature of PVA hydrogels; and (4) reproducibility limitations arising from synthesis sensitivity and performance variability across different preparation batches. Furthermore, the path from laboratory innovation to clinical deployment remains substantial: challenges in device integration architecture, manufacturing scalability, and power management continue to impede widespread adoption [2,19,53,54,55,56,57].

Previous reviews have summarized composite strategies and preparation routes for conductive PVA-based hydrogels, including their applications in flexible strain sensors, supercapacitors, and triboelectric nanogenerator sensors. Broader reviews have also examined conductive PVA-based hydrogels for wearable health monitoring and therapeutic applications [1,21,48,58,59,60,61,62]. Building on these contributions, the present review adopts a performance-trade-off perspective focused on wearable sensing. Its contribution lies in bringing together fabrication-induced network structures, electronic/ionic/hybrid conduction pathways, and application-specific sensing requirements, with particular attention to the competing demands of electrical conductivity, mechanical compliance, and hydration stability. This perspective emphasizes how material-design choices affect both sensing functionality and the practical limitations of wearable use, rather than treating improvements in individual properties as sufficient indicators of device suitability.

As outlined in Figure 1, the discussion connects preparation and reinforcement strategies—including freeze–thaw cycling, the Hofmeister effect, mechanical training, and sustainable synthesis—with conductivity enhancement and wearable sensing applications. Human-motion sensing, electrophysiological recording, and biochemical monitoring are considered alongside environmental resilience and mechanical durability. The review further relates these material-level considerations to reproducibility, device integration, scalable manufacturing, and clinical translation. Taken together, this synthesis aims to clarify the design compromises and unresolved challenges that should guide the selection of conductive PVA-based hydrogels for specific wearable applications.

## 2. Preparation of PVA-Based Hydrogels

PVA-based hydrogels are prepared primarily through physical or chemical crosslinking. Because PVA is water-soluble, crosslinking is required to form a stable network that can absorb water or biological fluids while maintaining its structural integrity. The degree of crosslinking influences fluid uptake and the physical, chemical, and diffusion properties of the hydrogel, thereby affecting its suitability for specific applications. This section reviews these preparation methods, each of which may be applied individually or in combination during the fabrication of PVA composite hydrogels. Additionally, Table 1 compares the crosslinking strategies of PVA hydrogels.

### 2.1. Physical Cross-Linking Method

#### 2.1.1. Freeze-Thaw Treatment

Traditional PVA hydrogel preparation via multiple freeze–thaw cycles generates crystalline regions that obtain mechanical properties through a well-defined mechanism [17,30,31]. As temperature decreases, water freezes into ice crystals, progressively concentrating PVA molecules in unfrozen regions. When PVA reaches a supersaturated state, intermolecular hydrogen bonding induces crystalline domain formation. Upon thawing, these crystalline domains anchor polymer chains, maintaining structural stability [64]. Hydrogel properties are significantly influenced by gelation parameters (duration, temperature, and cycle number) and material factors (PVA molecular weight, concentration, and degree of hydrolysis) [83]. Optimal thawing rates below 10 °C/min, preferably 0.2 °C/min, enhance crystallization [84]. Fully hydrolyzed PVA (98.5%) yields stronger hydrogels than partially hydrolyzed variants (88%) [83], while increased PVA concentration improves mechanical properties and stability but modulates pore size and water uptake; typically 10 wt% minimum is employed [85,86]. Improved freeze–thaw methods, including under-drawing and directional freezing, now yield enhanced mechanical properties (tensile strengths 0.3–1.2 MPa, moduli 0.03–0.10 MPa, fracture energies 160–420 J/m^2^) [63,64,65]. To further improve mechanical performance, researchers have adopted biomimetic approaches inspired by natural composites, which achieve exceptional strength and toughness through hierarchical, layered structures [87,88]. Deville’s directional freezing method for ceramic slurries—wherein ceramic particles concentrate between ice crystals subsequently removed through sublimation—demonstrates how controlled ice crystal orientation creates enhanced layered microstructures [89]. Applied to hydrogels, Li et al. developed unidirectional freeze-thawing, producing materials twice as strong and tough as natural conch shells [90]. In this approach, temperature gradients during freezing cause preferential crystallization along the freezing direction, resulting in aligned crystals and elongated pores that confer anisotropic mechanical properties—higher strength along the freezing axis but weaker perpendicular strength [91]. Conversely, Liang et al. constructed bidirectional freeze-thawing inspired by the lobster’s abdomen, applying simultaneous freezing in vertical and horizontal directions [92,93]. This yields uniform crystal and pore alignment in both directions, producing a homogeneous microstructure with isotropic mechanical properties.

While freeze-thaw PVA hydrogels exhibit excellent strength and toughness, their fatigue resistance remains inadequate [94]. To address this limitation, drying-annealing treatment was implemented, wherein hydroxyl groups undergo unsaturation, chain scission, and cross-linking reactions, increasing cross-linking and crystalline domain density [95]. Crystalline regions require greater energy for failure during fatigue crack propagation compared to amorphous chains alone. Liang et al. synergistically combined directional freezing with drying-annealing, using temperature gradients to induce directional ice-column growth and concentrate polymer chains in interstitial spaces [64]. Upon ice removal, sacrificial templates yielded anisotropic hydrogels mimicking human ligament architecture. Standard freeze–thaw cycles involve freezing aqueous PVA solutions at −10 to −40 °C for 12–24 h, followed by thawing at 25 °C for 1–3 h [21,85,86,96]. PVA’s regular linear structure and abundant hydroxyl groups facilitate ordered hydrogen-bonded arrays, creating crystalline cross-linking networks. These physically cross-linked hydrogels are preferred for tissue engineering, drug delivery, and wound dressing applications due to high purity, mild gelation conditions, and the absence of external cross-linkers [97,98,99,100,101,102]. They offer excellent mechanical strength, elasticity, and viscosity; however, they are temperature-sensitive and exhibit poor transparency [103].

Nonetheless, repeated freeze–thaw processing requires extended time and temperature control, while traditional methods produce hydrogels with limited mechanical strength for broader application. Additionally, elevated temperatures disrupt hydrogen bonds and microcrystalline regions, causing hydrogels to revert to a solution state, necessitating further advancement [17,104].

#### 2.1.2. Hofmeister Effect

The Hofmeister effect, discovered in 1888, categorizes ions by their hydration capacity: well-hydrated “kosmotropes” strengthen networks while weakly hydrated “chaotropes” weaken them. Ions modulate PVA–water interactions through three mechanisms: disrupting polymer-hydration hydrogen bonds via water polarization, increasing surface tension around polymeric cavities, and directly binding PVA chains to enhance solubility through charge modification [41,66,105,106,107,108,109,110,111,112,113,114,115]. Salting-out ions (Cit^3−^, SO_4_^2−^) sequester water from the polymer domain, promoting chain aggregation and hydroxyl hydrogen bonding that generate rigid crosslinks while remaining external to the network—enabling complete removal without compromising biocompatibility [32,116,117,118].

Recent advances employing the Hofmeister effect—particularly the salting-out mechanism—have proven effective for constructing strong and tough hydrogels. Yu et al. systematically investigated the mechanical properties of hydrogels with varying salt solution concentrations. The recyclable hydrogels (PCCN hydrogel) exhibit a tensile strength of 0.49 MPa, an elongation at break of 481%, and a maximum toughness of 0.81 MJ/m^3^—substantially surpassing the metal ion-free PCCN-0 hydrogel. This enhancement stems from two complementary mechanisms: formation of abundant metal coordination bonds and, via the Hofmeister effect, increased intermolecular interactions that strengthen surface tension, promote chain aggregation, and establish a multiple network structure. Together, these effects reinforce intermolecular forces and stabilize the crosslinked network architecture, collectively improving mechanical robustness [115]. Cui et al.’s alginate/PVA double-network immersed in ZnSO_4_ reached a tensile strength of 15 MPa with a fracture strain of 750%, demonstrating that careful network manipulation imparts both enhanced mechanical properties and novel functionalities [119]. Zhang et al. employed a directional freezing-stretching (DFS) strategy combining salting-out treatment and mechanical stretching (as shown in Figure 2I): DFS_4_ hydrogel exhibited elongation at break of 163.1%, tensile strength of 27.1 MPa, toughness of 25.3 MJ/m^3^, and elastic modulus of 21.5 MPa—representing 68-, 79-, and 307-fold improvements over untreated controls [66]. Duan et al. achieved heat-resistant crystalline hydrogels (tensile strength 23.82 MPa, fracture energy 47.65 kJ/m^2^, structural integrity to 100 °C) through annealing-enhanced thermodynamic stability and strengthened amorphous-region hydrogen bonding [120]. Sang et al.’s gelatin/glucose hydrogel with sodium citrate soaking demonstrated fracture strength of 2.7 MPa, fracture strain of 932%, toughness of 9.5 MJ/m^3^, and ionic conductivity of 0.97 S/m, sufficient to power LED devices and sustain 1.5 kg mechanical loads (as shown in Figure 2II) [33].

Unlike conventional salt solutions, ionic liquids enable superior ion-exchange capacity and thermal stability. Pan et al. combined localized alignment-assisted salting-out with fixed-end mechanical tension, stabilizing micro-oriented structures while promoting PVA chain aggregation and crystallization. Resulting ionic gels exhibited tensile strength of 18.1–62.2 MPa, toughness of 56.8–123.7 MJ/m^3^, and modulus of 18.8–187.8 MPa, with impact resistance exceeding Kevlar performance [121].

However, ion diffusion rate control and regional concentration uniformity during salting-out remain challenging for large-scale production, restricting consistent property achievement in bulk hydrogels [28,106].

#### 2.1.3. Mechanical Training

Inspired by the ordered fibrous architecture and superior mechanical properties of biological tendons and ligaments, researchers have developed anisotropic PVA-based hydrogels that mimic tendon fiber alignment at the microscopic level [68,122,123], thereby significantly enhancing strength and fatigue resistance [68,124,125]. This biomimetic approach—leveraging the structural organization that enables weight-bearing and movement in native tissues—has emerged as a key design strategy for mechanically superior hydrogels [67,68,69].

Progressive strengthening strategies have emerged as a key research direction. Lin et al. pioneered mechanical training by subjecting PVA hydrogels to repeated stretching cycles to mimic nanofiber organization in skeletal muscle [122], achieving tensile strength of 5.2 MPa while maintaining high water content (84 wt%) and biocompatibility. Subsequently, Liu et al. enhanced this approach by inducing PVA crystallization during stretching and incorporating [Fe(CN)_6_]^4−/3−^ redox pairs through ion infusion, achieving fracture strength up to 19 MPa and demonstrating that ionic additives effectively synergize with mechanical cycling [126].

Recently, Zhang et al. employed a synergistic fabrication strategy combining freeze–thawing, mechanical training, and solvent exchange to construct hierarchically structured polyvinyl alcohol-based hydrogels (as shown in Figure 3I) [124]. Following repeated freeze–thaw cycles, the hydrogels were immersed in a glycerol/water solvent containing ferric chloride and subjected to approximately 200,000 mechanical training cycles, yielding a material with exceptional comprehensive properties: tensile strength of 9.38 MPa, fracture energy of 187.5 kJ/m^2^, fatigue threshold of 7850 J/m^2^, conductivity of 0.64 S/m, and remarkable flexibility even at −80 °C [124]. Lou et al. developed a multi-step fabrication strategy combining freeze–thawing, directional stretch–drying, and thermal annealing in glycerol to produce ultrastrong and tough anisotropic PVA organogels (as shown in Figure 3II) [36]. Freeze–thawing cycles facilitate PVA chain entanglement and hydrogen bond network formation, while directional stretch–drying induces molecular chain orientation and enhances intermolecular interactions. Subsequent thermal annealing promotes chain rearrangement and densifies the hydrogen bond network, collectively yielding remarkable properties: toughness of 416 MJ/m^3^, tensile strength of 61.14 MPa, fracture strain of 961%, optical transparency of ~90%, and excellent stability at sub-zero temperatures. These combined properties position the gels as promising candidates for bionic tendons in soft robotics and transparent protective applications. But the requirement for precise optimization of multiple parameters (PVA concentration, pre-stretch ratio, annealing temperature, and time) and potential brittleness if network densification becomes excessive, which can impede ductility [36].

In summary, through combined application of mechanical training, ion infusion, and controlled drying processes, the tensile strength of hydrogels can be progressively enhanced to produce anisotropic materials with high tensile strength and outstanding mechanical performance.

#### 2.1.4. Solvent-Exchanged and Solvent-Regulation

Generally, physical-crosslinked hydrogel fabrication faces a fundamental constraint: insufficient non-covalent interactions promote polymer dissolution, while excessive interactions impede network homogeneity [127,128,129]. Solvent exchange circumvents this trade-off by modulating polymer interactions through sequential solvent replacement [128,130,131,132].

The fundamental principle of solvent exchange was demonstrated by Xu et al. using PVA as a model system [128]. Dissolving PVA in a strong hydrogen bond acceptor (dimethyl sulfoxide) promotes stretched polymer conformations and complete dispersion by favoring polymer–solvent over polymer–polymer interactions. Transferring to water—a weaker acceptor—restores intra- and intermolecular hydrogen bonding, yielding hydrogels with exceptional stiffness, toughness, antiswelling properties, and underwater adhesion. As shown in Figure 4I, this principle is implemented through a multi-step process: solvent exchange from the good solvent to a poorer solvent recovers interpolymer hydrogen bonding and establishes a homogeneous network with low-density crystalline domains; subsequent salting out promotes further crystallization, generating additional crystalline domains uniformly distributed throughout the network. During deformation, the well-dispersed crystalline domains initially remain intact to restrict network deformation, then adaptively disentangle to dissipate energy, thereby reconciling stiffness and toughness by exploiting the complementary deformation-resisting and energy-dissipating capacities of robust yet dynamic crystalline domains [39]. The resulting sal-exogels achieve exceptional mechanical performance with a coordinated combination of properties: stiffness of 52.3 ± 2.7 MPa, toughness (fracture energy) of 120.7 ± 11.7 kJ/m^2^, and tensile toughness of 199.0 ± 8.0 MJ/m^−3^, simultaneously exceeding the Lake–Thomas model limit that constrains conventional hydrogels [39]. Yao et al. enhanced this approach by combining solvent exchange with salting-out under stretching, inducing PVA chain and crystallite orientation while promoting aggregation and hydrogen bonding. This multiscale synergy achieved tensile strengths of 26.10 MPa and elastic moduli of 4.89 MPa under optimized (NH_4_)_2_SO_4_ conditions, soaking time, and drawing ratio [133].

Building on single-solvent exchange methods, researchers have developed two-step replacement strategies and multi-solvent systems to further enhance mechanical properties [124]. Wu et al. sequentially replaced DMSO with glycerol before final water immersion, incorporating thermal annealing to reconstruct the polymer network [134]. Gu et al. extended this approach via multi-solvent high-temperature annealing of polyvinyl alcohol–alkali lignin hydrogels (DR-PVA-AL). Ethanol immersion and elevated-temperature treatment induced gradient crystalline structures—soft phases surrounding hard crystalline domains—enabling stress transfer and energy dissipation. The resulting hydrogels exhibited a modulus of 74.4 MPa, a toughness of 90 MJ/m^3^, a tear resistance of 34,000 J/m^2^, a tensile strength of 24.8 MPa, and a compressive strength of 60 MPa (as shown in Figure 4II) [135].

**Figure 4:**
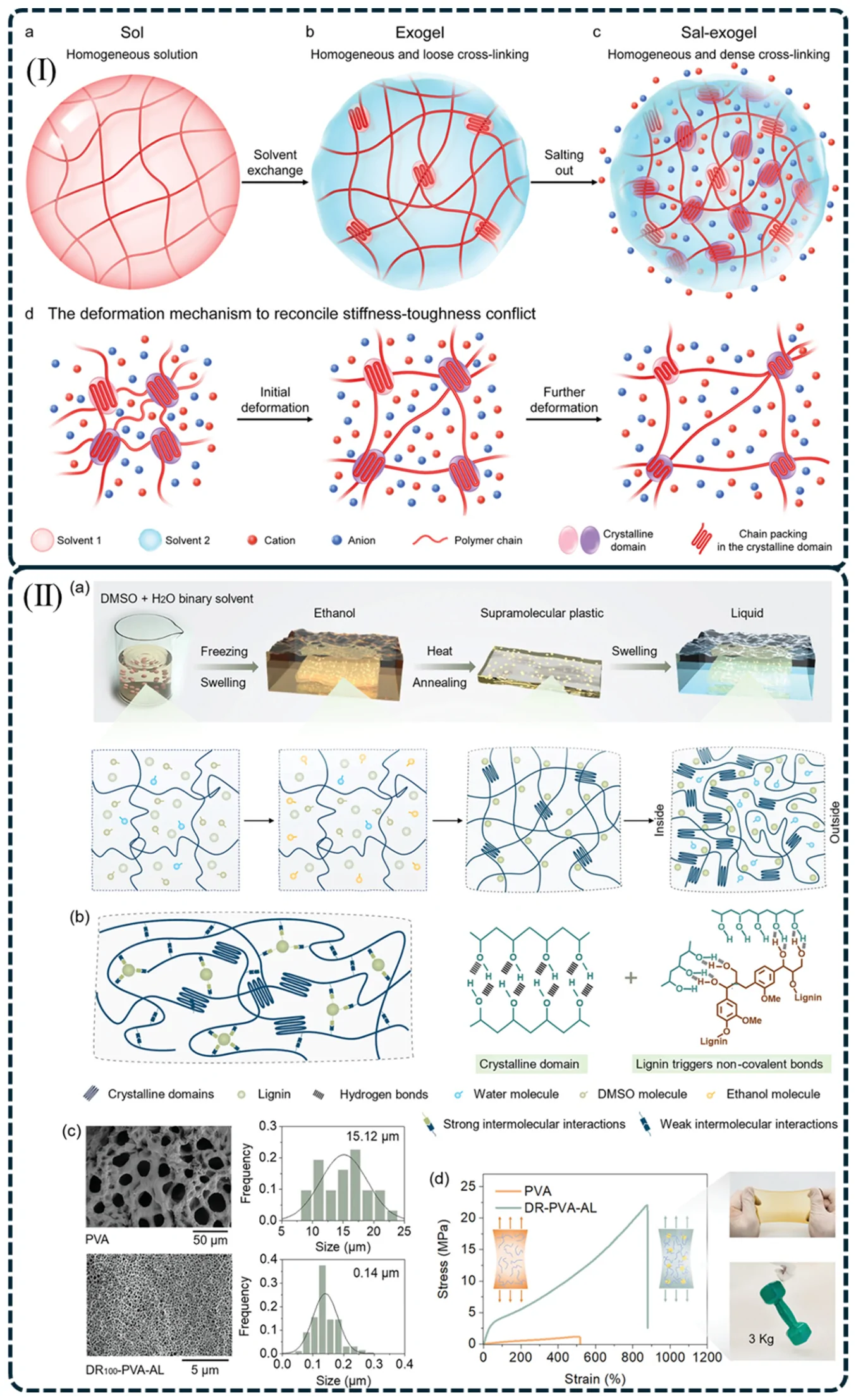
(**I**)—The design of a homogenized PVA network cross-linked by high-number-density crystalline domains for the stiff and tough sal-exogel and its deformation mechanism to reconcile the stiffness–toughness conflict. Reprinted with permission from [39]. (**II**)—Construction of DR-PVA-AL hydrogels. (**II**-**a**) Schematic representation of the preparation of DR-PVA-AL hydrogels. (**II**-**b**) Mechanism of synergistic enhancement between non-covalent bonding and gradient crystal structure in DR-PVA-AL hydrogels. (**II**-**c**) Scanning electron microscope (SEM) images and corresponding pore size distributions of PVA hydrogel and DR100-PVA-AL hydrogel (which “100” denotes the temperature during high-temperature annealing). Random selection of 100 pore data. (**II**-**d**) Tensile stress-strain curves of PVA hydrogel and DR100-PVA-AL hydrogel, and optical images of DR100-PVA-AL hydrogel. Reprinted with permission from [135].

Solvent replacement processes remain time-consuming, limiting large-scale production and industrial deployment. Optimization of multiple parameters—polymer type, salt concentration, salting-out time, and ionic composition—is critical, as mechanical properties exhibit sensitivity to parameter variation. This limitation is substantially offset by demonstrated versatility across diverse polymer systems, positioning solvent exchange as a broadly applicable strategy despite process complexity.

The preparation should be evaluated not only by the initial properties of the hydrogel but also by their retention during storage and use. In particular, assessments should distinguish evaporation-driven dehydration from syneresis and examine time-dependent changes in dimensions, water content, mechanical properties, and electrical response. Reporting the interval between preparation and testing, together with storage conditions, would improve comparisons between formulations. These considerations complement the durability discussion in Section 5.

### 2.2. Covalent Cross-Linking Methods

Covalent cross-linking of PVA proceeds via reactions between its hydroxyl groups and functional groups on exogenous molecules, employing either radiation or chemical reagents as primary methodologies.

#### 2.2.1. Chemical Reagent Cross-Linking Methods

Chemical cross-linking transforms polyvinyl alcohol (PVA) into a highly stable and functional material by forming covalent bonds between polymer chains, thereby enhancing mechanical strength, chemical stability, and resistance to dissolution [136,137,138]. This process reduces PVA’s hydrophilicity by decreasing free hydroxyl groups available for water binding, yet the resulting hydrogels retain significant water absorption capacity through swelling [62,139,140,141]. These materials have found applications in membrane technologies (pervaporation, reverse osmosis, and desalination), food packaging, wound dressings, drug delivery systems, dye retention, and fuel cells [71,140,142,143,144,145]. Chemical cross-linkers—including aldehydes (formaldehyde, glyoxal, and glutaraldehyde), dicarboxylic acids, epichlorohydrin, borate compounds, and urea oligomers—react with PVA’s hydroxyl groups to create acetal, hemiacetal, or ester bridges [38,62,70,71,72,73]. The extent of cross-linking can be precisely controlled by adjusting the cross-linker concentration and reaction parameters, enabling fine-tuning of mechanical properties, water resistance, and permeability [17,70,146]. Notably, glutaraldehyde cross-linkers form flexible bridges that enhance swelling capacity and pH sensitivity, resulting in higher drug loading capacity (10%) and faster release in acidic conditions, whereas radical-based agents (e.g., ammonium persulfate) create tight cross-links that limit swelling but offer stable networks [147].

Advanced cross-linking methodologies have expanded beyond traditional esterification and etherification to include click chemistry, bioconjugation, and dynamic bond formation [73,148,149,150,151,152]. For instance, gradient membranes, featuring cross-linking densities that decrease from surface to interior, can be fabricated through controlled surface cross-linking with glutaraldehyde, providing tunable permeability and selectivity for applications such as cell encapsulation and bioseparation [153]. Functionalization of PVA’s hydroxyl groups through esterification, acetalization, amination, sulfonation, and azidation further enhances polymer versatility [154,155,156,157,158]. Notably, incorporating monocarboxylic or polycarboxylic acids (e.g., citric acid, tartaric acid, and sebacic acid) into PVA solutions facilitates hydrogen bonding and creates robust networks with superior mechanical properties and toughness [115,159,160,161].

Borate complexes represent another versatile approach, forming reversible sol-gel transitions through ionic interactions when PVA approaches its overlap concentration [73,81]. Natural compounds, such as quercetin (at a 50:1 *w*/*w* ratio), have emerged as promising cross-linkers, conferring enhanced mechanical strength, water resistance, and antioxidant properties suitable for biomedical applications [18]. For instance, a sprayable bio-inspired hydrogel (SOPB) incorporated natural okra mucilage as a primary component, combined with PVA and borax cross-linking, with ionic conductivity enhancement through strategic NaCl incorporation between dual-layer structures to facilitate ion transport through the porous network (as shown in Figure 5). The optimized hydrogel exhibits exceptional performance in surface electromyography (sEMG) and ECG measurements, performing comparably to commercial Kendal H135SG electrodes, while effectively monitoring diverse human motions, including finger bending, wrist flexion, knee movement, tapping, and walking, with demonstrated capability to detect emotional states through facial expressions [7].

Despite the extensive potential of chemical cross-linking, significant challenges persist, primarily involving the biotoxicity and difficulty in removing residual cross-linkers. These contaminants can harm tissue cells and trigger inflammatory responses post-implantation, thereby compromising biocompatibility. Consequently, the development of effective yet biocompatible cross-linking strategies remains a critical research priority, driving innovation toward environmentally and biologically benign alternatives that maintain the advantageous properties of chemically cross-linked PVA hydrogels for applications ranging from cartilage repair to radiation shielding.

#### 2.2.2. Irradiation Cross-Linking Methods

Radiation-induced cross-linking represents a safe, non-toxic approach to polymer network formation through high-energy irradiation, including gamma radiation, electron beam irradiation (EBI), and X-rays [74,75,76]. These techniques generate free radicals on PVA chains, inducing the formation of covalent bonds that create structurally stable three-dimensional networks without chemical reagent residues.

Radiation dose critically influences the structural and functional properties of PVA hydrogels [162,163]. Lower doses result in sparse cross-linking and larger void spaces, enabling greater solvent absorption and swelling capacity, whereas higher doses produce densely cross-linked networks with enhanced mechanical strength but reduced water retention. This dose-dependent trade-off is exemplified by Yan Shi et al., who fabricated PVA-poly(vinyl pyrrolidone) hydrogels for artificial cartilage using gamma radiation doses ranging from 50 to 150 kGy. Mechanical strength increased progressively with absorbed dose up to 100 kGy but declined at 150 kGy, demonstrating the existence of an optimal irradiation threshold for balancing strength and functionality [76].

EBI enables rapid in situ cross-linking (approximately 30 s) with precise control over network structure and cross-linking degree, offering distinct advantages for biomedical applications. As a clean and non-toxic technology, EBI eliminates the challenges associated with trace amounts of chemical initiators and cross-linkers, which can pose biocompatibility risks and are difficult to remove completely from the final hydrogel product [164,165]. The commonly reported optimal range for PVA-based hydrogels is approximately 25–30 kGy, where gel fraction and mechanical integrity reach maximum values before degradative chain scission becomes significant [77]. For instance, ionogels prepared via in situ EBI cross-linking of PVA/PVP exhibited exceptional mechanical properties, including high stretchability (>1000%), extraordinary toughness (100 MJ/m^3^), ultra-fast response time (60 ms), and a wide pressure detection range (1 kPa^–1^ MPa) [78].

Deep eutectic solvents (DES)—consisting of hydrogen bond acceptors and donors—offer a sustainable approach to overcome conventional flexible electronic skin limitations: uneven conductive filler distribution, poor biocompatibility, and insufficient mechanical strength [52,166,167,168]. Wang et al. synthesized PVA/DES/NaCl elastomers via photo-crosslinking (choline chloride/acrylic acid), forming an interpenetrating network of physically crosslinked PVA and covalently crosslinked poly(acrylic acid) interconnected through hydrogen bonding (as shown in Figure 6). At optimal NaCl content (10 wt.%), the composite achieved 1200% tensile strain, 0.9 MPa tensile stress, 0.44 S/m electrical conductivity, and 0.21 kPa^−1^ pressure sensitivity (0–200 kPa range). DES properties—low toxicity, thermal stability, and environmental sustainability—enable uniform ionic conductivity and >90% optical transparency for direct skin application [52].

Despite its advantages in producing impurity-free products, radiation cross-linking presents significant limitations. The primary concern is potential damage to biological activity during irradiation, necessitating delayed loading of bioactive materials post-crosslinking to prevent radical-induced degradation. Additionally, carbon–carbon (C-C) bond formation between polymer chains renders these hydrogels non-biodegradable [78,164,169,170,171], limiting their applicability in biomedical contexts requiring eventual material resorption.

## 3. Conduction Mechanisms and Enhancements of PVA-Based Hydrogel

PVA hydrogels are promising for wearable electronics owing to flexibility, water absorption, and biocompatibility, but inherent low electrical conductivity limits applicability. Enhancement strategies incorporate conductive fillers, conducting polymers, and ionic species, each establishing distinct charge-transport mechanisms with critical implications for sensing mode, impedance stability, and signal integrity [33,51,172,173]. Table 2 compares the main conductivity-enhancement strategies in terms of mechanism, advantages, and limitations, whereas Table 3 compiles the conductivity and selected mechanical and sensing parameters of representative PVA-based systems discussed in Section 3.1, Section 3.2, Section 3.3 and Section 3.4, with one formulation per row. The tabulated values are taken from the original reports and should be interpreted in the context of the specific formulations, sample states, and testing conditions, rather than as a direct performance ranking.

### 3.1. Conductive Filler Incorporation

Carbon nanofillers, such as carbon nanotubes (CNTs), graphene, reduced graphene oxide (rGO), and carbon black, enhance electronic conductivity primarily by forming interconnected, percolating networks within the PVA matrix. Charge transport occurs through the conductive fillers and across interparticle junctions via direct contact or tunneling across sufficiently small gaps. Consequently, conductivity depends not only on the intrinsic conductivity of the fillers but also on their loading, dispersion, aspect ratio, and junction resistance. Graphene oxide (GO) should be distinguished from graphene and rGO, because its contribution depends on its oxidation state and the presence of other conductive components [174,176,207,209,210,211,212,213,214]. For example, Li et al. developed an anisotropic composite hydrogel by combining PVA with carboxylated CNT using ice-templating and salting-out strategies. The resulting PVA/CNT-S hydrogel exhibits an oriented anisotropic structure with exceptional properties: high water content (65%), outstanding tensile strength (3 MPa), remarkable toughness (4 MJ/m^3^), and electrical conductivity of 0.11 S/m along the alignment axis, while maintaining minimal swelling characteristics [175]. Similarly, He et al. fabricated multifunctional PVA/PAA/AG hydrogels via a one-pot strategy, incorporating carboxyl-functionalized graphene oxide (GO-COOH), which achieved 122.3 kPa fracture stress and 10.56 ± 0.63 S/m conductivity [174].

Beyond carbon materials, silver nanostructures—nanowires (AgNWs) and nanoparticles (Ag-NPs)—provide exceptional intrinsic conductivity for PVA composite applications [180,181]. Zhu et al. synthesized surface-hydroxylated silver nanowires (M-AgNWs; 150.23 μm length, 129.82 nm diameter, ~1157 aspect ratio) via bis(2-hydroxyethyl) disulfide modification. Incorporation into PVA/borax hydrogels yielded dose-dependent performance: 0.1% M-AgNW composite retained 78% tensile strength post-healing; 0.2% composite achieved 3.59 S/m conductivity (pre-healing) and 3.09 S/m (post-healing), corresponding to 86% self-healing efficiency [180].

Silver nanoparticle-embedded PVA/sodium nitrate (PVA:NaNO_3_:Ag-NPs) hydrogels demonstrated 120 V output voltage and 1.5 μA current. Ag-NPs enhanced electrical conductivity to 5.9 S/m and tensile strength to 0.35 MPa, enabling integration into triboelectric nanogenerators (H-TENG) for powering miniature electronic devices [181].

Recent innovations include LM encapsulation systems achieving exceptional conductivity (4.87 S/m) with excellent sensitivity (GF = 2.40) and outstanding fatigue resistance (over 500 cycles) suitable for maternal and infant health monitoring [182].

Overall, conductive fillers enable tailored electrical conductivity, high sensitivity, and multifunctionality [180,181]. Carbon materials offer processing flexibility; metallic fillers provide mechanical stability; 2D materials enable self-healing and multisensory capabilities [176,177,178]. However, agglomeration and inhomogeneous dispersion compromise uniformity [180,215]. Emerging materials like MXenes are prone to oxidation and interfacial instability, causing performance degradation, while high manufacturing costs and scalability issues limit commercial viability [177,178,182,216]. Future work must address filler dispersion uniformity, filler-matrix interfacial strength, material stability, and cost-efficient production processes [180,182,217,218].

### 3.2. Conducting Polymer Doping

Conducting polymers—poly(pyrrole) (Ppy), poly(aniline) (PANI), PEDOT, and poly(p-phenylene vinylene)—possess tunable electrical conductivity, excellent optical and mechanical properties, ease of synthesis, and environmental stability [4,54,183,184,185,186,187,188,219,220]. These polymers enable flexible, lightweight, and biocompatible electronics. Mechanistically, electronic conduction in these polymers arises from doping-generated charge carriers, commonly described as polarons and bipolarons, which are transported along conjugated backbones and between neighboring chains or conductive domains. Thus, conductivity is governed by both the doping state and the connectivity of the conducting phase. In PEDOT:PSS-containing PVA systems, chain rearrangement and controlled phase separation can improve the connectivity of PEDOT-rich domains. Hydrogen bonding with PVA mainly stabilizes the composite structure rather than serving as the primary electronic conduction pathway.

For instance, Li et al. developed a cheese-structured polypyrrole/biochar/PVA (SS-PVA) non-contact sensor wherein the intermediate layer simultaneously functions as friction material and electrode, eliminating interface charge loss inherent to contact devices and achieving ~250% signal enhancement [183]. Hu et al. synthesized a PVA/polyaniline-encapsulated bacterial cellulose (PANI@BC) supramolecular hydrogel via directional freeze–thaw (DFT) processing, which aligns polymer crystals to generate an oriented porous microstructure promoting anisotropic conductive network formation. The resulting PVA/PANI@BC (PTPB) hydrogel exhibits fracture stress exceeding 2 MPa, toughness of 3.75 MJ/m^3^, reliable strain sensing up to 200%, and conductivity of 1.3 S/cm, demonstrating suitability for flexible wearable sensing applications. [185]. A highly conductive, stretchable, and healable electrode was fabricated via blending poly(ethylene dioxythiophene):poly(styrene sulfonate) (PEDOT:PSS) with polyvinylpyrrolidone (PVP), PVA, and tannic acid (TA) [187]. The film exhibits a two-phase separation structure wherein PEDOT chains undergo conformational transformation and aggregation to establish conductive pathways, while PVP and poly(styrene sulfonate) chains simultaneously percolate into the PVA/TA cross-linked network via hydrogen bonding, forming a semi-interpenetrating architecture. This composite film achieves electrical conductivity of 1300 S/cm and mechanical stretchability of 266% strain, maintaining stable resistance (R/R_0_ = 1.06) after 3000 stretching cycles at 100% strain [187].

PVA-based hydrogels doped with conducting polymers are promising candidates for wearable bioelectronic interfaces, exhibiting high conductivity, environmental stability, and tunable electromechanical properties. However, the incorporation of conductive polymers introduces substantial limitations: reduced transparency and mechanical flexibility, compromised polymer homogeneity, diminished stability under cyclic deformations, and poor interfacial bonding between polymer layers. Addressing these material constraints remains essential for advancing conducting polymer-PVA composites toward practical applications [19,221,222].

### 3.3. Ionic Conductivity Enhancement

Salt dopants enhance ionic transport in PVA matrices via ion mobility within hydrated networks, establishing dual-mode conduction pathways. Common dopants include sodium chloride (NaCl), potassium chloride (KCl), and calcium chloride (CaCl_2_) [17,66,189,190,191,192,194,223,224,225,226,227,228,229,230].

For instance, Liu et al. developed a robust hydrogel fiber incorporating syndiotactic-PVA (s-PVA) as a reinforcing agent that achieves exceptional fracture strength of 20 MPa and toughness of 67.2 MJ/m^3^; NaCl addition as an electrolyte confers ionic conductivity of 0.38 S/m, together enabling a high GF of 1.9 across 100–300% strain with excellent sensing stability over 1200 cycles at 50% strain [189]. Similarly, the PVA-B-PAAS-KCl hydrogel system functions as an electrochemical detection matrix, wherein the PVA-B network integrated with polyacrylate chains establishes a confinement environment that rapidly captures released purines while isolating them from oxygen to prevent oxidative degradation; KCl incorporation further amplifies electrochemical signal intensity, yielding approximately 20.5% enhancement relative to undoped systems [190]. She et al. developed a series of PVA/pullulan/MXene/KCl (PKPM-S) hydrogels that undergo solketal-induced network contraction, achieving 5.706 MPa tensile strength (9.6-fold improvement), GF of 4.6, and a 100 ms response time [191]. CaCl_2_ presents a promising candidate owing to its bivalency and coordination with hydroxyl groups, forming reversible physical bonds that substantially increase stretchability [192].

Salt modulation via ion-specific effects on polymer solvation and network structure achieves orders-of-magnitude property enhancements [114,193,194]. For instance, Zhou et al.’s dual-crosslinked starch/PVA/NaCl composites establish denser networks through salt-enhanced hydrogen bonding and crystallization, increasing decomposition temperature by 17.5 °C, while achieving maximum tensile strength of 2.9 MPa (429.8% elongation), storage modulus of 22.4 kPa, and ionic conductivity of 4.2 S/m at 2% (*w*/*w*) NaCl [193]. LiCl functions as a hygroscopic dopant, maintaining optimal hydration via elevated hydration energy while substantially reducing actuation activation voltage [231]. Maksimkin et al. doped PVA with LiCl and NaCl; salt loading equivalent to PVA mass reduces actuation voltage by up to 20 V. PVA/LiCl actuators achieve 87% maximum extension at 20 kPa load versus 75% for PVA/NaCl, with LiCl outperforming NaCl by 10–15% across cycles due to enhanced Li^+^–water interactions [194]. Moreover, dual-cation doping—H^+^ and alkali metal cations disrupting PVA hydroxyl hydrogen bonding and accelerating ion diffusion—yields PVA/HCl/NaCl hydrogels with Seebeck coefficient of 7.43 mV/K and ionic conductivity of 33 mS/cm, substantially exceeding single-cation PVA/NaCl analogues [195].

Advanced salts—including ionic liquids and organic salts—overcome conventional salt limitations (high melting points, handling difficulties) via lower volatility, higher thermal stability, and anti-freezing capacity [42,232,233]. For instance, Zhang et al. synthesized a PVA–PEDOT:PSS/[BMIM]_5_[ZnBr]_1_ ionogel via solvent exchange and thermal annealing, achieving record n-type ionic thermoelectric performance with a negative Seebeck coefficient of −128 mV/K, power factor of 16.7 µW/cm•K^2^ (ZT = 1.1 at room temperature). Strong Zn^2+^–polymer coordination confines cations to the polymer-dominated phase while selectively enabling Br^−^ anion thermodiffusion, decoupling ionic and thermal transport pathways to generate high thermopower without requiring excessive ion concentration [197]. Figure 7 presents the structural architecture, thermodiffusion mechanism, and thermoelectric performance of a [BMIM]_5_[ZnBr]_1_–PVA–PEDOT ionogel. Panel (a) illustrates the ionogel architecture featuring lamellae-embedded networks, Zn^2+^ coordination domains, and thermodiffusion pathways that direct selective Br^−^ migration under vertical temperature gradients. Panels (b) and (c) benchmark the Seebeck coefficient and figure of merit (ZT) against state-of-the-art n-type inorganic and organic thermoelectrics, demonstrating ultrahigh thermopower and competitive overall performance. However, ILs introduce increased complexity, biocompatibility concerns, and higher processing costs [42,196]; deep eutectic solvents (DESs) offer similar advantages but likewise require careful optimization [59,198].

Balancing high ionic conductivity with mechanical strength and stretchability remains limiting. Conventional systems exhibit hygroscopic behavior, conductivity degradation during dehydration, and poor long-term stability; advanced systems (DES) partially mitigate these issues but require solutions addressing biocompatibility, cost, and environmental impact [198,199,200].

### 3.4. Synergistic Multi-Strategy Approaches

Synergistic multi-strategy approaches substantially enhance ionic conductivity, mechanical strength, and durability through collaborative molecular and structural effects [204,206,207]. For instance, Li et al. combined ionic liquids and graphene oxide in starch/PVA hydrogels via physical crosslinking, achieving 1.98 S/m conductivity, 0.64 MPa strength, below −20 °C freeze resistance, and sensor gauge factors of 6.04 (tension), 3.27 (compression), and 0.71%/°C (temperature); hydrogen bonding between imidazolium cations and PVA hydroxyls plus electrostatic interactions between imidazolium rings and acetate ions stabilized this dual ionic/electronic pathway [203]. Separately, Li et al. fabricated a PVA/gelatin/tannin acid (TA)@CNT nanocomposite organohydrogel fiber via freezing–thawing and Na_3_Cit/water–glycerol soaking, wherein TA preserved CNT electronic structure while enabling interconnected conductive networks; concurrent Na_3_Cit doping synergistically reduced electrical resistance, yielding −20–50 °C thermal adaptability, 30-day environmental stability, and reliable 0–628% strain and temperature detection [207].

Conducting polymer hydrogels merge electronic conductivity with mechanical flexibility but suffer brittleness under deformation; combining 2D materials and ionic liquids in nacre-inspired structures circumvents this constraint [204,205]. Thiruchelvam et al. constructed a fully conductive 3D polypyrrole–Ti_3_C_2_T_X_ hydrogel (PMCH) wherein Ti_3_C_2_T_X_ nanosheets served as conductive cross-linkers, creating a hierarchical mesoporous ion-rich environment; at optimized composition (33.33 wt.% Ti_3_C_2_T_X_), achieved 368.3 F/g specific capacitance across a 1 V potential window for wearable supercapacitor applications [204]. In parallel, You et al. prepared MXene-polypyrrole/PVA hydrogels exhibiting 11.3 S/m conductivity, 1235% stretchability, self-healing capability, and ~100% accuracy in EMG/ECG measurements and gesture recognition [51].

However, increased material complexity complicates phase control and reproducibility while risking phase segregation under mechanical and environmental stress. Multi-step processing with advanced nanomaterials raises cost barriers. Future research must establish fundamental material interactions, standardize evaluation protocols, and improve fabrication reproducibility and structure–property relationships for cost-effective wearable translation. [167,203,215,221,234,235].

### 3.5. Conduction Mechanisms in PVA-Based Hydrogels

Conductive PVA-based hydrogels are functionally classified by transport mechanism into three modes—electron-conducting, ion-conducting, and hybrid-conduction—each exhibiting distinct charge-transport pathways with critical implications for sensing mode, impedance stability, dehydration sensitivity, and signal integrity [221,236].

Electron-conducting systems incorporate carbon nanofillers, MXene nanosheets, or conducting polymers to establish interconnected electronic pathways within the hydrogel. MXenes are two-dimensional transition-metal carbides and/or nitrides rather than carbon nanofillers. For commonly used Ti_3_C_2_T_x_ MXene, conductivity enhancement relies on its high intrinsic electronic conductivity and charge transfer through interconnected nanosheets. Interactions between MXene surface terminations and PVA hydroxyl groups help stabilize the filler–matrix interface and conductive network; these structural interactions should not be equated with the electronic transport mechanism. When mobile ions are also present, ionic transport can contribute to the measured conductivity, and the dominant contribution depends on the formulation and measurement conditions [174,176,177,178,207,209,210,211,213]. They excel in strain and pressure sensing through resistance and capacitance modulation, delivering high gauge factors and rapid response times for wearable monitoring [237]. However, continuous conductive networks inherently produce high hysteresis and poor sensing linearity due to contact resistance-based mechanisms [236,238,239]. Composite filler embedding suffers weak interfacial interactions and mechanical property mismatches that compromise stretchability; cyclic deformation further induces filler debonding and microstructural cracking [60]. Surface-embedded conductive networks deposited directly onto polymer substrates circumvent these drawbacks by decoupling the conductive phase from the bulk matrix, delivering superior electrical performance and exceptional sensitivity [240]. Conversely, reducing water dependence enables environmental stability but introduces brittleness and reduced mechanical compliance, requiring careful formulation optimization [204].

Ion-conducting systems utilize salt, zwitterion, or ionic liquid doping within hydrated PVA networks [42,194,232,233,241,242]. Their inherent softness, stretchability, and mechanical skin compatibility establish low-impedance conformal interfaces ideal for bioelectrodes (ECG, EMG, and EEG recording), minimizing skin-electrode impedance and interfacial polarization [243,244] The entrapped liquid phase (water or ionic liquids) enables efficient ionic transport while maintaining mechanical robustness, emulating biological ionic signaling [243]. Critical limitations arise from water and ion mobility dependence: pronounced impedance drift upon dehydration, strong frequency-dependent response kinetics compromise both strain sensing and electromechanical transduction, and ion leaching from the gel generates uneven ionic distribution and degraded sensing accuracy [129,223,243,245].

Hybrid systems exploit electronic and ionic transport simultaneously, enabling multifunctional sensing and bioelectrode functionality [54,203,246,247]. Embedding electronic conductors within ionically conductive PVA matrices preserves efficient ion transport at skin-hydrogel interfaces while establishing electron pathways in the bulk material, supporting enhanced signal sensitivity and reduced impedance [243,248]. Integration of incompatible transport phases presents fundamental challenges: maintaining uniform filler dispersion without compromising polymer softness is difficult; weak metal–polymer bonding promotes filler particle release and catalytic degradation; carbon–ionic conductor combinations risk charge imbalance-induced polarization that degrades signal integrity. Multi-component systems amplify toxicity concerns (leached metal ions and polymer fragments) and trigger complex immune responses, while high production costs and unpredictable interfacial interactions inhibit scalability and clinical implant viability [17,20,61,248].

## 4. Application of PVA-Based Conductive Hydrogel Sensor

Conductive PVA-based hydrogels have been considered as functional flexible platforms through systematic material engineering that integrate multiple performance characteristics. This engineering approach enables independent tuning of electrical conductivity, mechanical strength, and biocompatibility for targeted applications. Recent advances have expanded functional scope across four primary domains: deformation and strain sensing, electrophysiological signal acquisition (ECG, EMG, EEG), electrochemical energy storage and conversion, and controlled drug-delivery systems [17,174,249]. This section will discuss relevant representative advancements.

Despite these capabilities, fundamental challenges persist in device architecture, manufacturing scalability, and power integration—barriers that remain critical for clinical translation and widespread deployment.

### 4.1. Human Motion Detection Sensors

Conductive PVA-based hydrogels merge electrical conductivity with inherent flexibility and biocompatibility, enabling deployment across supercapacitors, strain sensors, and wearable electroactive devices. This convergence positions them as critical materials for next-generation flexible electronics platforms.

For instance, Meng et al. fabricated a triple cross-linked polyvinyl alcohol-borax-ionic liquid hydrogel (PBIH) via freeze-thaw cycling, achieving a tensile strength of 3.1 MPa and a toughness of 670 kJ/m^3^ after three cycles [249]. Integration with the electrochromic polymer PEDOT:PSS yields a variable-fluorescence sensing skin (VFSS) with a rapid response time of 0.3 s, enabling real-time monitoring of complex human activities in wearable electronic applications [249]. Li et al. synthesized a polyacrylamide/polyvinyl alcohol composite hydrogel incorporating tannic acid-modified lignin (TA@AL) through one-pot polymerization and Fe^3+^ post-impregnation, establishing coupled mechanical-electrical functionality [53]. The synergistic integration yields tensile strength of 115 kPa, fracture strain of ~900%, strong tear resistance, universal adhesion, and ionic conductivity of 0.75 S/m. This architecture enables reliable strain sensing with high sensitivity, rapid response, and durability under cyclic deformation, discriminating between large-scale joint motions and subtle physiological signals for wearable monitoring applications [53].

To develop more compatible, effective, and stable sensing material, Ding et al. prepared PVA/sodium alginate/carbon nanotubes (PVA/SA/CNT) composite hydrogels with synergistic double-network crosslinking and hydrogen bonding between CNT and PVA [44]. Optimization at a 9:1 PVA:SA ratio and 5 mg/mL CNT concentration yields 346% stretchability, excellent elastic recovery, and anti-fatigue stability over 1000 cycles at 30% strain [194]. These composites function as sensitive strain sensors, responding to microscale deformations (facial muscle contractions, throat swallowing) and macroscale joint flexions (finger, wrist, and knee movements) with detection limits below 0.4% strain. As skin-equivalent bioelectrodes for electrocardiography, PVA/SA/CNT hydrogels substantially outperform commercial dry electrodes, elevating the signal-to-noise ratio from 7.5 dB to 18.2 dB [44]. He et al. synthesized PVA/PAA/graphene oxide composite hydrogels via one-pot green chemistry, optimizing mechanical properties, environmental stability, self-healing capability, and strain sensitivity simultaneously [174]. Dual crosslinking—AlCl_3_ (physical) and sodium tetraborate decahydrate (chemical)—establishes structural integrity, while carboxyl graphene oxide incorporation amplifies mechanical strength (fracture stress 122.3 kPa) and electrical conductivity (10.56 ± 0.63 S/m). The resulting strain sensor demonstrates exceptional sensitivity (GF = 11.31 at 400–550% strain), enabling real-time discrimination between large-scale joint flexions and subtle facial expressions [174].

Most hydrogels exhibit poor freeze resistance and limited anti-drying capabilities, restricting their deployment in harsh environmental conditions and liquid-based applications [129,250]. Developing hydrogels with enhanced stability and environmental resilience across diverse operational contexts therefore becomes essential [141,251,252]. Han et al. reported a multifunctional PANI/PVA/CMC-P(AM-co-AA)-Zn^2+^/EG hydrogel that decouples mechanical and electrical properties through polyaniline electronic conductivity, Zn^2+^ coordination reinforcement, and hydrogen bonding between ethylene glycol and water for cryogenic stability [253]. The optimized composition achieves a fracture tensile strength of 838 kPa, a strain of 330%, and an elastic modulus of 302 kPa; withstands freezing at −41.6 °C with 83.7% mass retention after 7 days; and exhibits electrical conductivity of 2.02 S/m with a gauge factor of 2.94 across 0–200% strain and was able to monitor body movements such as elbow, finger, wrist, and leg bending. However, compositional optimization reveals critical trade-offs: increased Zn^2+^ concentration (up to 0.12 M) enhances electrical conductivity, but elevated ethylene glycol content reduces it through ion dilution, while multi-component interactions complicate rational design strategies [253]. Solvent-free supramolecular ion-conductive elastomers (SF-supra-ICE) address dehydration vulnerabilities in conventional ion-conducting hydrogels by encapsulating inositol hexakisphosphate (IP6), a naturally ionizable compound, within PVA chains via high-density hydrogen bonding [201]. This architecture maintains high ionic conductivity (>3.3 × 10^−2^ S/m), skin-like softness, and long-term electrochemical stability without external solvent requirements, sustaining stable performance for over 150 days in open air and substantially outperforming hydrogel- and ionogel-based systems [201].

Flexible conductive hydrogels confront persistent barriers—non-green fabrication processes, conductivity-mechanical compliance trade-offs, and heavy additive reliance. Recent advances address these limitations through green synthesis pathways, rational structural design, and multifunctional integration strategies, advancing sustainable and versatile, flexible electronics platforms [227,254,255,256,257]. For instance, Chang et al. developed an eco-friendly multifunctional DES/PVA/CS/TEOS composite hydrogel via one-pot green gelation integrating biomass-derived wood vinegar and deep eutectic solvents (choline chloride-urea) as co-solvents without toxic catalysts or exogenous conductive fillers [257]. Synergistic solvent modulation enables in situ TEOS hydrolysis and covalent grafting to PVA via Si−O−C linkages, generating an open polymer network with expanded interchain spacing. The optimized DES/PVA/CS/TEOS_2_ hydrogel achieves ionic conductivity of 38.1 mS cm^−1^, tensile strength of 0.22 MPa with 300% elongation at break, and robust low-temperature tolerance to −20 °C. This platform enables sophisticated bioelectronic applications, including Morse code recognition via deep learning, 3 × 3 array pressure sensing, encrypted communication, and robotic hand gesture control with 98–99% classification accuracy (as shown in Figure 8) [257]. A complementary freezing strategy incorporates lignin and ionic liquid into the dual-crosslinked network of PVA and gelatin, establishing a multifunctional green composite hydrogel with enhanced mechanical strength, ionic conductivity, and strain sensitivity through hydrogen bonding and electrostatic interactions [256]. Flexible writing sensors and human motion detection devices fabricated from this hydrogel recognize handwriting patterns and monitor daily movements with high signal stability over 500 cycles, demonstrating robust anti-fatigue performance [256].

Underwater deployment presents critical performance barriers: conductive hydrogels suffer signal distortion and accelerated degradation under aquatic conditions due to swelling-induced matrix structure changes [258,259]. Water impact and ion permeation destabilize device function and compromise signal fidelity [41,260,261]. Early zwitterionic approaches remain insufficient for demanding underwater conditions [262]. Ren et al. developed a super-tough, non-swelling zwitterionic double-network hydrogel—PVA/poly(sulfobetaine methacrylate) with H_2_SO_4_ (PVA/PSBMA-H_2_SO_4_)—addressing these limitations through synergistic Hofmeister effects and ion-polymer interactions [41]. The interpenetrating network of rigid PVA and flexible PSBMA chains achieves a rigidity-flexibility balance: kosmotropic H_2_SO_4_ and PSBMA cooperatively nucleate nanocrystalline domains within PVA chains, functioning as primary physical crosslinking sites for energy dissipation, while H_2_SO_4_ induces a potent salting-out effect that densifies the PVA network. The optimized hydrogel achieves ultra-high toughness of 4.61 MJ/m^3^ (24.5% solid content), complete non-swelling (equilibrium swelling ratio <1%), and sustained stability exceeding one month in both pure water and seawater. An oceanic strain sensor fabricated from this hydrogel demonstrates reliable, stable sensing performance in seawater environments (as shown in Figure 9) [41].

Antimicrobial functionality is essential for supercapacitor and wearable sensor reliability, particularly at biological interfaces [263,264,265,266,267,268,269]. Sun et al. developed amphoteric sulfomethylated lignin/quaternized chitosan/PVA (SML/QCS/PVA) hydrogels through synergistic charged-group architectures, wherein SML and QCS moieties promote KOH dissociation and establish ion transport channels amplifying K^+^ and OH^−^ migration [267]. The resulting hydrogels achieve ionic conductivity of 46.64 mS/cm, tensile strain of 927.32%, and compressive strain of 85% under ambient conditions, maintaining electrochemical stability under bending and heavy load while suppressing bacterial proliferation with inhibitory zone diameters exceeding 37.0 mm against *E. coli* and *S. aureus* [267]. Wang et al. developed parallel antimicrobial architectures integrating polydopamine and silver nanoparticles into cellulose nanofibrils (PDA@CNF-Ag) to reinforce PVA via freeze-thaw processing, establishing multifunctional strain sensors with combined mechanical, electrical, and antimicrobial performance [263]. The optimized PVA-PDA/CNF-Ag-0.5 composition achieves electrical conductivity of 17 mS/m, stretchability of 536%, tensile strength of 60 kPa, high sensitivity (GF = 2.1), stable signal responses over 300 cycles, and reliable discrimination between large-scale and subtle human motions, while exhibiting strong, repeatable skin adhesion and robust antibacterial activity. This platform integrates bioelectronic and antimicrobial functionality for wearable applications [263].

Collectively, flexible conductive PVA-based hydrogels enable reliable human motion detection across physiological scales—microscale facial expressions to macroscale joint flexions—through dual-network architectures combining mechanical robustness with rapid electrical response (GF = 2.1–11.31, thresholds < 0.4% strain). Environmental resilience advances (cryogenic stability, solvent-free architectures, anti-swelling networks) extend functionality across temperature extremes and aquatic environments, while antimicrobial functionalization maintains bioelectronic compatibility. These capabilities establish PVA hydrogels as validated wearable motion sensors for demanding biological and environmental applications (as summarized in Table 4).

### 4.2. Healthcare Detection Sensors

Conductive PVA-based hydrogels integrate flexibility, stretchability, adhesiveness, and biocompatibility through facile synthesis, establishing versatile platforms for health monitoring, motion tracking, and bioelectrical signal acquisition. These materials transduce external stimuli into quantifiable electrical signals via resistance or capacitance variations, enabling discrimination of diverse activities—walking, running, respiration, and sleeping—while capturing subtle bioelectrical signatures (ECG, EMG, EEG) through minimal skin-electrode impedance [44,257,270,271]. This section examines emerging healthcare applications of conductive hydrogels.

#### 4.2.1. Electrocardiography (ECG)

Continuous ECG monitoring remains essential for cardiovascular disease assessment and intervention planning. Hydrogel bioelectrodes establish long-term stable skin adhesion with high-fidelity signal capture critical for early diagnosis and therapeutic guidance [270,272,273,274,275].

For instance, Nan et al. synthesized silver nanowire (AgNW)-enhanced PVA hydrogels, achieving 12.54 ± 0.43 kPa adhesion, reduced contact impedance (190.79 Ω), tensile strength of 147.6 kPa, and 300% strain resilience. AgNW incorporation substantially enhanced conductivity to 1.3 S/m compared to PEDOT:PSS-only systems, sustaining eight-hour continuous cardiac monitoring in a wearable configuration [270]. Li et al. developed injectable, self-healable PEDOT:PSS/PVA/borax hydrogels exploiting reversible borax–hydroxyl and hydrogen bonding for direct syringe injection as gel fibers [274]. This architecture achieved ultra-low modulus (~2.5 MPa), reduced on-skin impedance (~45% of commercial electrodes), and high signal-to-noise ratios (15–22 dB) across ECG, EMG, and EEG recording, while enabling reusability through remolding without degrading electrophysiological fidelity or brain–computer interface performance [274].

Current hydrogel bioelectrodes face interconnected limitations—inadequate breathability, moisture loss, and poor skin conformability—that compromise long-term monitoring safety [276,277]. Zhang et al. addressed these constraints through fabric-based composite electrodes integrating needle-punch membranes with SA/PVA/PAM/SGP conductive hydrogels [55]. This architecture achieved tensile stress of 58.8 MPa, skin adhesion of 10.7 kPa, superior biocompatibility (>90% cell viability at 24 h), exceptional breathability (>95 g/m^2^/day), and sustained water retention (≈35% loss over 10 days). Long-term ECG monitoring demonstrated contact impedance and signal quality substantially exceeding commercial Ag/AgCl electrodes [55]. Zhuang et al. developed a novel ionic hydration-engineered, cryo-compatible all-polymer hydrogel (c-APH) synthesized by incorporating PEDOT:PSS nanofibrils into a polyvinyl alcohol (PVA) matrix with Na_2_SO_4_ to overcome electrical-mechanical trade-offs in subzero-temperature bioelectronics, which is laser-processable for customized geometries [278]. The material achieved exceptional electrical conductivity (35.0 S/cm at −20 °C) while maintaining tissue-like softness and demonstrated excellent biocompatibility with 107.7% ± 1.9% cell viability. The hydrogel electrode delivered high-quality ECG signals with signal-to-noise ratios of 15.8–15.3 dB after storage at −20 °C for 4–48 h, comparable to state-of-the-art commercial hydrogel electrodes. Despite adhesion-related challenges, the EMG signal-to-noise ratio (21.5 dB) exceeded literature values for metal- and carbon-based electrodes (17.17–20.70 dB). Figure 10I shows the ECG and EMG measurements using dm-(PEDOT:PSSnano/PVA)9/1-Na_2_SO_4_ hydrogel electrodes. The hydrogels could be used in biomechanical monitoring and integrated human-machine interfaces for extreme-environment bioelectronic systems [278]. Panel (a) shows the measurement setup for both ECG and EMG. Panel (b) compares ECG detection using the hydrogel electrodes and metallic reference electrodes. Panels (c)–(g) document EMG measurements: (c) optical image and amplitude trace of evoked potential; (d)–(f) forearm EMG signals during grip force tests at 11.2, 28.8, and 38.8 lb, with corresponding amplitude quantification; (g) EMG signals produced by finger flexion/extension and distinct hand actions.

#### 4.2.2. Electromyography (EMG)

Surface electromyography signals function as pivotal biomarkers for motor function assessment and rehabilitation monitoring, propagating through myocyte action potentials to the epidermal interface, where signal fidelity depends critically on interfacial ion-to-electron coupling efficiency [279,281,282,283,284]. PVA-based hydrogel electrodes establish tissue-mimetic elasticity and dual ionic/electronic conduction to meet these requirements [285,286].

For instance, Zhou et al. synthesized PVA/PEDOT:PSS/chitosan composite hydrogels integrating hydrogen bonding and electrostatic interactions to achieve a fracture strength of 25 kPa and an elongation of 325% with strong interfacial adhesion [283]. During multimodal recording, these electrodes acquired ECG, EMG, EEG, and ocular signals with an ECG signal-to-noise ratio of 45.16 dB and a Pearson correlation coefficient >0.85 compared to commercial Ag/AgCl electrodes, establishing signal fidelity suitable for resource-limited biosignal monitoring platforms [283].

Strain-insensitive architectures constructed from bicontinuous PVA-polyvinylpyrrolidone and PEDOT:PSS networks maintained conductivity at 50% strain, suppressing impedance fluctuations during tissue deformation and eliminating motion artifacts in dynamic recordings [287]. Freeze-thaw processing of PVA with tannic acid reinforcement and lithium chloride yielded ion-conductive hydrogels combining superior adhesion strength (2.32 kPa on pigskin), stable conductivity (0.64 S/m), and tensile strength (0.1 MPa) [279]. The optimized PVA-TA-0.5/LiCl-1 formulation achieved exceptional strain sensing fidelity (GF = 1.15) with reliable operational stability through 2500 stretch cycles, enabling simultaneous acquisition of strain and EMG biomarkers (as shown in Figure 10II) [279].

Thermal fluctuations severely degrade performance through thermodynamic softening and electrochemical drift, generating spectral artifacts that compromise signal integrity [34,274,288]. Chen et al. addressed this through thermally adaptive PNIPAM/PVA hydrogels incorporating Hofmeister ion-induced hydrogen bond network reconstruction, maintaining stable EMG acquisition across physiological temperatures (25–40 °C) [34]. This architecture achieved modulus tunability spanning 36.2–3705 kPa while maintaining stable conductivity (6.2–8.2 S/m) across phase transitions, decoupling mechanical compliance from electrical performance. Gesture recognition achieved 100% accuracy with superior signal-to-noise ratios (45.22 dB below LCST, 36–39 dB above LCST), substantially outperforming commercial electrodes, with performance stability extending through ≥30 thermal cycles and reversible skin adhesion (8–12 kPa) compatible with extended wear [34]. However, the restricted operational window (25–40 °C) excludes cryogenic use, and long-term signal drift, wireless integration, and comprehensive biocompatibility evaluation remain necessary before clinical deployment [34].

Underwater surface electromyography demands electrode-skin interface stability, challenging terrestrial bioelectrodes [280,286,289,290]. Wang et al. developed polyphenol-based hydrogels (PTDG) employing dual-locking densification—combining internal ionic locking and solvent-regulated physical crystallization—that suppressed swelling to <3% in water and <9% in seawater while maintaining high ionic conductivity (1.84 S/m) and strong wet adhesion (9.33 kPa) [286]. This architecture enabled sustained minimal impedance drift and high-fidelity sEMG acquisition with a signal-to-noise ratio of 36.47 dB after 60 min of continuous underwater operation. Deep learning-based gesture recognition achieved 97.59% accuracy with a macro-F1 score of 97.09%, alongside precise muscle fatigue monitoring [286]. On the other hand, Deng et al. constructed dual-network hydrogels from copolymerized zwitterionic poly(SBMA–AA) and physically crosslinked PVA that suppressed swelling to 80% equilibrium after 120 h immersion through AA-mediated SBMA protonation [280]. These hydrogels delivered high sensitivity (GF 3.75 across 150–250% strain) with rapid response dynamics (269 ms response, 87 ms recovery), enabling real-time acquisition of physiological (as shown in Figure 10III) and movement-induced signals in human activity monitoring [280].

#### 4.2.3. Electroencephalography (EEG)

EEG recordings capture microvolt-level oscillations from synchronized neuronal populations across the cerebral cortex, directly reflecting ionic currents from synaptic transmission and revealing consciousness state transitions and pathological progression [291,292]. However, EEG acquisition faces dual constraints: microvolt-level amplitude and signal vulnerability to interference compromise measurement fidelity, while current brain–machine interface devices rely on rigid multi-channel electrode arrays, sacrificing portability and wearing comfort [292,293,294].

Chen et al. synthesized physically crosslinked hydrogels from polyvinyl alcohol, gelatin, and MXene nanosheets enhanced with tannic acid (PGTM) via one-pot synthesis [292]. This architecture demonstrated superior anti-drying and anti-swelling characteristics, maintaining stability across diverse aqueous environments (water, saline, acidic, and alkaline solutions) while retaining ~90% conductivity. Low contact impedance (17.18 ± 0.93 kΩ at 10 Hz) and excellent biocompatibility enabled frequency-independent sensing suitable for both subtle motions (finger bending, speech articulation, swallowing) and large-scale deformations in normal and underwater environments, reliably capturing EEG, ECG, and EMG signals [292]. However, excess MXene content may reduce sensitivity through conductive pathway disruption [292]. Zheng et al. synthesized multifunctional sensors via one-pot freeze–thaw gelation combining PVA and sodium alginate with PEDOT:PSS [293]. This architecture achieved a tensile strength of 118.8 kPa, an elongation of 334%, a conductivity of 256 mS/m, and dual-range strain sensitivity (GF = 0.39 for 0–120% and 0.73 for 120–200%) with a 2.2 s response time and >500 cycle stability [293]. Temperature sensing delivered −27.43 Ω/K and 0.729 mV/K sensitivities. These hydrogels successfully captured ECG, EMG, and EEG signals alongside motion monitoring (finger bending, wrist movement, and joint motion) with high skin conformity suitable for wearable health monitoring and flexible electronics (as shown in Figure 11I) [293].

#### 4.2.4. Cardiorespiratory Monitoring

Vigorous physical activity-related fatalities (e.g., marathon) frequently stem from undetected cardiovascular and respiratory abnormalities [295,296]. Commercial wearable devices (smart watches) suffer from fundamental limitations: restricted physiological data (basic heart rate and respiration only), motion artifacts from limb movement during dynamic exercise, mechanical discomfort from excessive tightness, and bacterial proliferation in humid environments. Conformal conductive hydrogel sensors address these constraints, enabling precise, real-time cardiac and respiratory monitoring during prolonged exercise while maintaining skin compatibility and environmental stability—critical requirements for early detection of acute cardiovascular or respiratory failure during athletic events [297].

Wang et al. synthesized a dual-network hydrogel via MXene nanosheets and borax crosslinking of polyvinyl alcohol and tempo-oxidized cellulose nanofibers [295]. The PBTM-3 hydrogel exhibited a tensile strength of 286 kPa, 800% elongation, and high strain sensitivity (GF of 7.79 at 800% strain) with a minimum baseline resistance of ~3 kΩ. The PBTM-3 hydrogel also showed exceptional environmental stability lasting 35 days at 4 °C and 30% relative humidity, with >99% antibacterial efficacy against *E. coli* and *B. subtilis*. Under simulated marathon conditions (38 °C, 52% RH), the sensor maintained stable performance for 6 h and reliably monitored heart rate (82–125 bpm) and respiratory rate (22–39 cycles/min) via carotid artery pulse and chest motion detection (as shown in Figure 11II). The hydrogel also enabled finger bending angle detection and movement monitoring. The primary limitation is MXene aggregation at excess concentrations, which impedes electron transport. This platform is particularly suited for continuous real-time health monitoring in athletes, endurance sports medicine, and extreme environmental conditions [295].

Table 5 compares representative PVA-based hydrogel systems for physiological monitoring and their reported performance.

### 4.3. Environmental and Stimulus-Responsive Sensing

This section groups PVA-based hydrogel systems according to their responses to humidity, temperature, pH, and light. This stimulus-based organization includes both environmental sensing and applications relevant to physiological monitoring. The discussion emphasizes the response mechanism and operating conditions of each system, whereas Section 4.4 focuses on the detection of specific biochemical analytes.

#### 4.3.1. Humidity Sensors

Simultaneous temperature and humidity monitoring constitutes a critical prerequisite for human health protection as well as cultural heritage preservation. Excessive humidity (>80%) accelerates cellulose hydrolysis in archival materials, degrading fiber networks and precipitating brittleness, cracking, and structural failure. Insufficient humidity (<45%) triggers complementary damage through accelerated water evaporation, destabilizing cellulose architecture and inducing mechanical fragility. Relative humidity below 45% increases susceptibility to respiratory tract infections and nasal congestion, whereas relative humidity above 80% impairs thermoregulation and thermal comfort. This dual imperative—safeguarding material integrity and ensuring physiological homeostasis—necessitates integrated, multifunctional sensors capable of simultaneous, high-fidelity temperature and humidity detection across heritage conservation and daily-life applications [40,45,298]. PVA-based hydrogel platforms address this need through five convergent strategies—ionic-liquid fortification, metallic-composite dual-networks, biopolymer cross-linking, hierarchical anisotropy, and crystalline phase transitions—each optimizing distinct environmental or operational constraints.

For instance, Du et al. established transparent ionogel architectures employing vanillin-modified PVA (VPVA) and ionic liquids, which fortify hydrogen bonding with ionic liquids and resolve instability and mechanical fragility inherent to unmodified systems (as shown in Figure 12I). The resulting transparent sensor achieved linear humidity response (R^2^ = 0.9969) across 8–90% RH with negligible hysteresis (<3.86%) and sustained stability across −20 to 80 °C. However, moderate sensitivity (~±0.5–1% RH) and limited strain-responsiveness motivated the transition to multi-responsive designs [299].

To expand operational ranges and simultaneously amplify humidity sensitivity beyond ionic-liquid baselines, Li et al. synthesized dual-network PVA/PAM/LiCl/MXene hydrogels via glycerol-water immersion, achieving exceptional elongation (~1700%), high conductivity (1.6 S/m), and dual sensing mechanisms: above 70% RH, Li^+^-mediated ion transport and MXene metallic conductivity activate (−1.09/% RH), while below 70% RH, surface hydrogen bonding dominates [300]. Figure 12II shows the humidity sensitivity response curve of the PVA/PAM/LiCl/MXene hydrogel sensor. Hydrogen bonding sustains electrical stability under cyclic strain and humidity fluctuations, critical for reliable physiological signal monitoring. This advance in sensitivity and mechanical coupling, however, introduces operational complexity and narrower optimized windows, necessitating alternative design paradigms for broader environmental applicability [300].

Biopolymer-crosslinking strategies decouple mechanical robustness from ionic transduction. Xu et al. synthesized double-network PVA/HPMC/Agar with citric acid and glycerol optimization, achieving a stress of 1.77 MPa, an elongation of 766%, and fatigue resistance over 200 cycles, with a GF of 5.94 at 200% strain across 37–99% RH [301]. This approach trades ionic-conductivity amplification for sustainable synthesis and extended RH ranges, addressing applications prioritizing environmental footprint over peak sensitivity [301].

Heritage conservation and physiological monitoring require simultaneous temperature-humidity sensing, demanding integrated architectures beyond purely humidity-optimized designs. Wang et al. developed conductive PVA-based hydrogels (PSBMA_1_-PMAZ_1.5_) incorporating phytic acid, hydroxypropyl methylcellulose, zwitterionic SBMA, and zinc chloride, establishing stable electrical response across 23.4–56.8 °C and 44–69% RH through synergistic mechanisms: phytic acid hydrogen bonding, SBMA’s amphoteric contributions, and zinc chloride’s salting-out effect [40]. The platform achieved a maximum strain of 739%, a stress of 2.694 MPa, a gauge factor of 1.1 at 0–600%, and a hysteresis ≤ 11.68%, enabling real-time human motion and temperature-humidity response through 200 loading-unloading cycles [40]. But the narrowed supercapacitor operational windows (25–40 °C), composition optimization complexity, and hygroscopic salt loading sensitivity—constraints motivating further innovation in mechanical-thermal decoupling.

Hierarchical anisotropic architectures establish an alternative strategy to purely chemical cross-linking, coupling mechanical robustness directly to nanostructural organization rather than ion-transport modulation alone. Li et al. synthesized loofah-inspired polypyrrole-coated bacterial cellulose–MXene–PVA (PBMA) hydrogels via directional freeze-thaw and supramolecular self-assembly, achieving 1.8 MPa fracture stress, ~6 MJ/m^3^ toughness, 250% stretchability with negligible hysteresis, and >5000 cycle fidelity [302]. Electromechanical sensitivity reaches 4.66-GF (160 ms response/recovery); humidity sensing spans 10–80% RH with 10.2/8.6 s response/recovery times driven by MXene-mediated water adsorption–desorption. The platform integrates multimodal sensing (strain + humidity), strong adhesion, and antimicrobial activity with >90% performance retention after recycling. However, humidity-strain coupling under deformation requires constant-deformation conditions for accurate RH quantification. Applications including respiration tracking, facial expression detection, arterial pulse monitoring, joint bending discrimination, and gesture recognition (99.75% accuracy) establish this platform for wearable electronic skins and comprehensive health monitoring [302].

Phase-transition-driven hydrogels could leverage crystallization kinetics to achieve exceptional mechanical enhancement while simultaneously introducing piezoelectric functionality—a capability absent from water-mediated ionic transport systems. Liu et al. synthesized inhomogeneous salt hydrogels comprising PVA, PAM, and lithium nitrate trihydrate with crystal-seed-triggered phase transitions, enhancing tensile strength from 31.5 to 326 kPa and establishing surface-rich crystal layers [303]. This restructuring delivers linear RH relationships in electrical conductivity and piezoionic voltage, reversible adhesion (295 kPa on porcine skin, 263 kPa on wood) persisting across thermal cycles, high Li^+^ conductivity, and response/recovery times of 77/75 ms. However, hygroscopic salt loading dependence may limit extended operation across diverse climatic environments [303].

Table 6 compares representative PVA-based hydrogels for humidity and temperature sensing.

#### 4.3.2. Temperature Sensor

Static physiological temperature information complements dynamic mechanical stimuli in health assessment. Body temperature maintenance at 36.3–37.2 °C means deviations indicate fever or physiological abnormalities; sensors detecting abnormal increases from 34 to 42 °C aid disease and inflammatory response assessment. Additional capability to detect nearby heat or cold sources alerts users to thermal burn or frostbite risks, enhancing safety and environmental awareness [304,305,306,307,308,309].

**Table 6:** Summary of representative PVA-based hydrogels for humidity and temperature sensing.

Hydrogel Name	Conductive Component	Sensing Range	Key Advances	Main Application	Ref.
Vanillin-Modified PVA (VPVA) ionogel with [EMIM][BF_4_] ionic liquid	[EMIM][BF_4_]	Humidity Range: 8–90% RH	1. Exceptional linearity and minimal hysteresis (<3.86%); 2. Superior mechanical integrity; 3. Intrinsically transparent and robust	1. Non-contact skin humidity sensing;	[299]
PVA/PAM/LiCl/MXene	LiCl/MXene	Humidity sensitivity (−1.09/% RH)	1. Multifunctional sensing	1. Human health monitoring	[300]
PVA/HPMC/Agar (with citric acid crosslinking)	citric acid	Humidity: 37–99% RH; Strain sensing: GF = 5.94	1. Superior mechanical properties; 2. Excellent fatigue resistance and good recovery after cyclic loading;	1. Human motion detection; 2. Wearable medical and bioengineering applications	[301]
PBMA: PVA/Tannic Acid/Ppy@Bacterial Cellulose/MXene	Polypyrrole@Bacterial Cellulose fibers + MXene	-Strain sensing: GF = 4.66; -Humidity sensing: 10–80% RH; -Strain range: up to 250%	1. Superior multimodal performance; 2. Fast electromechanical response/recovery (~160 ms); 3. Exceptional durability; 4. Thermal reprocessability with >90% original sensing performance retention; 5. Recyclable and sustainable design	1. Environmental humidity sensing; 2. Physiological motion monitoring	[301]
PVA/PAM/LNH	LNH	Linear output voltage response across relative humidity range	1. Innovative Salt-Hydrogel Design; 2. Reversible Phase Transition; 3. Multi-Modal Sensing Capability; 4. Moisture Retention Without Dehydration	1. Smart wearable devices; 2. Humidity sensing; 3. Energy harvesting	[301]
PVA/CPBACMC@PANI)	PANI	-Strain sensing: GF = 0.75/1.88; -Temperature sensing: TCR = −1.52%/°C) in 20–60 °C; -Response/Recovery time: ~200 ms	1. Superior mechanical properties; 2. Dual-responsive sensing; 3. Fast self-healing ability; 4. Excellent cyclic stability	1. Wearable electronics and healthcare monitoring; 2. Continuous body temperature monitoring	[307]
PVA/SA/PEDOT:PSS (modified with dilute H_2_SO_4_)	PEDOT:PSS	-Strain Sensing: 0–200% range (GF = 0.46 at 0–100%; GF = 0.57 at 100–200%); -Temperature Response: −2.43 U/K; 7.91 mV/K;	1. High Toughness and Mechanical Durability; 2. Multifunctional Sensing: Decoupled strain/temperature response. 3. Fast Response: 100 ms	1. Wearable Health Monitoring; 2. Body temperature monitoring	[308]
PVA/PEDOT:PSS/LiCl	PEDOT:PSS + LiCl	-Strain Sensing: 0–16 kPa pressure range; -Impedance change ΔR/R~0.07–11.3%; -Thermoelectric Performance: Temperature detection down to 0.1 K sensitivity;	1. Superior Anti-Swelling; 2. Exceptional Fatigue Resistance; 3. Dual-Mode Sensing (Temperature + Strain); 4. Three-dimensional-Printability and Biocompatibility	1. Real-time dual sensing of temperature and tissue deformation; 2. Wearable Health Devices	[8]

For instance, Zhang et al. synthesized dual-responsive PVA/CPBA/CMC@PANI hydrogels exploiting dynamic borate-bond and hydrogen-bond crosslinking, achieving a negative thermal coefficient of resistance (TCR): −1.52%/°C (20–60 °C) and −0.303%/°C (60–80 °C), enabling thermal resolution ≤ 0.5 °C through borate-bond rearrangement coupled with PANI’s temperature-dependent conductivity [307]. Concurrent strain sensing achieves a gauge factor of 1.88 with ~200 ms response time, enabling joint movement tracking, facial expression capture, and continuous body temperature acquisition within integrated wearable systems [307]. Zhou et al. integrated acid-modified PEDOT:PSS into PVA/sodium alginate networks via electrostatic interactions and hydrogen-bonded cross-linking (coded as PSPSH), establishing electrical conductivity of 48.69 mS/m, fracture strength of 45.38 kPa, ductility of 209.13%, and simultaneous fatigue resistance [308]. Strain detection reaches the 2.5% threshold alongside dual-mode temperature response (−2.43 Ω/K resistance sensitivity, 7.91 mV/K voltage sensitivity), establishing robust body temperature monitoring for extended wearable deployment [308]. Figure 13 shows the temperature sensing characteristics of PSPSH.

PVA/PEDOT:PSS patches incorporating regulated Li^+^/Cl^−^ ion diffusion achieve dual-mode wound temperature and strain sensing through Soret-effect-driven thermopower (3.56 mV/K) and sub-kelvin sensitivity (0.1 K detection) [8]. Mechanical resilience reaches ~88 kPa tensile strength and 331% elongation, with exceptional signal decoupling (35% thermal versus 3.9% strain impedance change) enabling independent quantification under dynamic conditions. Superior anti-swelling stability and waterproof operation sustain >90% thermoelectric performance after 96 h continuous monitoring, positioning the patch for early-stage wound infection detection in chronic wound management and diabetic ulcer assessment [8]. Figure 14 shows the integrated applications of these wound monitoring patches.

Biocompatible dual-network hydrogels incorporating PVA, poly(NIPAM-co-acrylamide), eutectic gallium-indium/MXene-polyaniline composites (~6 wt%), and chitosan achieve skin-matched mechanics (Young’s modulus ~140 kPa), stretchability with resistance changes exceeding 1000%, and a gauge factor of 2.5 at 400% strain [310]. Multimodal sensing establishes temperature sensitivity of −2.9%/°C (20–40 °C) and 65%/°C (0–20 °C), alongside pH responsiveness of −13.68 mV/pH for simultaneous biophysical and biochemical monitoring. Gallium and chitosan constituents confer antimicrobial capacity (>99% bacterial killing under infrared) and triboelectric energy harvesting (83 mW/m^2^ at 40 N load). The integrated platform decouples mechanical compliance from multifunctional responsiveness—strain, temperature, pH, and energy harvesting—across joint tracking, swallowing assessment, gesture recognition, and AI-integrated health analysis [310].

These platforms decouple mechanical compliance from electrochemical sensitivity through dynamic cross-linking, ion transport modulation, or structural hierarchy—capturing temperature and strain through orthogonal mechanisms (TCR + gauge factor, Soret effect + elasticity, PANI conductivity + borate rearrangement). Trade-offs emerge: TCR-dominant systems achieve superior thermal resolution (≤0.5 °C, 0.1 K) but modest strain sensitivity (GF = 1.88–2.5); ion-transport platforms excel at signal decoupling yet require moisture-stability encapsulation; multifunctional designs maximize capability (temperature + pH + energy) at increased complexity cost. Future advancement demands standardized biocompatibility protocols, long-term signal drift characterization under physiological conditions, and wireless integration for autonomous wearable deployment.

#### 4.3.3. pH Sensor

Beyond environmental temperature monitoring, biofluid composition analysis through pH-responsive hydrogels adds an orthogonal biochemical dimension to comprehensive physiological assessment. Whereas temperature sensors quantify thermal state through resistance modulation, pH-responsive platforms detect biochemical markers through network swelling, ionic conductivity changes, and crosslink dissociation. This metabolic indicator—pH shifts in sweat (4.5–6.5), wound exudate, and bodily secretions—signals infection, metabolic imbalance, or inflammatory response, establishing pH detection as an essential biomarker alongside thermal and mechanical parameters.

Physiological pH fluctuations across biological fluids (sweat, wound exudate, secretions) necessitate pH-responsive hydrogel sensors enabling simultaneous biochemical and biophysical monitoring [311,312,313,314]. For instance, Fu et al. developed an acrylic acid (AA)/hydroxyethyl methacrylate (HEMA)/sodium styrenesulfonate (SSS) terpolymer in PVA that achieves 810% elongation, 0.31 MPa tensile strength, 1.48 MJ/m^3^ toughness, and 0.03 MPa Young’s modulus through AA’s pH-responsiveness, HEMA’s microhydrophobic domain balance, and SSS’s conductivity. Strain sensing achieves a GF of 7.12 with 0.26/0.28 s response/recovery times, enabling joint movement monitoring with satisfactory biocompatibility for dual motion detection and transdermal drug delivery applications [315]. Yan et al. reported a multifunctional PPMP@S/ApoVs platform—incorporating PEDOT:PSS, polypyrrole, MXene nanosheets, and stem cell-derived apoptotic vesicles (2 μg/mL)—establishes integrated wound monitoring and on-demand drug delivery for diabetic healing. Synthesized via sequential PVA mixing, in situ aniline polymerization, freeze-thaw cycles, and borax crosslinking, the platform demonstrates excellent electrical conductivity, hemolysis <5%, >80% bacterial killing (*S. aureus* and *E. coli*), effective reactive oxygen species scavenging via MXene nanozymes, and M1-to-M2 macrophage polarization with enhanced CD206 expression. Dynamic pH responsiveness across 4.5–6.5 (characteristic of diabetic wounds) operates through reversible borate ester bond formation: acidic conditions convert B(OH)_4_^−^ to H_3_BO_3_, disrupting crosslinks and triggering accelerated bioactive substance and apoptotic vesicle release [50]. Figure 15 shows the pH-responsiveness of ApoVs.

Triple physical cross-linking (TPC) hydrogels from poly(vinyl alcohol) and sodium alginate via F/T cycles and Ca^2+^ soaking establish three synergistic pathways—hydrophobic association, microcrystallinity, and ionic coordination—decoupling mechanical robustness from strain sensitivity while maintaining skin-matched compliance (33–45 kPa) [316]. The material achieves a fracture energy of 5.9 MJ/m^3^, stretchability of 2490%, electrical conductivity of 0.65–2.47 S/m, and a maximum gauge factor of 10.29 across 580% strain. pH-responsive carboxylic acid groups modulate ion migration and network resistance across physiological pH ranges, with sensing stability persisting through 50+ consecutive stretching-unloading cycles and reproducibility over 100 successive cycles, supporting reliable wearable health monitoring and human movement detection. However, trade-offs between PVA/Ca^2+^ ratios and strain-mechanical sensitivity, coupled with pH-responsiveness variability in complex biological fluids, warrant further investigation [316].

A PNIPAM/GO/PVA/Na^+^ hydrogel incorporating thermosensitive poly(N-isopropylacrylamide), pH-responsive graphene oxide, and Na^+^ ions achieves dual-stimuli responsiveness through PNIPAM’s lower critical solution temperature behavior and GO’s dissociable carboxyl groups [317]. Temperature sensing achieves −2.23%/°C sensitivity (29.2–43.9 °C range, 0.1 °C resolution, 321 ms response); pH sensing delivers 53.08%/pH sensitivity (pH 1.00–9.18, 481 ms response). The dual-modal configuration achieves ultra-wide linearity (R^2^ = 0.9953), enabling simultaneous real-time biophysical and biochemical monitoring, though signal interference from complex sweat composition and substrate-integration constraints may require optimization [317].

A sustainable conductive hydrogel (CCP) synthesized via physical crosslinking of polyvinyl alcohol with coal-based carbon quantum dots and carbonized lignin eliminates toxic chemical crosslinkers while enabling dynamic network restructuring [318]. Surface carboxyl groups confer pH responsiveness across physiological pH 4.0–7.0 for real-time sweat pH monitoring, coupled with exceptional mechanical performance (7.5 MPa tensile strength, 487% elongation, 24.5 mS/m conductivity) and strain sensing (gauge factor 5.34). The platform leverages renewable and waste-derived materials through green physical crosslinking to establish multimodal responsiveness—simultaneous motion detection and biochemical marker sensing—positioning it for wearable health monitoring in sports medicine and metabolic assessment [318].

Table 7 summarizes and compares representative PVA-based pH-responsive hydrogels as multifunctional platforms for integrated biochemical and biophysical monitoring.

#### 4.3.4. Photoresponsive Sensor

The preceding environmental sensors—humidity, temperature, and pH—establish passive detection through inherent material responsivity to ambient conditions. Photoresponsive platforms introduce a fundamentally different operational paradigm: active, externally triggered modulation of mechanical and electrochemical function through light stimulation. This capability enables remote, contact-free manipulation without physical contact, transcending passive-sensing limitations and establishing bidirectional control architectures essential for autonomous soft robotics, implantable devices, and adaptive wearables that require both information acquisition and programmed actuation.

Photoresponsive conductive hydrogels integrate light-triggered functionality with electrical transduction, enabling remote, contact-free manipulation of mechanical and sensing properties in real time. This convergence addresses a critical engineering challenge: simultaneously achieving rapid reversible actuation, mechanical robustness, and multimodal sensing—capabilities essential for autonomous soft robotics, implantable biodevices, and adaptive wearable electronics [319,320,321,322]. Light-triggered mechanisms enable precise spatial and temporal control while maintaining electrical conductivity for real-time signal monitoring, transcending single-modality design limitations [49,323,324].

For instance, Guo et al. developed a PNIPAM/AM/PVA hydrogel reinforced with MXene nanosheets as broadband photon absorbers. Under NIR irradiation (808 nm), MXene converts photons to thermal energy, triggering phase transition near the lower critical solution temperature and enabling rapid directional bending with 34.89% volume shrinkage within 50 s. The composite achieved Young’s modulus of 13 kPa and tensile strain of 371.98%, with an electrical conductivity of 0.06 S/m. Integration of a piezoelectric composite layer [poly(vinylidene fluoride-trifluoroethylene)/barium titanate] enabled multimodal sensing: piezoresistive detection (GF = 1.78; response/recovery 235/292 ms) and piezoelectric sensing (45 ms response time) captured strain magnitude, bending direction, and deformation velocity [323].

NIR light penetrates tissue with minimal damage and enables precise spatial patterning without physical contact. Xie et al. developed a mussel-inspired indocyanine green–polydopamine-reduced graphene oxide–calcium phosphate–PVA (ICG-PGO-CaP-PVA) nanocomposite targeting advanced wound dressings through photothermal-photodynamic synergy. Polydopamine-reduced graphene oxide loads ICG while amorphous calcium phosphate nanoparticles provide mechanical robustness and biocompatibility. Under NIR irradiation (808 nm), dual mechanisms operate synergistically: photothermal effects elevate temperature to ~52 °C within 5 min enabling direct antibacterial heating; photodynamic effects generate reactive oxygen species. Bacterial survival reduced to 5.6% (*S. epidermidis*) and 11.3% (*E. coli*)—17- and 9-fold improvements over pure PVA. Photothermal-induced gel-sol conversion triggers controlled Ca^2+^ release, facilitating multi-phase wound healing. Mechanical properties (tensile strength 1.4 MPa, 253% strain, 0.9 MPa compressive strength, 80% recovery) enable clinical translation in bacterial-infected injuries [324].

Photoisomerization pathways exploit azobenzene-based crosslinkers undergoing UV-vis-triggered cis-trans isomerization without thermal effects. Boronic acid–azobenzene crosslinkers integrated with catechol-functionalized single-walled carbon nanotubes (SWCNTs) demonstrate light-controlled electrical switching. Reversible photoisomerization triggers molecular-level conformational changes propagating to macroscopic network rearrangement and consequent conductance modulation. At 5 wt% SWCNT loading, conductivity reaches 3.5 S/cm with photoresponsive conductance switching between 10 and 60 µA under alternating UV and visible light irradiation. The 3D crosslinked network, formed through boronic ester and hydrogen bonding, maintains immediate self-healing and viscoelastic behavior, enabling contact-free switching applications and soft actuators requiring precise, reversible deformation control [49].

Multi-stimuli variants expand the design landscape. Temperature/ethanol/salt-responsive hydrogels (PPSD) exploit contrasting hydrophilic-hydrophobic monomer structures, achieving reversible light-scattering transitions: polymer chain aggregation at 60 °C induces transparent-to-opaque conversion, recovering within 5 s upon cooling. Electrical conductivity ranges 0.01–0.80 S/m, with piezoresistive strain-sensing reaching 73% resistance change at 100% tensile strain and stable performance over 100 stretch cycles [325]. Dual UV/mechanical stress-responsive wearables (PVA-GEL-GL-Mo-7) deliver 2.2 MPa tensile strength, 5.26 MJ/m^3^ tenacity, 522% extensibility, and 90% transparency across −50 to 85 °C, enabling concurrent ambient light variation and mechanical loading monitoring for wearable health monitoring in diverse climates [326].

Table 8 compares representative PVA-based photoresponsive conductive hydrogels discussed in this section.

### 4.4. Selected Applications in Biochemical Sensing

Conductive PVA-based hydrogels enable real-time biochemical detection through charge-transfer reactions triggered by target analytes, establishing flexible biosensing platforms combining precise structural control, compositional versatility, and programmable architectures for next-generation wearable diagnostics [328,329].

This section discusses selected biochemical sensing applications of PVA-based hydrogel platforms rather than providing an exhaustive survey of biosensing. Particular attention is given to glucose and other biomarker detection, including multiplexed analysis and selected examples involving alternative biofluids. The aim is to connect material design with analytical function and practical sensing requirements.

#### 4.4.1. Glucose Monitoring in Sweat

Non-invasive glucose monitoring through sweat sampling addresses a critical clinical need in diabetes management, where frequent assessment prevents hyperglycemia-induced complications. Sweat provides an accessible biofluid containing diverse metabolic biomarkers—glucose, lactate, and urea—enabling continuous metabolic status assessment via direct skin-surface sampling [29,58,330,331,332,333]. Maintaining physiological glucose concentrations through neural and endocrine homeostasis is essential for preventing metabolic dysfunction and systemic complications [330,334].

PVA-based enzymatic glucose sensors leverage tunable hydrogel composition to achieve picomolar limits of detection. For instance, Devi et al. demonstrated that PANI-to-PVA ratios govern electrical and mechanical properties; optimized blends achieve LOD ~0.001 μM, though oxidation/reduction currents decline at elevated glucose concentrations under high electrolyte conditions, indicating saturation limitations [335]. Pan et al. engineered PVA/PEDOT:PSS films with integrated ethylene glycol and fatigue-resistant Ni nanoparticles, overcoming enzyme-based instability through dual-phase conductivity enhancement under dynamic strain, achieving LOD 0.37 μM and sensitivity 262.17 μA mM^−1^ cm^−2^ with exceptional long-term stability [336].

Advancing platform robustness, a Janus CMC/PVA hydrogel integrates high sensitivity, skin adhesion, and sub-zero operability through asymmetric layer design: a glycerol-modified outer layer (−38 °C operation) and a polydopamine-coated inner layer (enhanced adhesion). Glucose oxidase-modified gold nanorods achieve electrochemical detection across 10–350 μM with LOD 0.21 μM [337]. The MXene-PAM-PVA-GOx platform extended this further through supramolecular integration of MXene, glucose oxidase, gold nanoparticles, and Prussian blue, achieving wide-range detection (0–5.3 mM, LOD 9 μmol) with three-week continuous skin monitoring—substantially exceeding commercial device lifespans [338]. Figure 16 presents the blood glucose measurement on skin. However, a remaining limitation persists: enzymatic glucose sensors exhibit reduced sensitivity above 70% relative humidity, necessitating enhanced hydrogel design for enzymatic stability under high-moisture conditions to achieve environment-independent monitoring [338]. Panels (A) and (C) show photographs of the GM3PAS patch on rabbit and human skin, respectively. Panels (B) and (D) show the corresponding invasive glucose meter measurements from rabbit ear vein and human fingertip blood samples. Panel (E) correlates current values from rabbit skin surface glucose assays with blood glucose meter readings, while panel (H) presents the equivalent correlation for human skin surface assays. Panels (F)–(G) and (I)–(J) display continuous glucose monitoring curves obtained from the GM3PAS patch over multiple time points in rabbit and human subjects (two investigators), respectively.

#### 4.4.2. Multiplexed Biomarker Detection

Beyond single-analyte glucose monitoring, multiplexed PVA platforms simultaneously detect multiple biomarkers and biophysical parameters within extended physiological ranges [339,340]. Uric acid detection exemplifies this capability: Lou et al. developed a multifunctional organohydrogel combining carbon nanofibers, MXene, and PEDOT:PSS-impregnated PVA/PAAM, achieving a linear detection range of 20 μM–14 mM with LOD 10.93 μM, dual-region sensitivities (0.0247 and 0.0064 µA·µM^−1^·cm^−2^), 94.2% signal retention after 35 days, and integrated temperature sensing (16–40 °C); real sweat validation yielded recovery rates of 98.81–101.22%, establishing clinical viability for hyperuricemia diagnosis and gout management [341]. Complementing this, Gao et al. synthesized a ternary PEDOT/PVA/MXene hydrogel via hydrogen-bonded assembly, where PEDOT provides electronic conduction, PVA provides matrix toughness, and MXene enhances conductivity and electrocatalytic activity [4]. This architecture expands the electrochemical active area to 0.7973 cm^2^, achieving uric acid sensitivity of 0.289 μA·µM^−1^·cm^−2^ with LOD of 0.57 μM across physiological range 1–400 μM, maintaining stable performance under mechanical deformation and tracking dietary-induced fluctuations with ELISA-equivalent accuracy [4].

Extending beyond sweat to alternative biofluids expands diagnostic scope to stress biomarkers inaccessible through sweat alone [342,343,344]. Parth et al. fabricated PVA–PEGDA–SA composite hydrogels via porogen leaching (10–20 µm pores) and electrospinning (0.2–0.4 µm interconnected nanofibers) to overcome biofouling, reduced sensitivity, and mechanical instability in conventional coatings. Electrospun membranes substantially outperform leached variants through suppressed baseline noise, controlled swelling (<100% vs. >600% over 24 h), and stable analyte diffusion, establishing the fabrication route as a critical design parameter for predictable electrode interfacial behavior and enabling saliva-compatible point-of-care diagnostics. Figure 17 shows the biosensing performance of these hydrogels [345]. Panels (A) and (B) show the sensor configuration and digital photograph. Panel (C) compares current response profiles upon exposure to 0.1 µg/dL cortisol for three configurations: no hydrogel membrane, leached membrane, and electrospun membrane with dye. Panels (D) and (E) demonstrate dose-dependent performance: panel (D) shows chronoamperometric responses across increasing cortisol concentrations (0–10 µg/dL), and panel (E) presents the corresponding calibration plots recorded at 50, 200, and 400 s timepoints. All measurements were conducted in triplicate (n = 3) with reported data as mean values ± standard deviations.

Table 9 compares representative PVA-based hydrogel systems for selected applications in biochemical sensing. Comparisons should distinguish measurements in standard solutions from those in artificial sweat, authentic biofluids, and on-body tests. Future evaluations should report selectivity, calibration stability, and the effects of sample conditions alongside sensitivity and detection limits. This would help establish whether analytical performance is retained under the intended conditions of use.

## 5. Challenges, Limitations, Future Perspectives, and Conclusions

### 5.1. Current Challenges and Limitations

Despite remarkable progress in developing conductive PVA-based hydrogels for wearable bioelectronics, significant technical and practical barriers continue to impede their transition from laboratory prototypes to clinically deployable systems. These challenges span multiple dimensions of material science, manufacturing, and clinical translation [20,346].

Firstly, the fundamental tension between enhancing electrical conductivity and maintaining hydration stability remains unresolved. Electron-conducting systems utilizing carbon nanofillers (CNTs, graphene, MXene) achieve rapid response and high sensitivity for strain sensing applications, but may exhibit cyclic instability and hysteresis associated with changes in filler contacts and deformation of the polymer network [42,56,302,347]. Conversely, ion-conducting systems leverage the excellent mechanical compliance and skin-conformability of hydrated PVA networks for bioelectrode applications but remain vulnerable to dehydration-induced changes in conductivity during extended wear unless hydration is adequately controlled [222,348,349,350]. Hybrid-conduction approaches theoretically mitigate these individual limitations; however, material complexity introduces new challenges—filler aggregation, phase segregation under mechanical stress, and compromised interfacial stability—that restrict deployment in high-performance devices [210,248,303].

In addition, incorporating inorganic nanofillers into organic polymer matrices presents persistent interfacial compatibility challenges. Leakage of dopants and conducting fillers into aqueous environments, aggregation phenomena driven by van der Waals forces in aqueous dispersion, and poor mechanical coupling at filler-polymer boundaries collectively compromise structural uniformity, electrical performance, and long-term stability [215,346,351]. Surface-embedded conductive networks deposited directly onto polymer substrates offer a promising design direction by decoupling the conductive phase from bulk matrix transport, but scaling this approach to industrial production remains technically challenging [352,353]. Reproducibility requires control of processing history as well as nominal composition. Meng et al. showed that, at a PVA content of 12.5 wt%, the fracture strength of a PVA/borax/ionic-liquid hydrogel increased from approximately 0.5 MPa without freeze–thaw treatment to 3.1 MPa after three cycles, which they attributed to improved network organization and energy dissipation. This comparison demonstrates substantial processing sensitivity, rather than directly establishing poor batch-to-batch reproducibility [249]. It nevertheless identifies freeze–thaw history as a critical variable when reproducing a formulation. A useful distinction is therefore between the repeatability of measurements on one specimen and reproducibility across independently prepared batches. We recommend reporting the number of independent batches and the within- and between-batch variation in conductivity and mechanical properties under matched conditioning and testing conditions.

Robustness should likewise be distinguished from high strength in a single tensile test. In the same study, increasing the PVA content above 12.5 wt% increased tensile strength but reduced elongation and toughness; loading–unloading tests at strains of 50–300% also exhibited pronounced hysteresis. These findings show that strength, extensibility, toughness, and recovery cannot be treated as interchangeable measures of durability [249]. Mechanical hysteresis alone does not establish electrical drift, but it motivates simultaneous mechanical and electrical measurements during repeated deformation. For wearable sensors, we therefore recommend tracking residual strain, hysteresis, baseline resistance, and response amplitude over repeated cycles, with strain amplitude, loading rate, recovery interval, temperature, and hydration state specified. Such measurements would help distinguish a mechanically strong material from a sensor that maintains a reproducible response during use.

To improve durability, hydration control should be combined with network and interface stabilization. PVA crystalline domains can provide physical anchoring points, while secondary polymer networks and reversible interactions can reinforce the matrix and facilitate structural recovery [173,274]. In conductive composites, improved filler–polymer coupling is also important for maintaining conductive-pathway continuity during repeated deformation. These measures should be optimized alongside hydration management, with attention to the balance between mechanical reinforcement and the compliance required for conformal skin contact [1,21,354].

The inherent hygroscopic nature of PVA hydrogels—while advantageous for biocompatibility—creates vulnerability to environmental moisture fluctuations. Prolonged exposure to dry environments (relative humidity < 30%) triggers progressive water loss from the hydrogel network, inducing crystallization, mechanical embrittlement, and catastrophic conductivity degradation [1,166,295,355]. Extended use in physiological conditions (sweat, wound fluid) alters local ionic concentrations and causes uneven ion dilution, further compromising both ionic conductivity and structural cohesion [8,166,356]. While advanced approaches incorporating deep eutectic solvents and encapsulation strategies have partially mitigated these issues, robust solutions addressing long-term biocompatibility, manufacturing scalability, and environmental cost remain underdeveloped [167,168,357].

Thermal fluctuations severely degrade sensor performance through both thermodynamic softening of the hydrogel matrix and electrochemical drift in the conductive phase. Current designs exhibit restricted operational windows (typically 25–40 °C for physiological applications), excluding cryogenic scenarios and limiting utility in extreme environmental conditions [34,35,40,317]. Long-term signal drift—where electrochemical potential gradually shifts over hours or days of continuous monitoring—remains inadequately addressed and threatens the reliability of extended diagnostic applications [2,20,358,359]. Even thermally adaptive designs achieving stable EMG acquisition across physiological ranges require reversible structural rearrangement (hydrogen bond reconstruction), introducing concerns about fatigue and reproducibility over thousands of thermal cycles [34,302,360].

Moreover, moving beyond single-parameter sensors toward multimodal, multiplexed systems demands sophisticated integration of diverse sensing transduction mechanisms, signal conditioning circuitry, and wireless communication modules onto flexible substrates [257,304,310]. Power management presents a critical unresolved challenge: most wearable systems currently require either frequent charging (impractical for continuous monitoring) or miniaturized coin-cell batteries that compromise flexibility and skin conformability. Integrated energy harvesting directly from body heat or mechanical motion has been demonstrated at laboratory scale, but achieving sufficient power density while maintaining electrical compatibility with conductive hydrogel interfaces remains elusive [257,310,361,362].

Integrating conductive PVA-based hydrogels into wearable electronics requires reliable interfaces beyond bulk conductivity alone. Mechanical mismatch between the hydrated network and electronic components promotes interfacial delamination during repeated deformation, particularly at the skin–hydrogel contact (requiring conformal adhesion to minimize motion artifacts) and the hydrogel–electronic connection (requiring stable electrical coupling) [1,20]. Ionically conductive systems present additional interface-design complexity in coupling ionic transport to electronic readout. Integration must be assessed at the assembled-device level during deformation and environmental exposure, including interfacial integrity and signal stability, rather than inferred from standalone conductivity or tensile measurements [17,21,279,352,363].

Scalability and reproducibility are constrained by processing complexity and sensitivity to formulation and fabrication conditions. Freeze–thaw cycling, solvent exchange, and post-treatment prolong production, while maintaining uniform cooling conditions at larger scales generates spatial differences in network formation and nonuniform mechanical and electrical properties. Strict control of PVA molecular weight, degree of hydrolysis, concentration, and freeze–thaw history is essential, as these parameters influence crystallization and network integrity. In conductive composites, uniform distribution of fillers, dopants, and crosslinking agents is particularly challenging; agglomeration and phase segregation compromise structural uniformity while increasing manufacturing costs [364,365,366,367].

These limitations are compounded by insufficiently standardized evaluation across laboratories. To improve comparability, studies should systematically report raw-material specifications, complete formulation and processing histories, specimen dimensions, hydration state, and measurement conditions. Reproducibility must be evaluated using independently prepared batches with explicit reporting of batch variability in conductivity, mechanical properties, and device-level performance; repeated measurements of a single specimen do not constitute evidence of batch-to-batch reproducibility. Accordingly, simplified processing should incorporate defined process windows and batch-level quality control, recognizing that laboratory prototypes do not inherently establish scalable manufacture [1,17,292,293,310].

While PVA itself enjoys excellent biocompatibility credentials, composite systems incorporating nanofillers, conducting polymers, and chemical dopants raise legitimate concerns regarding immunogenicity, cytotoxicity, and long-term in vivo responses [19,353,368]. For skin-mounted devices, evaluation should specifically address irritation and potential additive leaching from the final formulation during prolonged contact, because the biocompatibility of PVA alone does not establish the safety of the integrated device. Current literature predominantly relies on short-term in vitro assays; comprehensive long-term biocompatibility studies—including chronic implantation protocols, comprehensive immune profiling, and biodegradation kinetics—remain underdeveloped. Regulatory pathways for wearable bioelectronics remain nascent, requiring development of standardized testing protocols, biocompatibility documentation, and performance validation benchmarks acceptable to agencies such as the FDA [17,19,61].

### 5.2. Future Perspectives

Addressing these interconnected challenges demands coordinated advances across materials science, device engineering, manufacturing technology, and clinical validation. The following priorities emerge from current literature:

Rather than relying solely on increasingly complex multicomponent formulations, future research should explore hierarchical architectures that allow electrical, mechanical, and biological functions to be tailored at different length scales [38,197,257,369]. Surface-functionalized systems maintaining external conductivity while preserving bulk hydrogel properties merit focused investigation. Bio-inspired composite architectures—including nacre-like hierarchical lamination and gradient interfaces—may achieve simultaneous high conductivity and mechanical compliance without sacrificing stability or biocompatibility [38,223,292,370,371,372]. Machine learning-guided materials discovery, coupling high-throughput synthesis with predictive computational models, could rapidly identify optimal filler concentrations, dopant combinations, and cross-linking parameters for specific applications, substantially accelerating translation [306,373,374,375,376].

Long-term wearable operation requires complementary strategies to retard water loss and preserve electromechanical performance [1,2,166,295,299,377]. At the formulation level, water–polyol solvent systems, such as glycerol/water mixtures, can reduce water vapor pressure and slow evaporation, helping to preserve hydration-dependent compliance and charge transport [1]. As a representative example, Han et al. reported that an ethylene-glycol-containing PANI/PVA/CMC-P(AM-co-AA)-Zn^2+^ hydrogel retained its shape and a visibly moist surface after seven days at room temperature, whereas the corresponding hydrogel without ethylene glycol underwent severe shrinkage [253]. This supports improved resistance to drying but does not by itself establish stable electrical performance during seven days of continuous wear. Humectant content should therefore be optimized rather than maximized, because solvent modification also changes network mechanics. Additive compatibility with prolonged skin contact must also be evaluated [1].

At the device level, hydrophobic coatings or elastomeric barrier layers can complement bulk humectants by restricting evaporation [2]. Barrier coverage should be tailored to the application to balance water retention, skin conformity, and breathability, rather than relying on thick, fully occlusive encapsulation. Together with network and interface stabilization, these approaches offer a practical route toward improved durability. Their effectiveness should be evaluated by measuring mass or water-content retention alongside conductivity or baseline-resistance drift, tensile-property retention, and cyclic hysteresis after aging under specified temperature and relative humidity [2]. Sweat exposure, repeated deformation, and skin–electrode impedance should additionally be considered for wearable bioelectrodes. Storage stability and short cyclic tests should be distinguished from sustained on-skin performance [55].

Further evaluation should consider structural defects and damage–healing cycles alongside hydration stability. Where cracks or exposed pores are present, possible capillary-driven liquid redistribution deserves investigation rather than being assumed negligible. Mechanical recovery, electrical continuity, and sensing performance should be assessed separately after repair. Bulk electrical measurements should also be complemented by structural and interfacial characterization when interpreting charge transport in heterogeneous hydrogel networks.

Developing predictive models of thermal-electrochemical coupling will enable rational design of compensation mechanisms—either through active feedback control of electrode potential or through passive materials engineering wherein thermal sensitivity is intentionally programmed to cancel sensor drift [34,40,302,317]. Extended validation under realistic thermal stress—including prolonged body-temperature operation, diurnal cycling, and extreme environmental exposure—is essential before clinical deployment [7,35,295,310].

Rather than relying on rigid external electronics, future systems should embed flexible signal conditioning directly into hydrogel platforms through molecular-scale electronic design or hybrid organic-inorganic interfaces [7,293,323,329]. Wireless power transfer technologies adapted for bioelectronic applications could enable true wireless operation without bulky batteries. Development of biocompatible wireless communication modules operating at reduced power consumption (achieving multi-day autonomy from thin-film energy harvesting) represents a critical unmet need [197,257,310,378].

The bioelectronics research community should prioritize establishing consensus protocols for material characterization, sensor performance validation, and biocompatibility assessment. Creation of reference standards, interlaboratory comparison studies, and harmonized reporting formats would accelerate technology maturation and facilitate regulatory approval [1,2,7,257]. Close collaboration between academic researchers, device manufacturers, clinical validation partners, and regulatory authorities should be initiated early in development to align technical specifications with eventual clinical requirements.

Transition from prototype demonstrations to clinically validated systems demands rigorous long-term studies in relevant patient populations [295,379]. For continuous physiological monitoring applications, multi-week or multi-month prospective studies comparing conductive PVA-based hydrogel sensors against FDA-approved reference devices would establish clinical utility and safety profiles [17,276,277,380]. For biochemical sensing applications, expanded biofluid sampling (incorporating tears, saliva, and interstitial fluid beyond sweat) and multiplexed analyte detection capabilities should be systematically validated across diverse pathophysiologic states [2,100,331,337,341,345].

Given the growing emphasis on sustainable materials, incorporating biomass-derived components (chitosan, cellulose, and plant polyphenols), renewable cross-linking agents, and green solvents (deep eutectic solvents and ionic liquids) into conductive hydrogel synthesis represents both an environmental imperative and an opportunity for cost reduction. Natural polysaccharides, such as chitosan and alginate, offer hydrophilicity, biodegradability, and chemically modifiable functional groups, providing a materials-level rationale for their consideration as bio-based components. Concurrent development of biodegradation pathways and recycling strategies will be essential for clinical adoption and environmental responsibility [31,50,71,168,256,381,382,383,384,385,386,387].

### 5.3. Conclusions

Conductive PVA-based hydrogels offer tunable networks for wearable sensing. This review connects fabrication strategies and electronic, ionic, and hybrid conduction pathways with sensing applications and practical operating constraints. Advances in crosslinking and composite design have supported demonstrations of motion sensing, electrophysiological recording, and biochemical monitoring. Collectively, these studies highlight the importance of considering charge transport, mechanical compliance, and environmental stability together, rather than optimizing individual properties in isolation.

A central conclusion is that material selection should be guided by the intended application and operating environment. Conductivity enhancement must be balanced against network uniformity, mechanical recovery, hydration stability, and interface reliability. High conductivity or tensile strength alone is insufficient to establish sustained wearable performance. Accordingly, formulation and processing choices should be assessed at the assembled-device level, where deformation, environmental exposure, and electrical coupling jointly influence signal stability.

Further progress requires reproducible fabrication, comparable testing protocols, and validation under conditions representative of actual use. Priorities include controlling processing-dependent variability, maintaining hydration and stable interfaces during repeated operation, and evaluating the safety and performance of complete devices over relevant use periods. By linking material design to these requirements, conductive PVA-based hydrogels can be advanced toward more reliable and application-specific wearable health-monitoring systems.

## Figures and Tables

**Figure 1:**
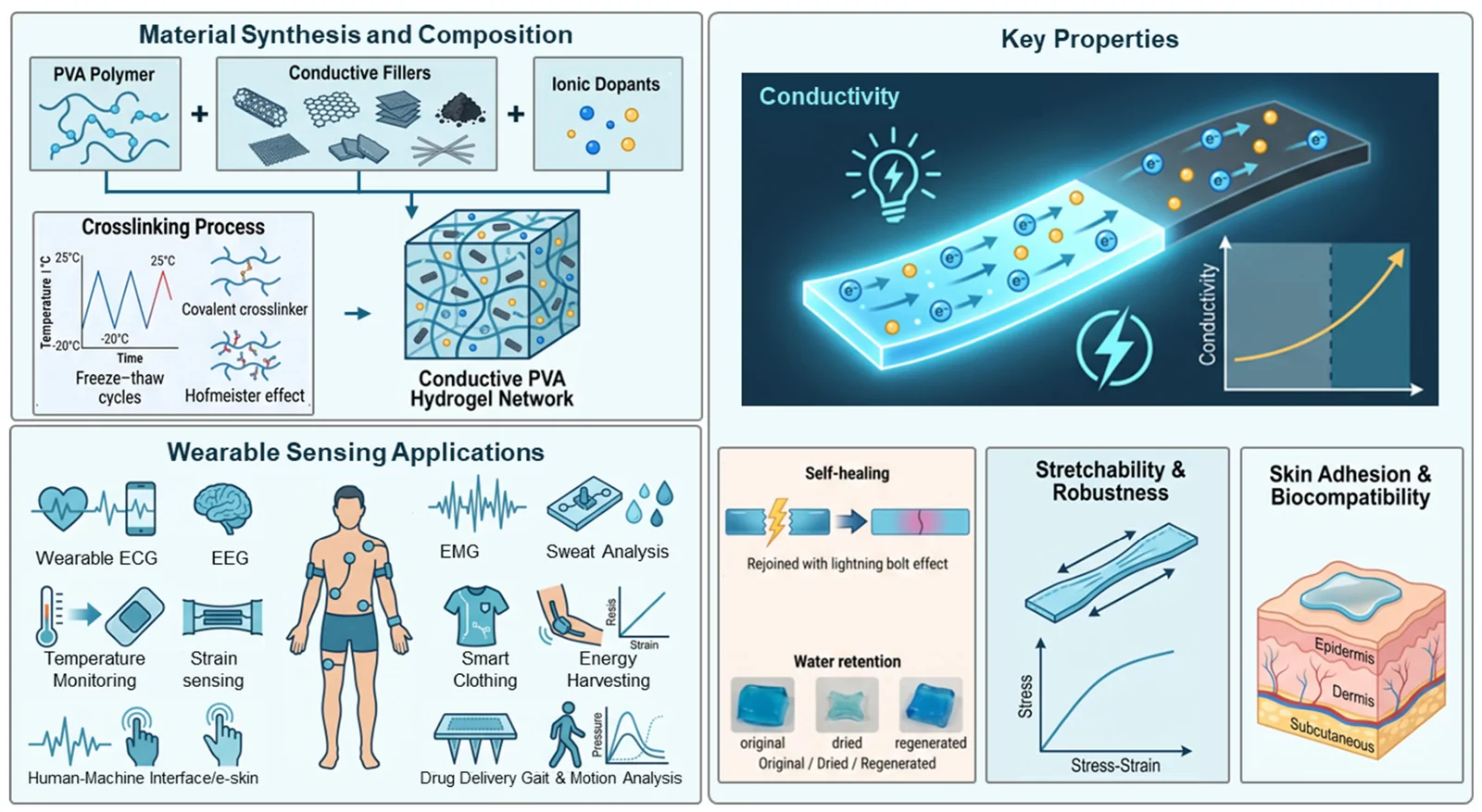
Overview of conductive PVA-based hydrogels for wearable electronic applications: Material synthesis and composition; Key properties; and Wearable sensing applications. Figure 1 schematic was initially generated using Nano Banana 2 (AI design tool) and subsequently refined by manual element extraction and reorganization in Microsoft PowerPoint.

**Figure 2:**
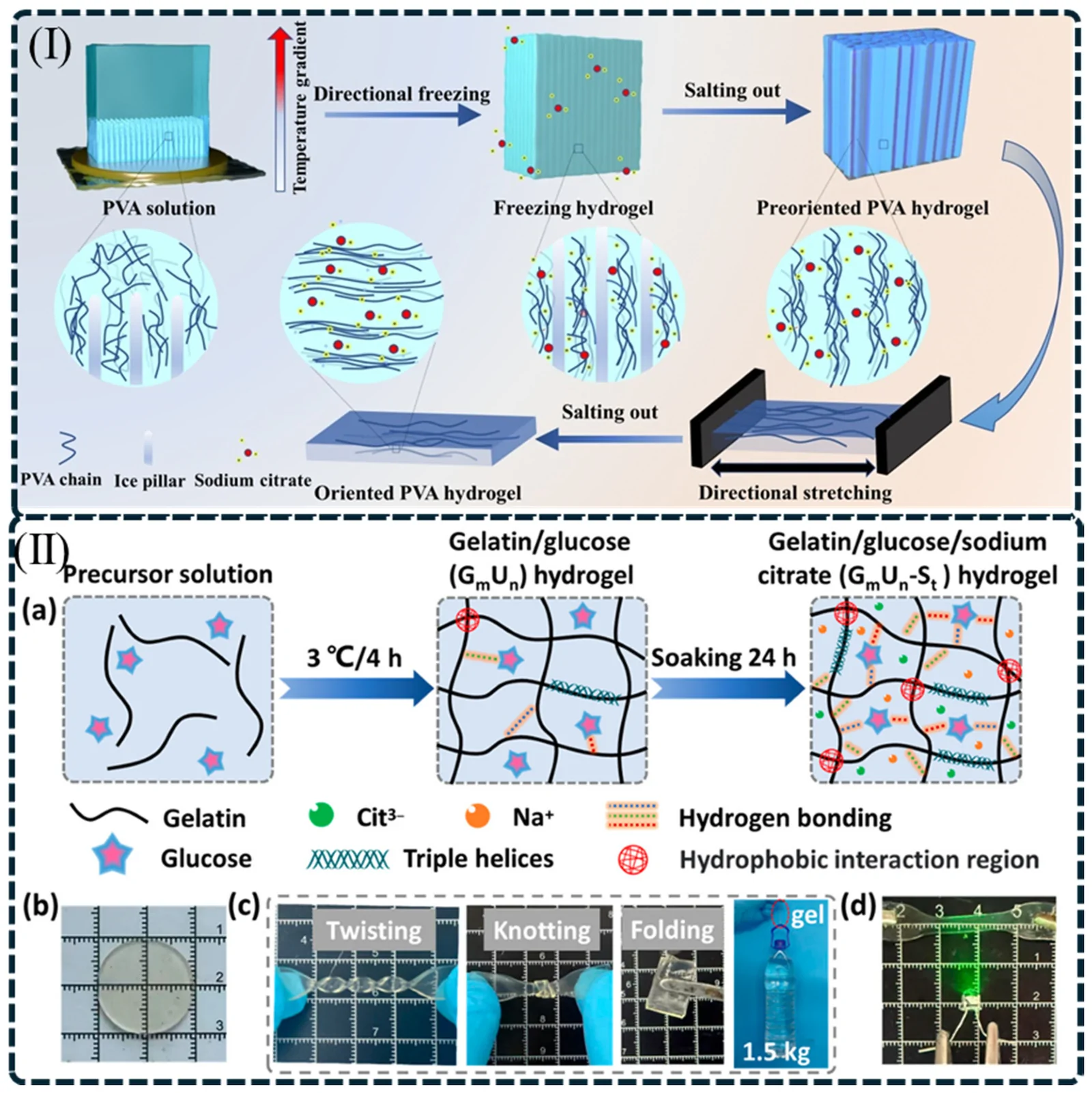
(**I**)—Schematic diagram for the preparation of oriented, orderly PVA hydrogels with an anisotropic structure. PVA: Polyvinyl alcohol. Reproduced from [66], available under terms of the CC BY 4.0 license, Copyright 2025, Zhang J.L, et al. (**II**)—(**a**) Schematic illustration of the preparation process of GmUn-St hydrogel. (**b**) Picture of the G220U300-S20 hydrogel. (**c**) Demonstration of the mechanical flexibility of G220U300-S20 hydrogel under various deformations, including twisting, knotting, folding, and lifting a 1.5 kg bottle. (**d**) Photograph showing the G220U300-S20 hydrogel powering a LED. Reproduced from [33], available under terms of the CC BY 4.0 license, Copyright 2026, Sang W. et al.

**Figure 3:**
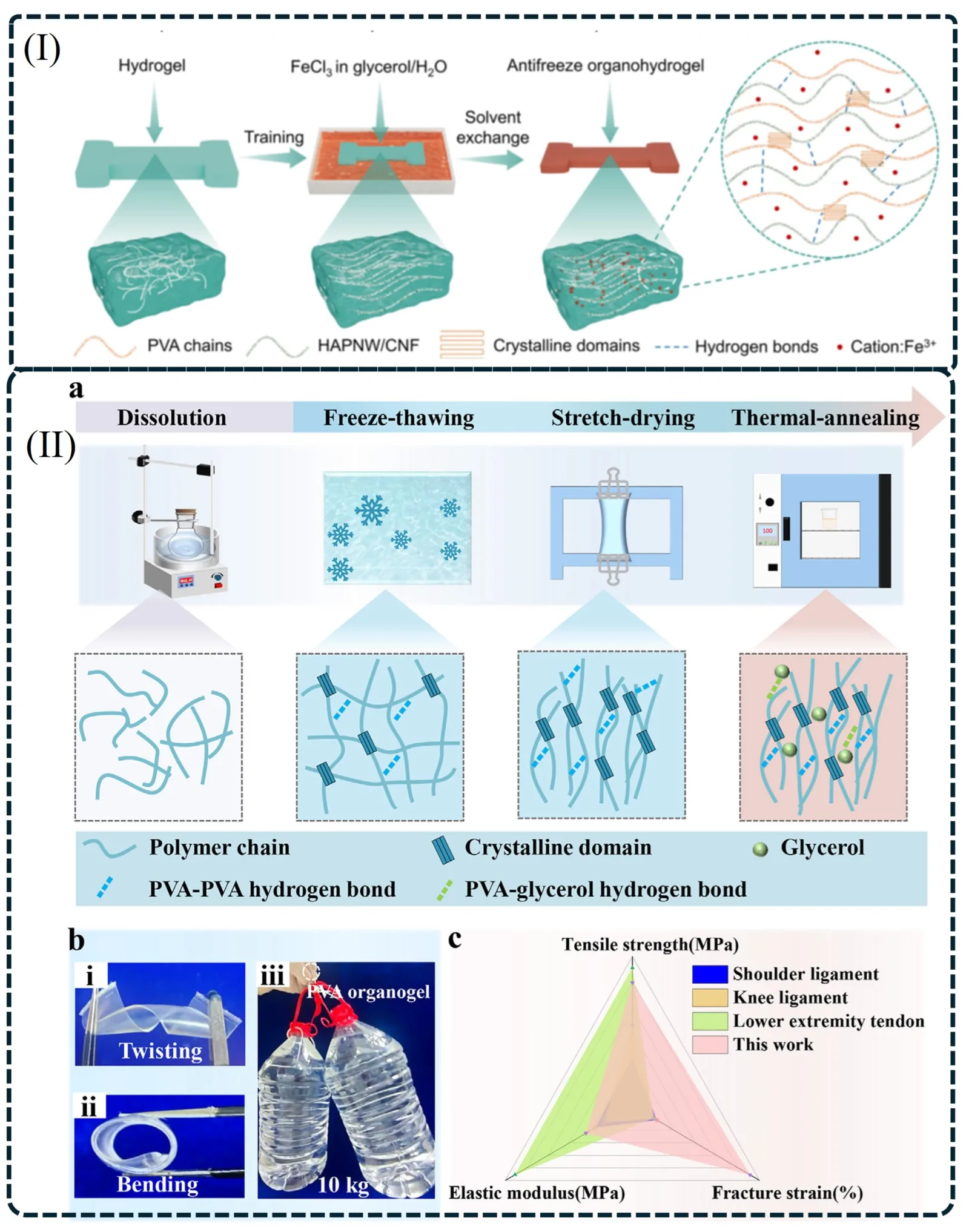
(**I**)—Illustration of the solvent exchange-assisted mechanical training process. Reprinted with permission from [124]. (**II**)—Schematic illustration of the preparation process and properties of anisotropic organogel. (**a**) Preparation of anisotropic organogel through freeze-thawing, stretch-drying, and thermal annealing strategy. (**b**) Demonstration of the organogel’s (**i**,**ii**) anti-deformation capability and (**iii**) mechanical strength, which lift a weight of 10 kg. (**c**) Comparison of mechanical properties of the developed organogel relative to various natural biological tissues. Reprinted with permission from [36].

**Figure 5:**
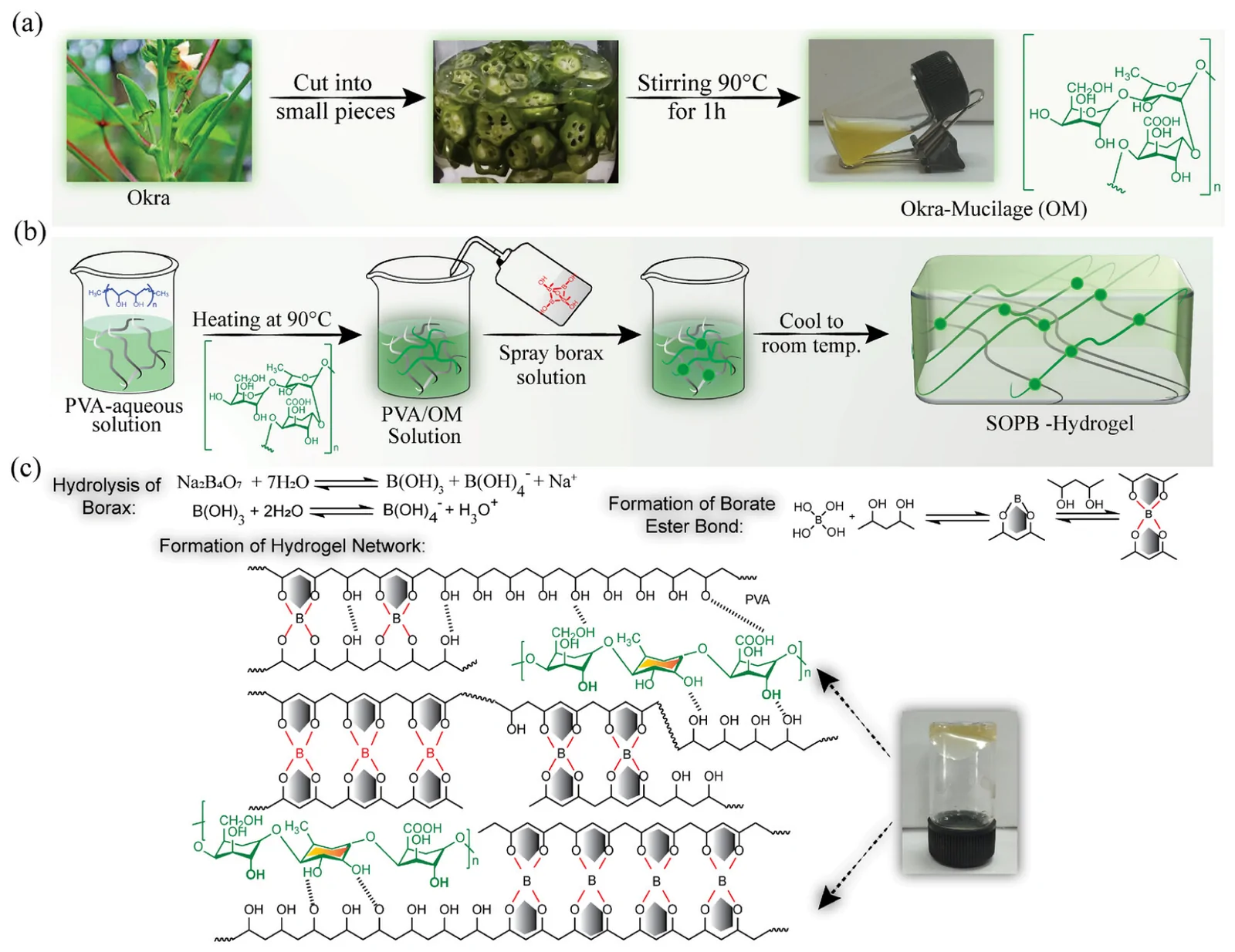
Schematic representation illustrates the preparation of (**a**) okra mucilage, (**b**) the structural formation of the SO1P5B hydrogel, and (**c**) the proposed chemical reactions occurring during SO1P5B hydrogel synthesis. Reprinted with permission from [7].

**Figure 6:**
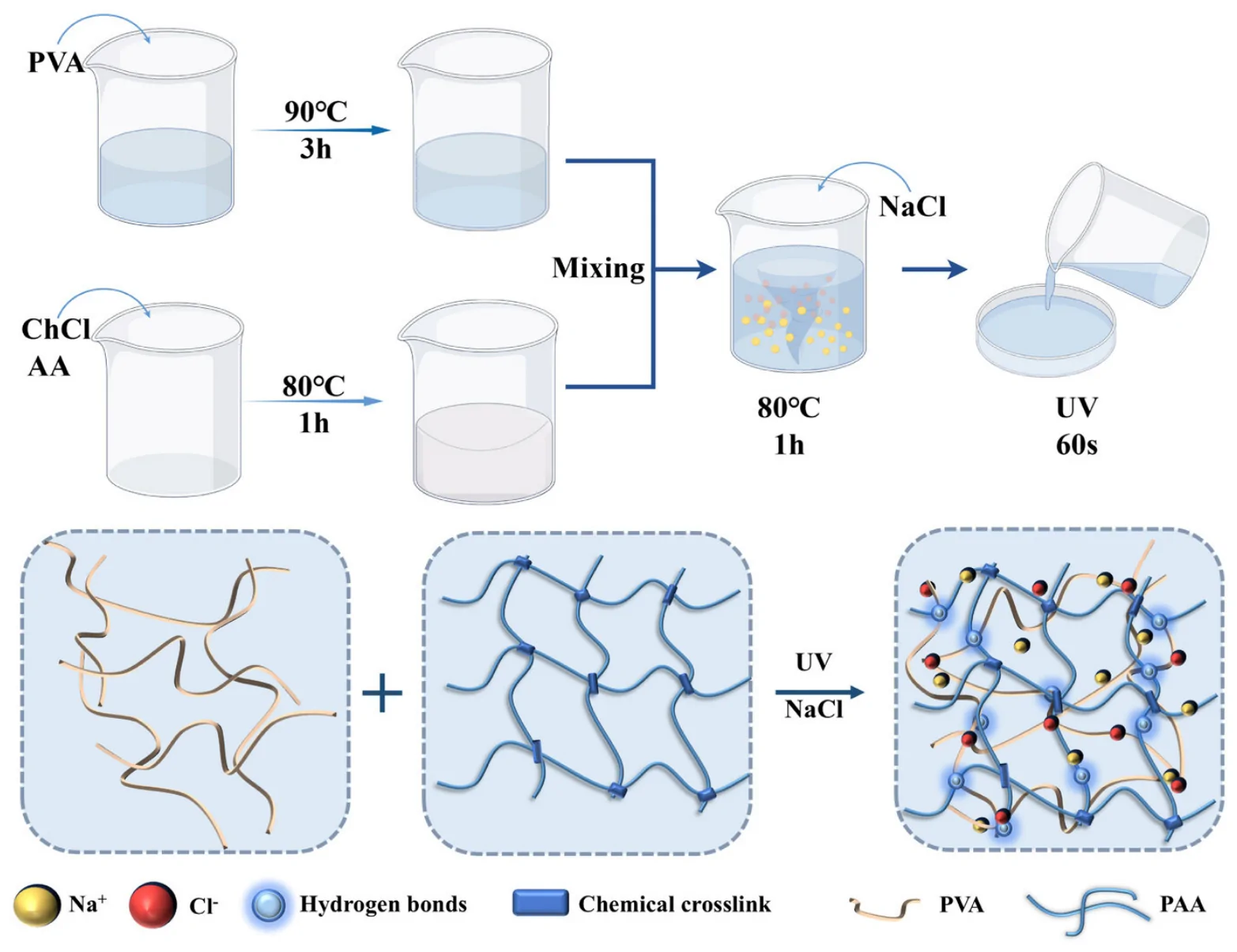
Synthesis process and schematic of the network structure of PVA/DES/NaCl elastomers (By Figdraw). Reproduced from [52], available under terms of the CC BY 4.0 license, Copyright 2025, Wang P.W. et al.

**Figure 7:**
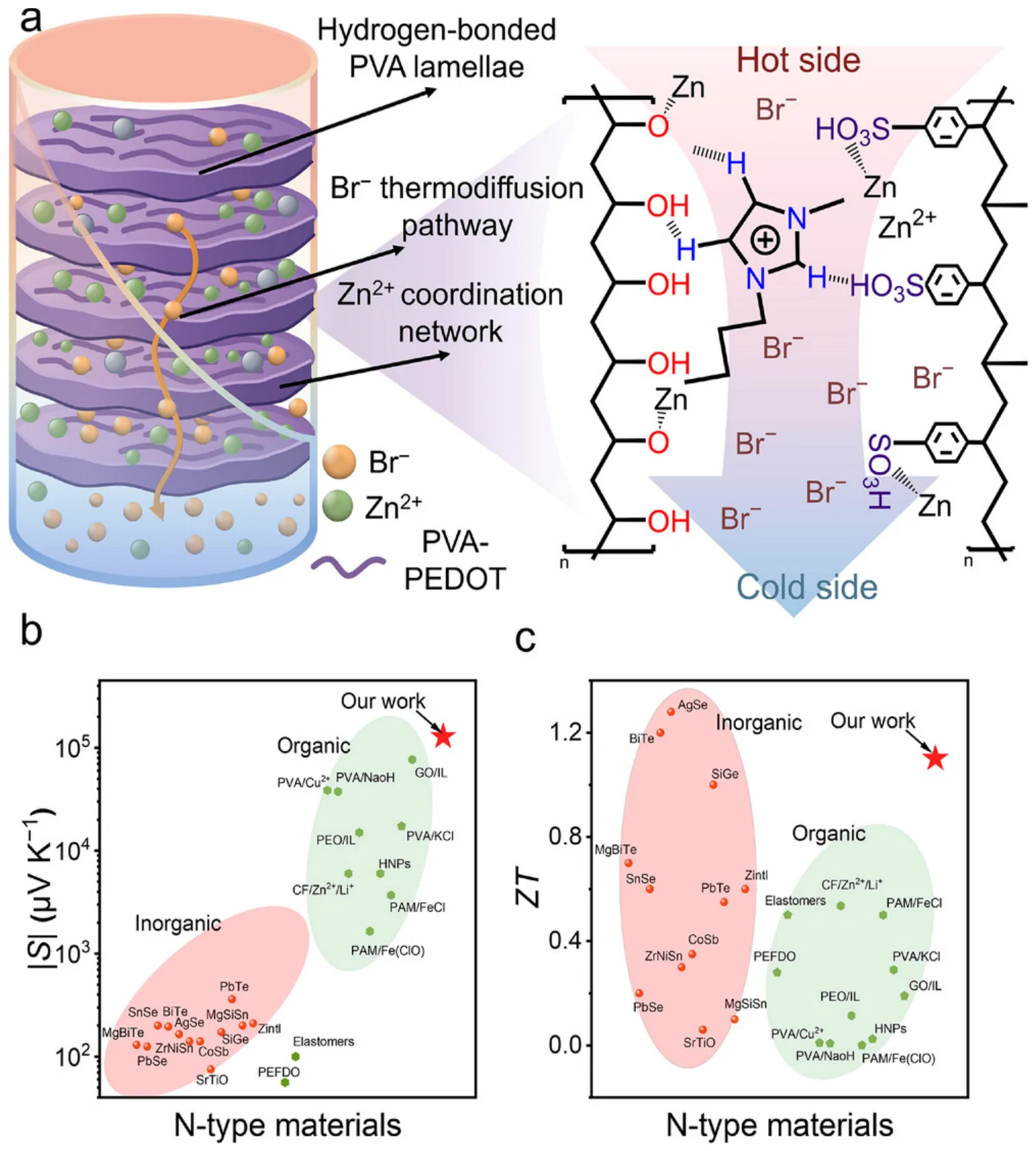
Structural architecture, thermodiffusion mechanism, and room-temperature thermoelectric benchmarking of the [BMIM]_5_[ZnBr]_1_ & PVA–PEDOT ionogel. Reproduced from [197], available under terms of the CC BY-NC-ND 4.0 license, Copyright 2026, Zhang C.Y. et al.

**Figure 8:**
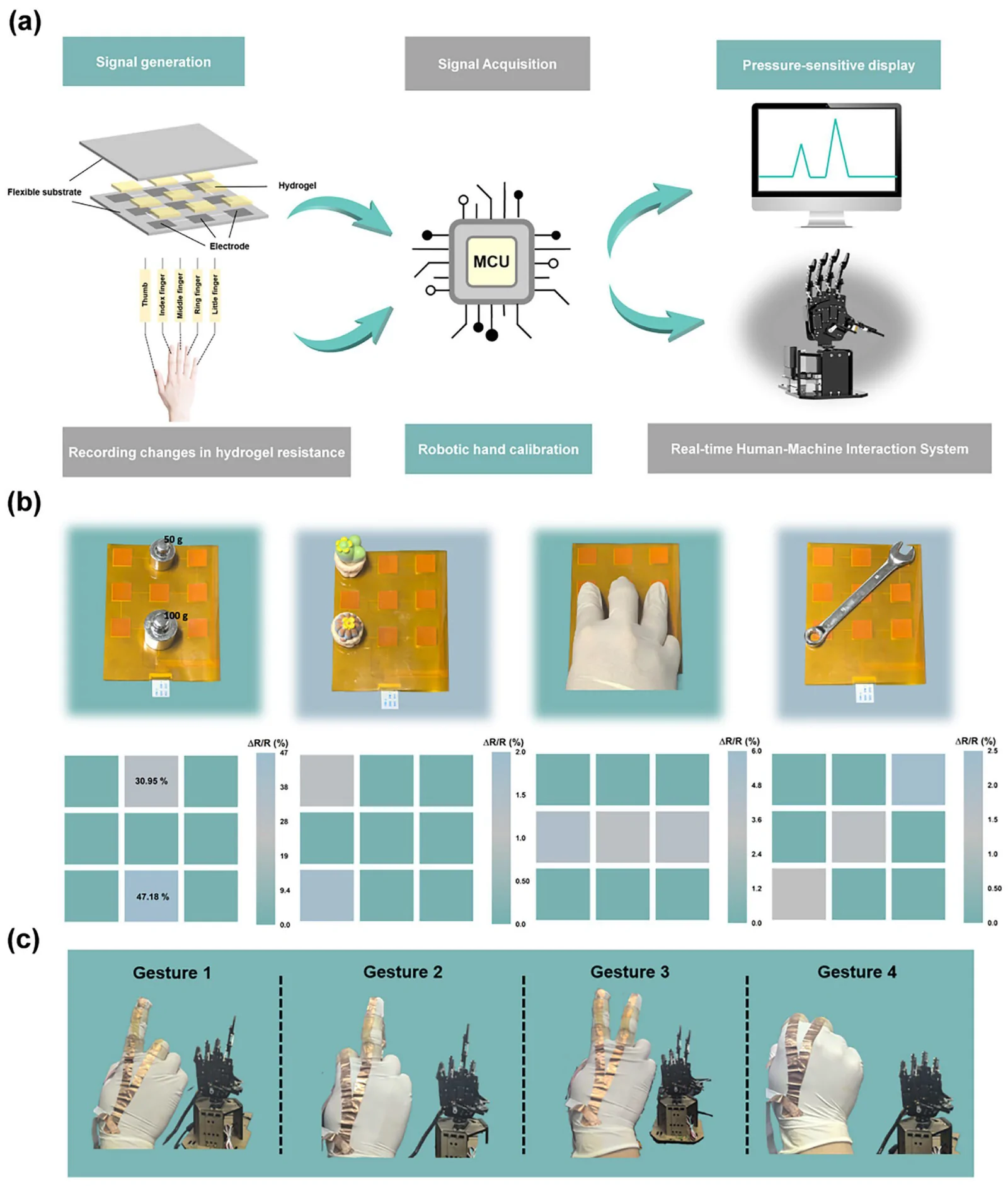
(**a**) Schematic diagram of the 3 × 3 hydrogel sensor array and robotic hand driving system. (**b**) Optical photographs and corresponding response thermal maps of the 3 × 3 sensor array pressed by different objects. (**c**) Photographs showing the robotic hand mimicking various human hand gestures. Reprinted with permission from [257].

**Figure 9:**
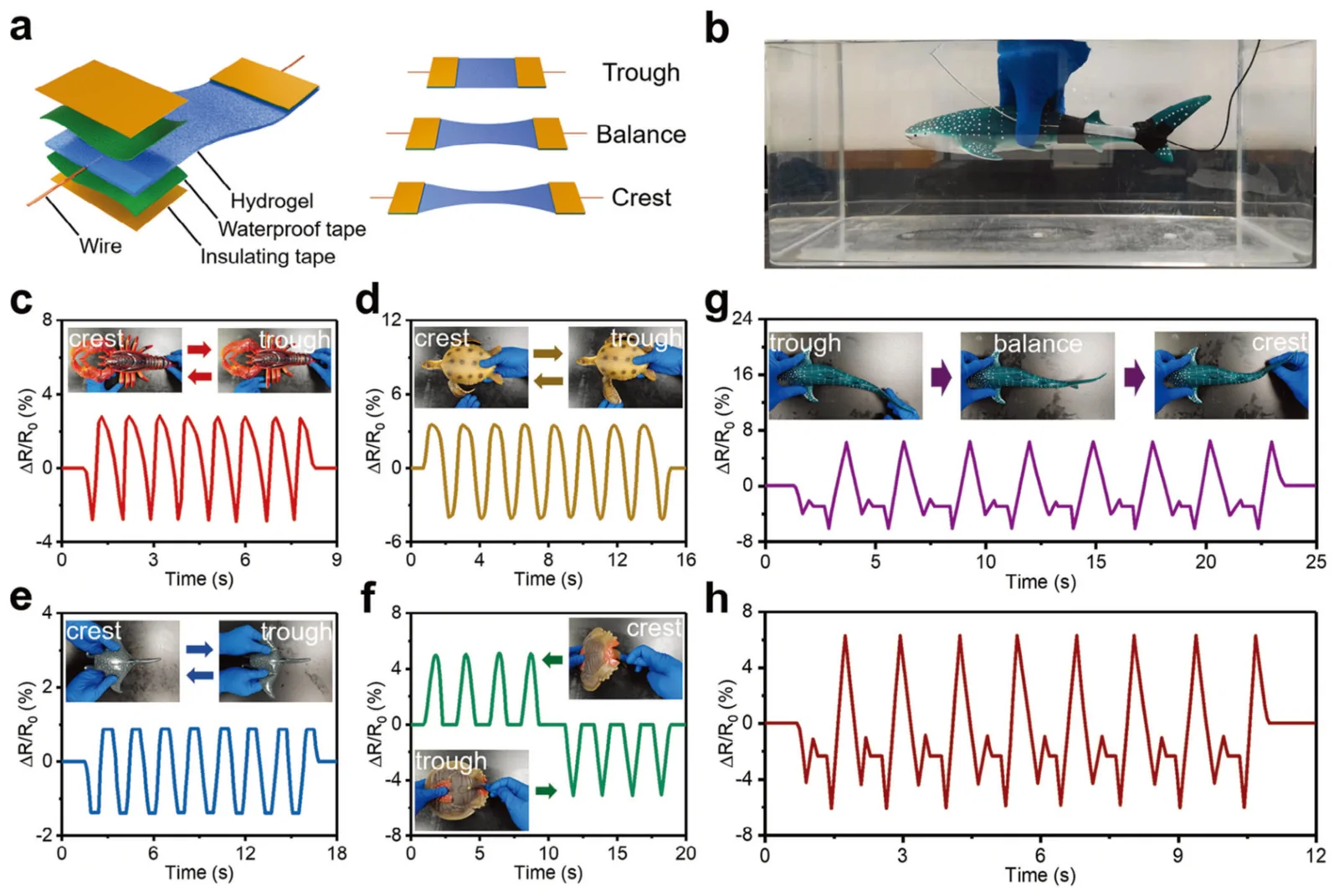
Underwater motion monitoring of marine animal models. (**a**) Schematic illustration of PVA/PSBMA-H_2_SO_4_ hydrogel strain sensor. (**b**) Image of an underwater strain sensor assembled on a shark model. Real-time relative resistance changes in hydrogel sensors for motion monitoring of marine animal models, including (**c**) crawling of the lobster, (**d**) gliding of the sea turtle, (**e**) swimming of the devil ray, (**f**) flipping of the horseshoe crab, and (**g**) swimming of the shark. (**h**) Real-time relative resistance changes of the hydrogel sensor for a swing of a shark model at high speed. Reprinted with permission from [41].

**Figure 10:**
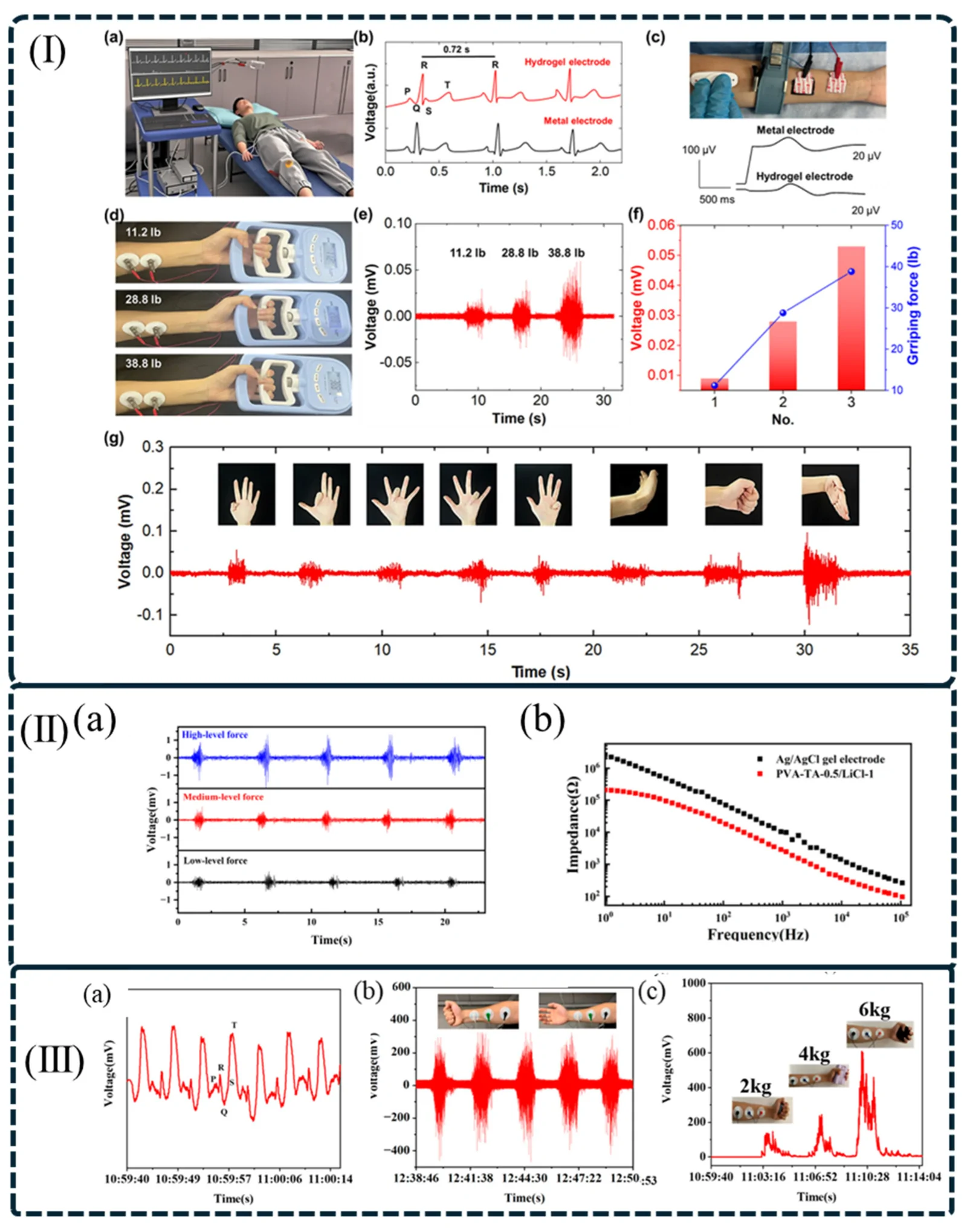
(**I**) ECG and EMG measurements using dm-(PEDOT:PSSnano/PVA)9/1-Na_2_SO_4_ hydrogel electrodes. Reprinted with permission from [278]. (**II**) Application of PVA-TA-0.5/LiCl-1 hydrogel. (**II**-**a**) EMG signals when holding grip at different levels of force. (**II**-**b**) Impedance spectra of commercial Ag/AgCl gel electrode and PVA-TA-0.5/LiCl-1 skin interface. Reproduced from [279], available under terms of the CC BY 4.0 license, Copyright 2025, Pan X.Y. et al. (**III**) Human physiological signal detection of the PVA/AA-SBMA hydrogel: (**III**-**a**) ECG signals, (**III**-**b**) EMG signals of arm muscle tension, and (**III**-**c**) EMG signals under different strengths. Reproduced from [280], available under terms of the CC BY 4.0 license, Copyright 2025, Deng Z.X. et al.

**Figure 11:**
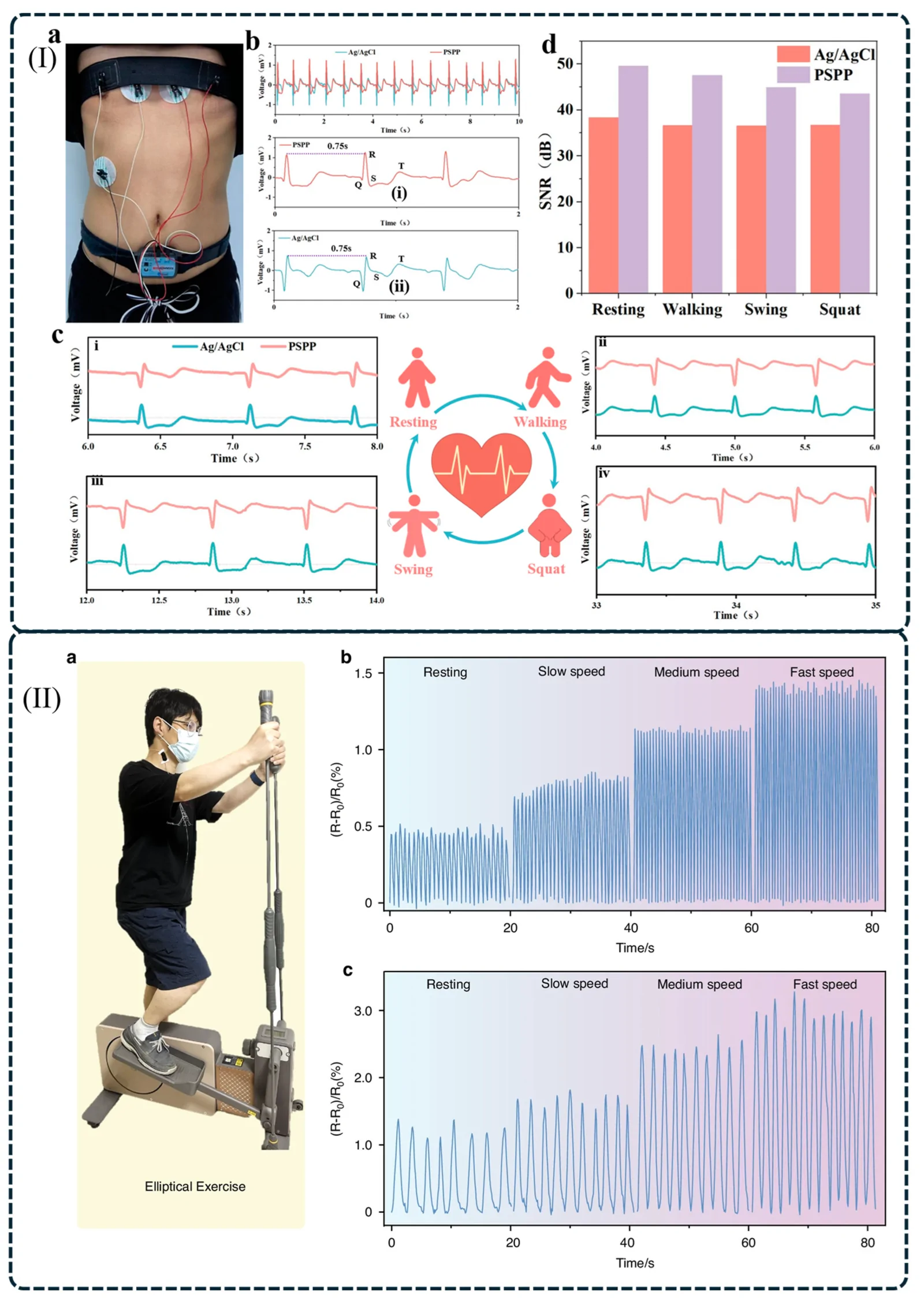
(**I**)—(**a**) Optical image of PSPP hydrogel paired with elastic bandage and disposable Ag/AgCl electrode patch placement. (**b**) Comparison of ECG waveforms between PSPP hydrogel and disposable Ag/AgCl electrodes. (**i**) R-R interpeak of PSPP hydrogel. (**ii**) R-R interpeak of commercial Ag/AgCl gel electrode (**c**) SNR under resting, walking, arm swinging, and squatting states: (**i**) ECG waveform in the resting state, (**ii**) ECG waveform in the walking state, (**iii**) ECG waveform in the arm swinging state, (**iv**) and ECG waveform in the squatting state. (**d**) Signal-to-noise ratio of hydrogel and disposable Ag/AgCl electrodes under four different states. Reproduced from [293], available under terms of the CC BY 4.0 license, Copyright 2025, Zheng J.J. et al. (**II**)—(**a**) Illustration of PBTM-3 hydrogel sensor on carotid artery during elliptical exercise. PBTM-3 hydrogel sensor for the right carotid artery (**b**), respiration (**c**) for monitoring the sensing performance. Reproduced from [295], available under terms of the CC BY-NC-ND 4.0 license, Copyright 2025, Wang X.Y. et al.

**Figure 12:**
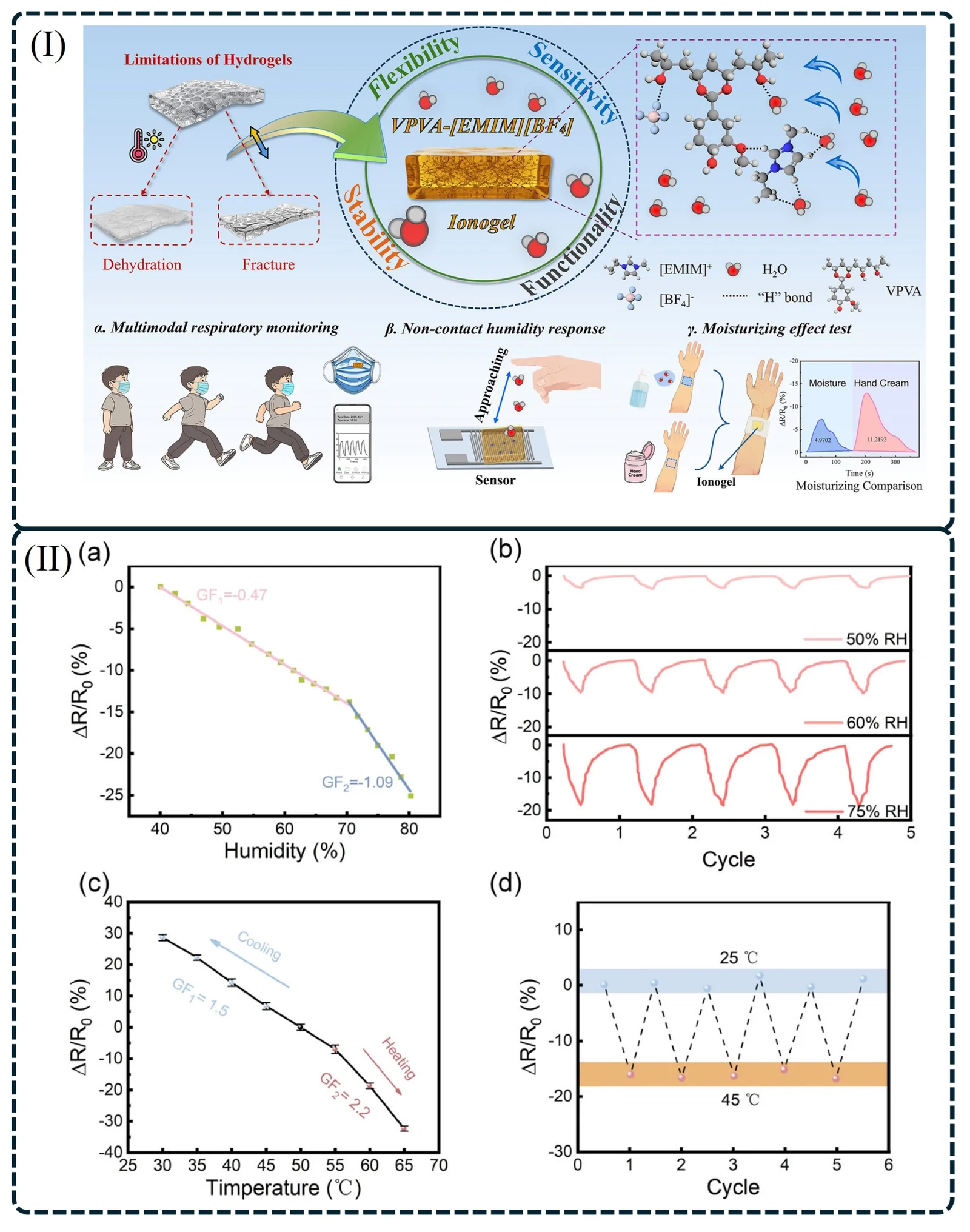
(**I**)—Schematic diagram illustrating the key features, structure, and multiple applications of the VPVA-[EMIM][BF_4_] ionogel flexible humidity sensor: (**α**) a mask-integrated system for multimodal respiratory monitoring, (**β**) non-contact monitoring of skin humidity, (**γ**) evaluation of the moisture retention capacity of moisturizing products. Reprinted with permission from [299]. (**II**)—(**a**) Humidity sensitivity response curve of the PVA/PAM/LiCl/MXene hydrogel sensor. (**b**) Stability of the hydrogel’s relative resistance change at different humidity levels. (**c**) ∆R/R_0_ curve of hydrogel real-time response to temperature. (**d**) Excellent stability and repeatability after several cycles of hot and cold temperature variations. Reproduced from [300], available under terms of the CC BY 4.0 license, Copyright 2025, Li R.R. et al.

**Figure 13:**
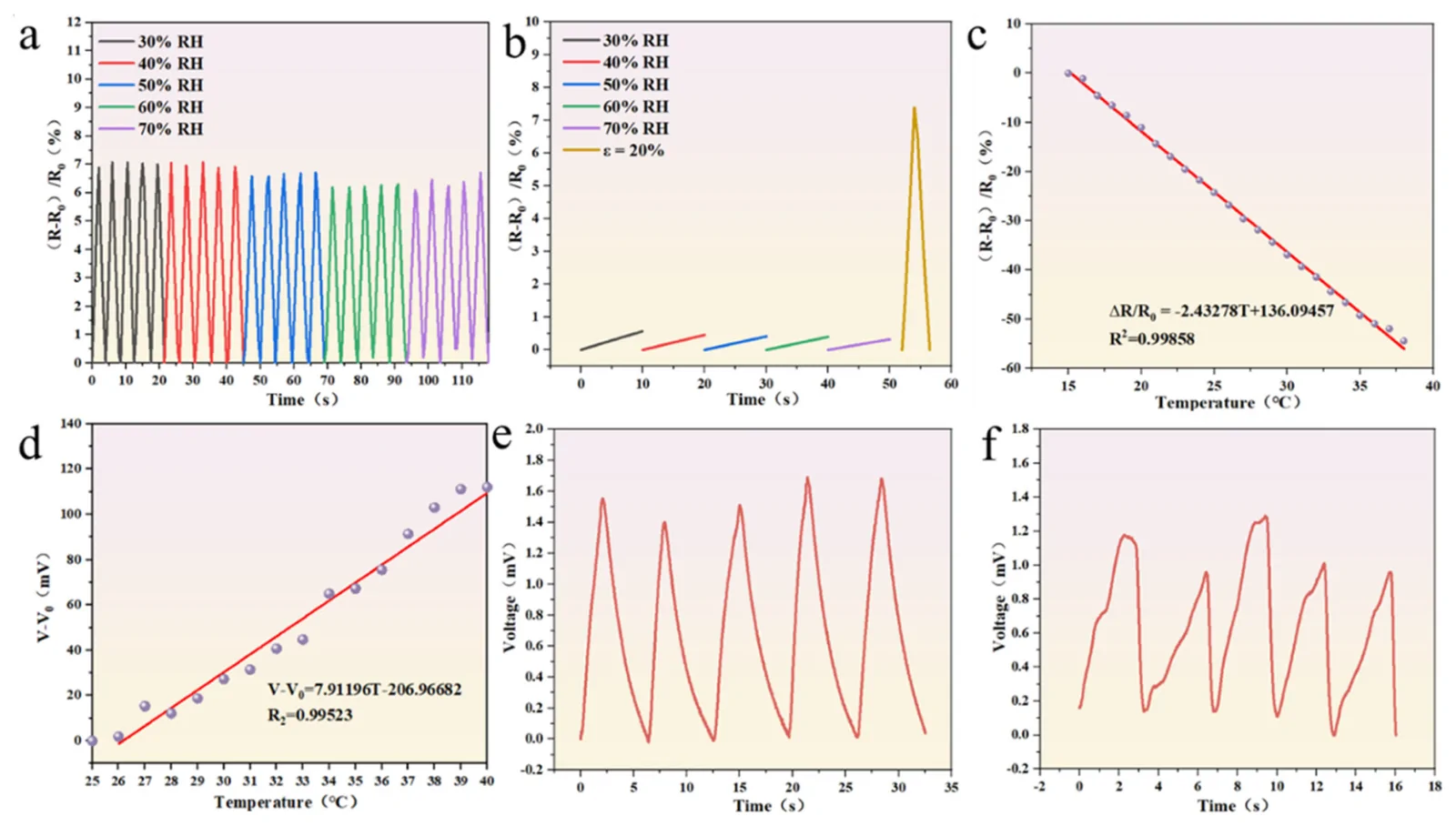
Temperature sensing characteristics of PSPSH (**a**) resistance change at different humidity and 20% stretch (**b**) resistance change at different humidity in static condition (**c**) resistance–temperature correlation curves (**d**) voltage–temperature correlation curves (**e**) voltage change at the touch of a finger (**f**) voltage change in breathing condition. Reproduced from [308], available under terms of the CC BY-NC 3.0 license, Copyright 2026, Zhou J.L. et al.

**Figure 14:**
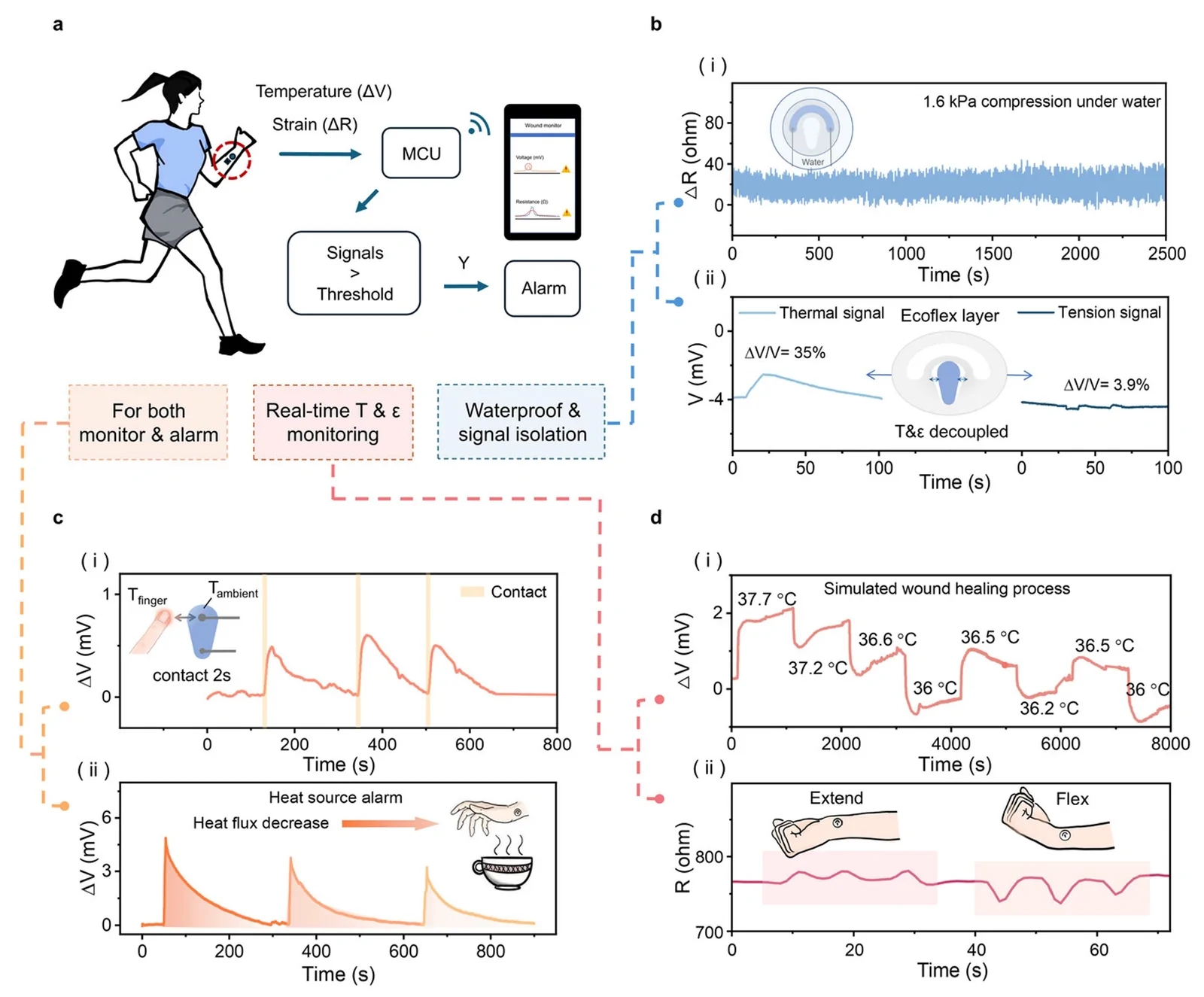
Integrated applications of the wound monitoring patch. (**a**) Schematic of the wound monitoring system to realize remote control. (**b**) (**i**) Impedance stability under 1.6 kPa compression in water. (**ii**) Decoupling of thermal and mechanical signals in the wound monitoring patch, where ΔV/V ~35% for thermal stimuli and ~3.9% for strain. (**c**) (**i**) Detection of finger temperature against ambient temperature with a contact time of 2 s. (**ii**) Dynamic thermal test during cooldown of a hot source for a heat alarm. (**d**) (**i**) Continuous monitoring of a simulated wound-healing process with temperature changes. (**ii**) Impedance response to wrist extension and flexion. Reproduced from [8], available under terms of the CC BY-NC-ND 4.0 license, Copyright 2026, Fang Y. et al.

**Figure 15:**
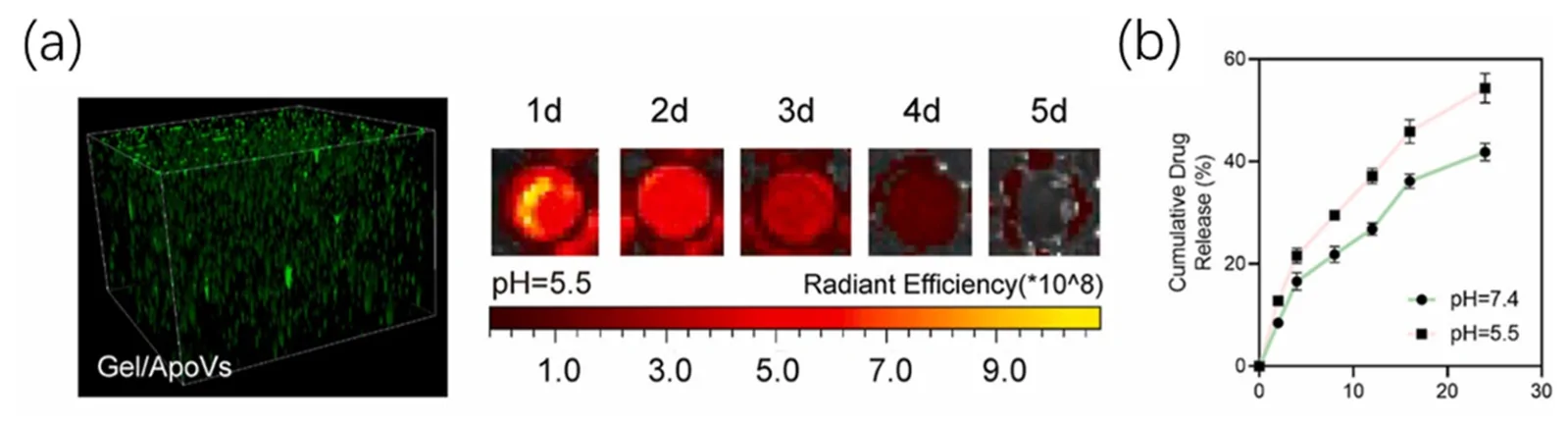
Preparation and characterization of ApoVs. (**a**) Confocal images of DiO-ApoVs in composite hydrogels and in vitro release of ApoVs from the composite hydrogels in PBS. (**b**) Cumulative release of ApoVs from the hydrogel in response to the acidity of the microenvironment. Reproduced from [50], available under terms of the CC BY-NC-ND 4.0 license, Copyright 2026, Yan Z.N. et al.

**Figure 16:**
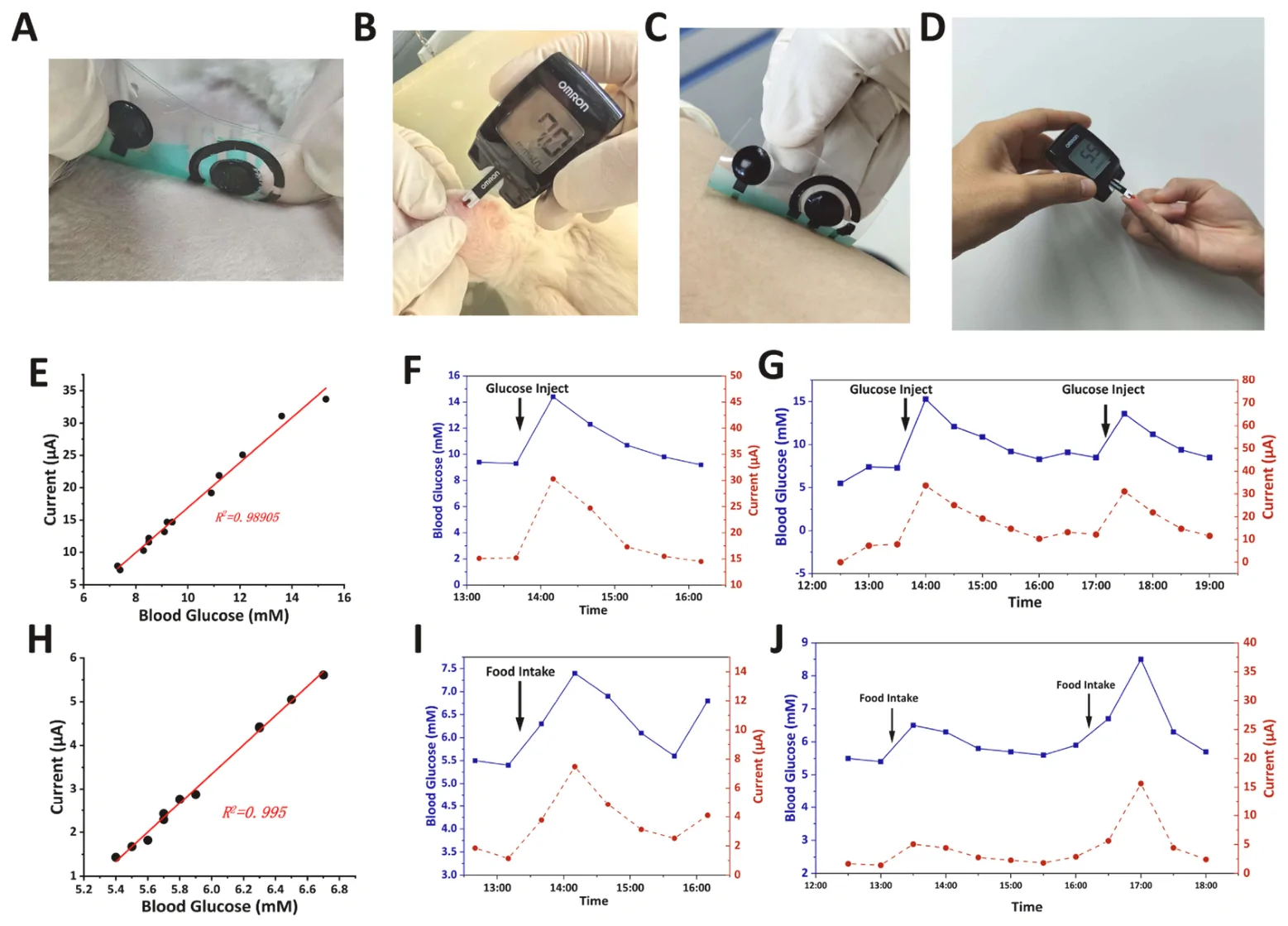
The blood glucose examination of GM3PAS on the skin surface. Reproduced from [338], available under terms of the CC BY-NC-ND 4.0 license, Copyright 2026, Li Z.X. et al.

**Figure 17:**
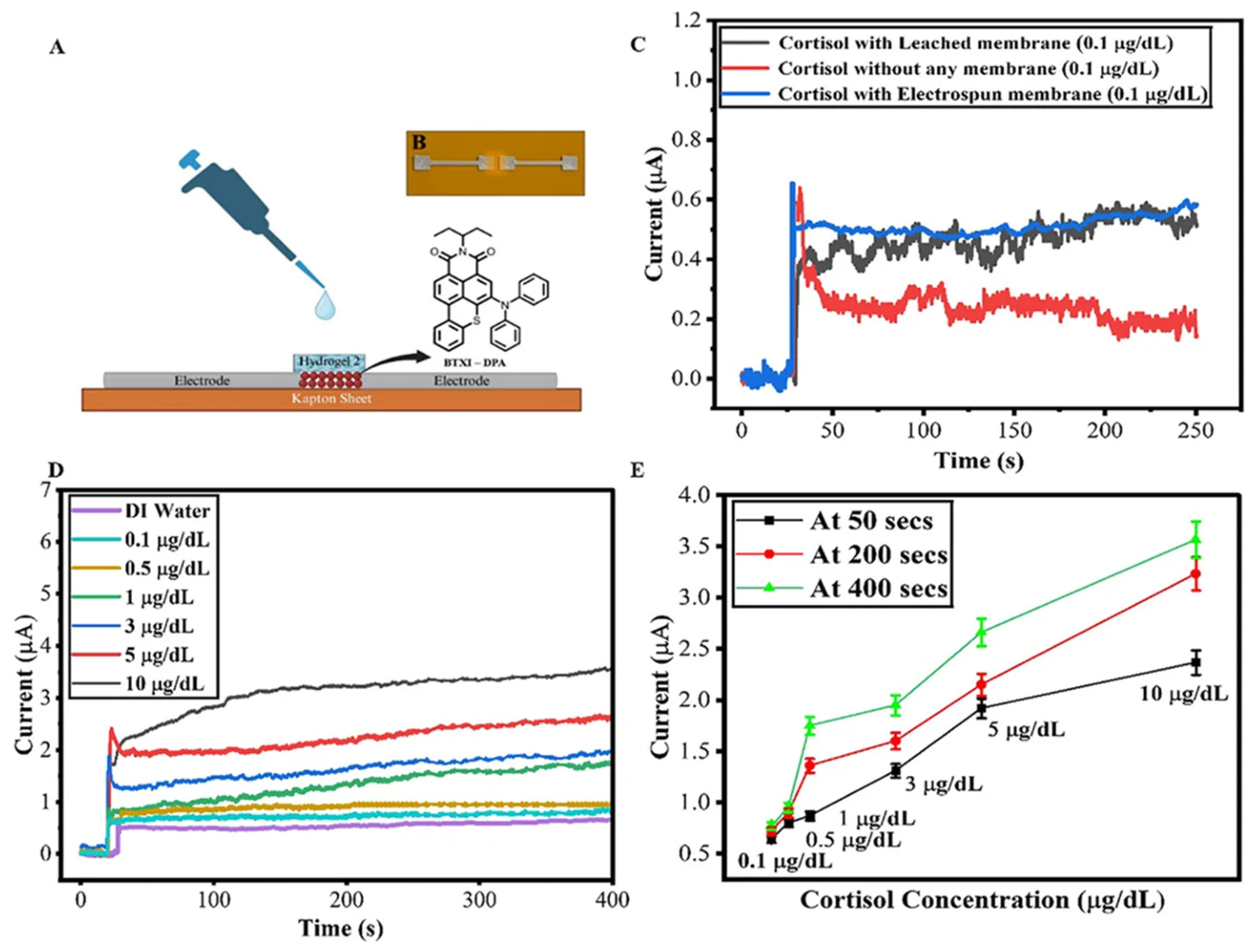
Biosensing performance of porous hydrogels. Reproduced from [345], available under terms of the CC BY 4.0 license, Copyright 2026, Pandit, Parth, et al.

**Table 1:** Comprehensive crosslinking strategy comparison of PVA-based hydrogels.

Crosslinking Strategy	Crosslinking Mechanism	Quantitative Performance Indicators	Key Advantages	Critical Limitations	Main Wearable Applications	Ref.
Physical Crosslinking	1. Hydrogen bonding 2. PVA crystallites 3. chain entanglement 4. does not control crosslink density, prone to non-uniform networks	1. Young’s modulus: <10 kPa 2. Tensile strength: <0.5 MPa 3. Cyclic durability: <500 cycles 4. Water retention: excellent	1. Environmentally benign, non-toxic 2. High biocompatibility 3. Recyclable and recastable	1. Limited mechanical strength 2. Weak fatigue resistance under cyclic deformation 3. Temperature & humidity sensitivity 4. Environmental degradation (sweat, physiological fluids)	1. Transient and short-term wearable sensors 2. Flexible, low-stress applications	[63,64,65,66,67,68,69]
Chemical Crosslinking	1. Covalent bonding via crosslinking agents forms irreversible network structure	1. Young’s modulus: 50–150 kPa 2. Tensile strength: 0.5–1.41 MPa 3. Fatigue resistance: >1000 cycles 4. Low residual strain: <5% after repeated cycling 5. High transparency: ≥88%	1. Superior mechanical robustness and durability 2. Excellent long-term network stability 3. Customizable crosslink density 4. Reproducible performance	1. Residual toxicity from chemical crosslinkers (biocompatibility concern for skin contact) 2. Poor recyclability 3. Challenging process control 4. Current research shifting toward green biocompatible alternatives	1. Long-term skin-contact devices 2. Load-bearing applications 3. Posture & motion monitoring 4. High-performance wearables	[38,62,70,71,72,73]
Radiation Crosslinking	1. Free-radical-induced covalent crosslinks via γ-rays or electron beams 2. formed by radical recombination after polymer chain bond scission	1. Uniform crosslink density across bulk material 2. Minimal residual radiation 3. High purity (no chemical additives) 4. Tunable network architecture	1. Chemical-free formulation, exceptionally clean 2. Excellent for biomedical applications 3. Precise control of crosslink density 4. Deep penetration (γ-rays for bulk samples)	1. High capital investment (>US$1M facility) 2. Risk of polymer backbone degradation 3. Limited scalability for small-scale production 4. Specialized infrastructure required	1. Biomedical-grade hydrogels; high-purity wearable devices 2. Implantable bioelectronics	[74,75,76,77,78]
UV Crosslinking	1. Photo-initiated free-radical polymerization (254–365 nm activation wavelength); 2. occurs at room temperature and atmospheric pressure	1. Reaction kinetics: 1–10 min 2. Spatial/temporal resolution: micron-scale 3. Penetration depth: <1 mm	1. Rapid reaction kinetics (minutes) 2. Excellent spatial and temporal control 3. Precise micro-patterning capability 4. Room-temperature operation	1. Photoinitiator toxicity (residual monomers in cured structure) 2. Shallow penetration depth (<1 mm) 3. Risk of polymer backbone scission 4. Incomplete surface crosslinking in thick samples—Biocompatibility concerns	1. Micropatterned flexible sensors 2. Micro-scale wearable devices 3. Inkjet-printed electronics	[79,80]
Hybrid/Dynamic Crosslinking	1. Combined covalent + non-covalent bonds (ionic coordination, boronic ester dynamic bonds, hydrogen bonding); 2. Synergistic physical and chemical interactions	1. Tensile strength: 0.3–1.54 MPa 2. Young’s modulus: ~92 kPa 3. Stretchability: up to 858% 4. Ionic conductivity: 0.29 S/m 5. Energy at break: 5.14 MJ/m^−3^ 6. Exceptional fatigue resistance: >20 cycles	1. Balanced strength and toughness 2. Self-healing capability (reversible networks) 3. Adaptive mechanical properties 4. Multifunctional performance 5. Superior fatigue resistance	1. Increased system complexity 2. Batch-to-batch reproducibility challenges 3. Higher processing costs 4. Mechanistic interactions remain inadequately understood	1. High-performance wearables for physiological monitoring 2. Electronic skin (e-skin) with adaptive properties 3. Self-healing multifunctional devices	[73,81,82]

**Table 2:** Comparison of conductivity-enhancement strategies for PVA-based hydrogels.

Strategy	Representative Additives	Dominant Charge-Transport Mechanism	Main Advantages	Key Limitations	Ref.
Carbon-based nanofillers	CNTs (incl. carboxylated CNTs), graphene, GO/GO-COOH, carbon black	Electronic transport through percolated filler networks; tunneling and contact conduction between adjacent fillers	Processing flexibility; tunable conductivity and mechanical reinforcement; compatible with anisotropic structuring (e.g., ice templating)	Filler agglomeration and inhomogeneous dispersion; junction/contact resistance; conductivity of GO depends on its oxidation/reduction state	[174,175,176,177,178,179]
Metallic nanostructures	Ag nanowires, Ag nanoparticles, liquid metals	Direct electronic conduction through intrinsically conductive metallic phases	High intrinsic conductivity; compatible with self-healing designs; good sensitivity and fatigue resistance	High material cost; potential Ag ion cytotoxicity and biocompatibility concerns; oxidation in humid environments; limited long-term stability data	[180,181,182]
Conducting polymers	PPy, PANI, PEDOT:PSS, PPV	Transport of doping-generated charge carriers (polarons/bipolarons) along conjugated backbones and between chains or PEDOT-rich domains; PVA mainly stabilizes the composite structure	Tunable conductivity; homogeneous, soft all-polymer networks; ease of synthesis	Brittleness under deformation; performance depends on doping state and connectivity of the conducting phase	[4,54,183,184,185,186,187,188]
Salt-based ionic conduction	Inorganic salts (e.g., NaCl, LiCl), acids/protons, zwitterions	Migration of mobile ions through the hydrated polymer network	Softness, stretchability, and transparency; mechanically robust formulations possible	Dehydration-induced conductivity loss; slower electrochemical response; hygroscopic behavior and limited long-term stability	[189,190,191,192,193,194,195]
Ionic liquids and deep eutectic solvents	Imidazolium-type and aprotic ILs; DESs	Ion migration in IL/DES-rich domains; ionic thermodiffusion in ionic thermoelectric designs	Anti-freezing behavior and improved environmental stability; multifunctional sensing; DESs are more eco-friendly and lower cost than ILs	Increased system complexity and reproducibility challenges; IL toxicity; higher processing cost than simple salts; dehydration only partly mitigated by DESs	[42,59,196,197,198,199,200,201,202,203]
Synergistic multi-strategy (hybrid) systems	IL + GO; conducting polymer + MXene; CNT + salt; MXene-based hybrids	Coupled ionic and electronic pathways; the dominant contribution depends on formulation and measurement conditions	Combines complementary conduction modes; improved conductivity–mechanics–durability balance	Material complexity; phase segregation and reproducibility issues; MXene oxidation and interfacial instability over time; multi-step processing costs	[51,203,204,205,206,207,208]

**Table 3:** Conductivity and selected performance parameters of representative PVA-based conductive systems discussed in Section 3.

Strategy	System (Sample State)	Conductivite (S/m)	Mechanical/Other Parameters	Ref.
Carbon	PVA/carboxylated CNT, ice-templated + salted-out (anisotropic hydrogel)	0.11 (along alignment axis)	Tensile strength 3 MPa; toughness 4 MJ·m^−3^; water content 65%	[175]
Carbon (GO)	PVA/PAA/agarose + GO-COOH, one-pot	10.56 ± 0.63	Fracture stress 122.3 kPa	[174]
Metal	PVA/borax + 0.2% M-AgNW	3.59 (before healing); 3.09 (after healing)	~86% healing efficiency, based on conductivity	[180]
Metal	PVA/NaNO_3_/Ag NPs	5.9	Tensile strength 0.35 MPa; TENG 120 V, 1.5 μA	[181]
Liquid metal	Encapsulated liquid-metal system	4.87	GF = 2.40	[182]
Ionic	s-PVA/PVA/NaCl hydrogel fiber	0.38	Fracture strength 20 MPa; toughness 67.2 MJ·m^−3^; GF = 1.9 (100–300% strain); 1200 cycles at 50% strain	[189]
Ionic (dual cation)	H^+^/M^+^-doped PVA, freeze–thaw	3.3 (=33 mS/cm)	Seebeck coefficient 7.43 mV·K^−1^	[195]
Hybrid ionic–electronic	Starch/PVA + ionic liquid + GO	1.98	Strength 0.64 MPa; GF = 6.04 (tension)/3.27 (compression); temperature sensitivity 0.71% °C^−1^; freeze resistance < −20 °C	[203]
Conducting polymer	PEDOT:PSS/PVP/PVA/TA semi-IPN (film)	1.3 × 10^5^ (=1300 S/cm)	266% strain; R/R_0_ = 1.06 after 3000 cycles at 100% strain	[187]

**Table 4:** Summary and comparison of quantitative performance for recent representative conductive PVA-based hydrogels used in human motion detection sensors.

Hydrogel System	Conductive Material	Tensile Strength (kPa)	Fracture Strain (%)	Sensing Range (%)	GF	Response Time	Highlight	Application	Ref.
PVA/Borax/PEDOT:PSS (VFSS)	PEDOT:PSS	500–3100	>500	N/A	1.0 (tensile) 0.994 (bending)	0.3 s	1. Ionic conductivity: 3.25 × 10^−2^ S/cm 2. Real-time activity monitoring; 3. Variable fluorescence sensing	1. Complex human activity detection; 2. Wearable electronics	[249]
PAM/PVA/TA@AL/Fe^3+^	TA@AL with Fe^3+^ post-impregnation	115 (optimal)	~900	0–550%	3.48–14.88	Response: 82 ms/Recovery: 71 ms	1. Coupled mechanical-electrical functionality; 2. Excellent tear resistance	1. Microscale motion sensing; 2. Facial expressions	[53]
PVA/SA/CNT (5 mg/mL) (PSC-2)	-CNTs; -mixed conduction (ionic + electronic); -3D interpenetrating network	Optimal	346	0–150	Higher sensitivity; optimal balance	0.26–0.28 s (at 4–8% strain); recovery time: 0.28–0.38 s	1. Total conductivity: 0.53 S/m (ionic + electronic); 2. Toughness: 55.83 kJ/m^3^; Strain sensing range: 0.4–150%; 3. Detection limit: 0.4%; Elastic recovery: excellent; 4. Anti-fatigue: >1000 cycles @ 30% strain; 5. Signal-to-noise ratio (SNR): 18.2 dB (vs. 7.5 dB for dry electrodes); 6. Stability: smooth curves at 50, 100, 200, 300 mm/min strain rates	1. Human motion detection; 2. ECG monitoring (rest, walk, run); 3. Wearable strain sensors; 4. High-precision bioelectronic devices	[44]
PVA/PAA/AG	PVA/PAA/Agarose + AlCl_3_ + Borax + GO-COOH	122.3	561	0–550%	3.66–11.31	Immediate response	1. Ionic conductivity: 10.56 ± 0.63 S/m Reversible brightness control demonstrating real-time conductivity changes	1. Multi-Modal Wearable Strain Sens	[174]
PANI/PVA/CMC-P(AM-co-AA)-Zn^2+^/EG (Optimal, EG:H_2_O = 1:3, 0.1 M Zn^2+^)	PANI + Zn^2+^ + CMC + P(AM-co-AA) + Ethylene Glycol	838	330	0–200%	2.94	Immediate resistance response to deformation;	1. Conductivity: 2.02 S/m; 2. Freezing tolerance: –41.6 °C while maintaining mechanical flexibility; 3. Moisture retention: 83.7% weight retention after 7 days at room temperature	1. Joint Motion Detection; 2. Human-Robot Interaction & Gesture Control; 3. Writing and Touch Recognition	[253]
DES/PVA/CS/TEOS_2_	PVA + Chitosan + DES + Silicic Acid (TEOS-derived)	220 ± 10	~303	0–400%	0.10–0.50	200 ms	1. Ionic conductivity: 38.1 mS/cm; 2. Freeze tolerance: 3. Anti-freezing; 4. Low-temperature activation energy; 5. Good biocompatibility	1. Physiological Monitoring; 2. Human Motion Detection 3. Advanced Morse Code Communication System	[257]
PGL (PVA/Gelatin/Lignin/Ionic Liquid [C_1_OC_2_mPy][NTf_2_])	Ionic liquid [C_1_OC_2_mPy][NTf_2_] + Lignin	868.16	512.7		2.91	301 ms	1. High energy dissipation; 2. Electro-repaired after breakage; 3. Specific capacitance: 104.8 F/g at 1 A/g; Working voltage: 1.2 V	1. Wearable Sensing; 2. Flexible Electronics	[256]
PVA/PSBMA/H_2_SO_4_	PSBMA (zwitterionic polymer) with H_2_SO_4_	~200	300	0–300%	2.1–11.31	<0.4 s	1. Ionic conductivity: 4.35 S/m; 2. Superior toughness (4.61 MJ/m^3^); 3. Real-time continuous monitoring with no baseline drift across 1000 cycles; 4. Non-Swelling Property	1. Underwater motion tracking; 2. Marine animal models (lobster crawling, sea turtle gliding, ray swimming, shark movements)	[41]
PVA-PDA@CNF-Ag	Polydopamine (PDA) + CNF + AgNPs	60	536	1–200	2.1	stable signal responses	1. Electrical Conductivity: 17–23 mS/m; 2. Strong, repeatable adhesion on diverse substrates; 3. Robust antibacterial activity; 4. Multi-Functional Integration	1. Human-Motion Wearable Electronics 2. Voice/Speech Monitoring	[263]

**Table 5:** Summary of recent representative PVA-based hydrogels applied in healthcare detection sensors.

PVA-Based Hydrogels	Conductive Components	Contact Impedance	Signal-to-Noise Ratio (SNR)	Commercial Comparison	Highlight	Application	Ref.
PVA/PAA.PDOT:PSS/AgNWs double network hydrogels	PEDOT:PSS+ AgNWs	196.53 Ω @ 2 MHz	High-quality ECG signals achieved with stable, clear detection of P, Q, R, S, T, and U waves;	Lower impedance (196.53 Ω vs. 213.23 Ω, Ag/AgCl);	1. Tensile strength: 147.6 kPa; 2. Maximum strain: 307.2%; 3. Adhesion: 12.54 ± 0.43 kPa; 4. Continuous monitoring: 8 h without signal loss; 5. ≥97.9% differentiation between three body states	ECG	[270]
PEDOT:PSS/PVA/borax hydrogels	PEDOT:PSS	~45% reduction compared to commercial wet electrodes	15–22 dB	Superior to commercial wet electrodes	1. Fully reusable electrode material; 2. Autonomous repair of mechanical damage; 3. Multi-Modal Signal Acquisition	ECG/EMG/EEG	[274]
SA/PVA/PAM/SGP dual-network composite hydrogel	Silver-coated glass powder (SGP) + SA + NaCl	145.63 kΩ @ 10 Hz:	35.03	25.57	1. Superior Impedance Performance; 2. High SNR Across Motion States; 3.Forty-eight-hour Continuous Monitoring; 4. Excellent Biocompatibility	ECG	[55]
PVA/CS/PEDOT:PSS	PEDOT:PSS	reducing impedance for improved signal acquisition performance	45.16	39.33 (Ag/AgCl)	1. High adhesion and durability; 2. Six dB improvement over commercial electrodes	ECG/EMG/EEG	[283]
PVA/PVP/PEDOT:PSS	PEDOT:PSS	Minimal interfacial contact impedance with skin	4.9 μV^2^	−1541.8 μV^2^ (Commercial gel electrodes); ->34.4 μV^2^ (Ag/AgCl electrodes)	1. Remarkable strain insensitivity and motion artifact rejection	sEMG	[287]
PVA-TA-0.5/LiCl-1	LiCl	Not explicitly reported	significantly lower than commercial Ag/AgCl gel electrodes	Not explicitly reported	1. An excellent balance of adhesion strength, conductivity, and mechanical stability; 2. Successfully captures clear EMG signals with regular waveforms and accurately monitors	EMG	[279]
PVA/PNIPAM	I^−^, K^+^	lower skin impedance below LCST	-I^−^-treated hydrogel: 45.22 dB/39.16 dB; -K^+^-treated hydrogels: 36 dB	Not explicitly reported	1.One hundred percent gesture recognition accuracy; 2. Programmable material compliance; 3. Thermal Stability	EMG	[34]
PTDG	Dopamine-functionalized reduced graphene oxide	Minimal impedance in the high frequency region	36.47	Not explicitly reported	1. <3% swelling in DI water after 7 days immersion; 2. <9% swelling in simulated seawater; 3.9.33 kPa wet adhesion	Underwater sEMG	[286]
PVA/AA-SBMA	Poly SBMA	Not explicitly reported	Not explicitly reported	Not explicitly reported	1. Anti-swelling dual-network strategy: 80% swelling ratio after 120 h immersion in DI water	ECG/EMG	[280]
PVA/Gelatin/TA/MXene (PGTM Dual-Network)	MXene/TA	17.18 ± 0.93 kΩ @ 10 Hz	Not explicitly reported	Not explicitly reported	1. Metal-like conductivity of MXene nanosheets; 2. Anti-drying properties via glycerol incorporation; 3. MXene-TA dual-network strategy minimizes swelling	ECG/EEG/EMG	[292]
PVA/SA/PEDOT:PSS (PSPP)	PEDOT:PSS	lowest skin-contact impedance compared to traditional Ag/AgCl electrodes	ECG: 49.68 dB; EMG: 39.56 dB; EEG: 10.11 dB	1. ECG: 38.36 dB (gel electrode); 2. EMG: 39.24 dB (Ag/AgCl electrode)	1. One-pot freeze-thaw synthesis; 2. Cost-effective fabrication; 3. Multi-stimuli sensing	ECG/EEG/EMG	[293]
PVA/Bn/PEI/MXene Dual-Network Hydrogel	MXene	Not explicitly reported	Not explicitly reported	Not explicitly reported	1. Ultrafast self-healing (~0.06 s); 2. Direction-aware strain sensing; 3. Dual reversible dynamic covalent bonding; 4. Moldable and recyclable	real-time biomechanical monitoring	[297]
PVA/Borax/TOCNF/MXene (PBTM-3 Dual-Network)	MXene	~3 kΩ	Not explicitly reported	Not explicitly reported	1. Exceptional environmental stability under extreme conditions; 2.One hundred percent antibacterial rate; 3. Water retention: 94% at 38 °C & 52% RH for 6 h; 4. Strong self-adhesion	cardiorespiratory monitoring	[295]

**Table 7:** Summary of representative PVA-based hydrogels applied in pH responsive.

Hydrogel System	Conductive Component	pH Responsive Performance	Highlight	Main Application	Ref.
NMAL	PANI (Polyaniline) + LMs (Ga^3+^ compounds) + micro-nano conductive fillers	1. Strain Sensing: GF = 2.5; 2. Temperature Response: −2.9%/°C (20–40 °C); 65%/°C (0–20 °C) 3. pH Sensing: −13.68 mV/pH sensitivity	1. Multifunctional Stimulus-Responsive; 2. High Printability; 3. Antimicrobial and Biocompatible; 4. Self-Powered Capability; 5. Exceptional Mechanical Durability	1. Wearable Health Monitoring; 2. Pressure & Touch Sensing; 3. Bioelectronic Medical Devices	[310]
PVA/P(AA-HEMA-SSS)	Sodium Styrenesulfonate (SSS)	1. Strain Sensing: GF = 3.55 at 0–400% strain; GF = 7.21 at 400–800% strain;	1. Cross-Coupled Multifunctional Sensing; 2. pH-Controlled Drug Release; 3. Enhanced Stability Through Microstructure Optimization; 4. Excellent Bio-compatibility	1. Controlled Drug Delivery System; 2. Flexible Wearable Strain Sensors; 3. Health and Motion Monitoring Devices	[279]
PPMP@S/ApoV system	PEDOT:PSS + PPy + MXene	1. Dynamic pH responsiveness across 4.5–6.5	1. ROS-Scavenging Nanozyme; 2. Self-Healing and pH-Responsive; 3. Enhanced Antibacterial Properties; 4. Bioelectrical Signal Conductivity;	1. Smart Bioelectrode Interface; 2. Flexible Bioelectronics	[50]
PVA/SA with Triple Physical Cross-linking	SA + Ca^2+^ ions	1. Strain Sensing Range: 0–580% with varying GF = 2.41–10.29); 2. Dynamic swelling responses across acidic/neutral/alkaline conditions	1. Triple Physical Cross-Linking Architecture; 2. Exceptional Mechanical Properties; 3. Wide Strain Sensitivity; 4. pH-Responsive Swelling	1. Non-invasive wearable devices;	[316]
PNIPAM/GO/PVA/Na^+^	GO + Na^+^	1. Temperature Sensitivity: −2.23%/°C @ 29.2–43.9 °C; Resolution: 0.1 °C; Response time: 321 ms; 2. pH Sensitivity: 53.08%/pH across pH 1.00–9.18; Response time: 481 ms	1. GO + Na^+^ synergy enhances overall sensing capability; 2. Combines temperature and pH sensing in single device; 3. Biocompatible;	1. Wearable Health Monitoring; 2. Sweat pH Monitoring; 3. Dual-Modality Biosensors; 4. Medical Diagnostics	[317]
CDs/CL/PVA	CDs + Carbonized Lignin (CL)	1. Strain Sensing: GF = 1.64; 2. Multi-Stimulus Responsiveness: Strain, temperature, and pH changes	1.One hundred percent Biodegradable and Green Chemistry; 2. Synergistic Conductive; 3. Exceptional Mechanical Robustness; 4. Hot-Melt Recyclability	1. Wearable Motion Sensors; 2. pH-responsive sweat detection;	[318]

**Table 8:** Summary of representative PVA-based photoresponsive conductive hydrogels.

System	Conductive Component	Sensing Range	Highlight	Application	Ref.
PNIPAM/AM/PVA	MXene + P(VDF-TrFE)/BTO piezoelectric thin film	Strain: GF = 1.78 (0–5%); Bending: GF = 1.74 (0–100°)	1. Multimodal sensing; 2. Photothermal NIR actuation; 3. Response time: 235 ms	1. Soft robotics, artificial muscles, intelligent actuators	[323]
PVA/CaP/PGO	PGO; Ca^2+^/PO_4_^3−^	Not specified	1. Dual therapeutic function; 2. Antibacterial and biocompatible	1. Smart wound dressing; 2. ECG/EMG	[324]
PVA/MXene	MXene	Strain: GF = 1.78	1. Dual-state shape-memory; 2. Photothermal NIR responsiveness; 3. Real-time feedback via conductivity change	1. Adaptive electronics, collapsible/reconfigurable devices; 2. Programmable robotics	[49]
PVA-GEL-GL-Mo_7_ Dual-Stimuli-Responsive Ion-Conductive Hydrogel	Benzimidazole methyl trimethylammonium hydroxide (BzMe_3_NOH) ionic solvent;	-Strain sensing: Relative resistance change 36% @ 30% strain, 51% at 50% strain, 73% at 100% strain; -Temperature sensing: Negligible thermal hysteresis	1. Dual-stimuli responsiveness: UV light and mechanical stress; 2. Excellent mechanical properties: Tensile strength 2.2 MPa, elongation 522%, toughness 5.26 MJ/m^3^; 3. Anti-freezing capability:	1. Optical display devices; 2. Bionic skins and interactive wearable devices; 3. Dual-responsive smart systems for environmental UV monitoring	[326]
PVA/azobenzene crosslinkers	Fn-SWCNT	-Photo-responsive conductance switching: Photocurrent increases from 10 µA (visible light) to 60 µA (UV 365 nm)	1. Dynamic photo-responsive conductance switching;	1. Soft robotics and contactless actuators; 2. Flexible electronics with optical control	[327]

**Table 9:** Summary of representative PVA-based hydrogels for selected applications in biochemical sensing.

Hydrogel/Platform	Conductive Component	Target Analyte	Sensing Properties	Main Application	Ref.
PANI/PVA	PANI	Glucose	-Sensitivity: 58.91 μA·mM^−1^·cm^−2^; -LOD: 0.001 μM	1. Enzymatic glucose biosensors	[335]
Ni/PEDOT:PSS/PE	PEDOT:PSS + electrochemically deposited Ni nanoparticles	Glucose in human sweat	-Detection Range: 2.0 μM–2.8 mM; -Sensitivity: 262.17 μA·mM^−1^·cm^−2^ (at 0.03–0.40 mM) & 141.29 μA·mM^−1^·cm^−2^ (0.40–0.70 mM); -LOD: 0.37 μM	1. Non-invasive wearable glucose sensors; 2. Sweat-based biomarker detection	[336]
CMC/PVA/PDA/GLY Janus Hydrogel	GOD@PEI@AuNRs	Glucose in human sweat	-Detection Range: 10–350 μM; -LOD: 0.21 μM	1. Non-invasive wearable sweat glucose sensors	[337]
CNF-Networked MXene/PEDOT:PSS-Impregnated PVA/PAAM Organohydrogel	MXene + PEDOT:PSS + CNF+ Borax	UA in human sweat	-Detection Range: 20 μM–14 mM; -LOD: 10.93 μM	1. Multifunctional Wearable Sensor; 2. Non-invasive sweat biomarker tracking	[341]
MXene-PEDOT-PVA Ternary Hydrogel	MXene + PEDOT	UA in Human Sweat	-Linear Range: 1–400 μM; -Sensitivity: 0.289 μA·μM^−1^·cm^−2^; -Detection Limit (LOD): 0.57 μM; -Response Time: ~5 s	1. Non-invasive Wearable UA Detection	[4]
PVA–PEGDA–SA Porous Hydrogel	BTXI-DPA	Cortisol	-Detection range: 0.1–10 µg/dL; -Current response: 0.6 µA	1. Wearable biosensing devices 2. Cortisol stress monitoring	[345]

## Data Availability

No new data were created or analyzed in this study. Data sharing is not applicable to this article.

## Abbreviations

Polyvinyl alcohol (PVA); sodium alginate (SA); zinc ions (Zn^2+^); freeze/thaw (F/T); dimethyl sulfoxide (DMSO); electron beam irradiation (EBI); chitosan (CS); polyvinylpyrrolidone (PVP); carbon–carbon (C-C) bonds; glutaraldehyde (GA); ammonium persulfate (APS); polyacrylamide (PAAm, PAM); hydroxypropyl chitosan (HPCS); graphene oxide (GO); poly(2-methoxyethyl acrylate) (PMEA); dopamine (DA); extracellular matrix (ECM); carbon nanotubes (CNT); reactive oxygen species (ROS); 1-ethyl-3-methylimidazolium tetrafluoroborate (EMIMBF4); sulfomethylated lignin (SML); quaternized chitosan (QCS); carbon dots (CDs); silk fibroin (SF); gauge factor (GF); glycerin (Gly); lithium chloride (LiCl); silk fibroin/polyvinyl alcohol/glycerin/lithium chloride hydrogel (SPGL); poly(3,4-ethylenedioxythiophene):poly(styrenesulfonate) (PEDOT:PSS); poly(N-isopropylacrylamide-*co*-hydroxymethylacrylamide) (P(NIPAM-*co*-NMA)); liquid metal (LM); triboelectric nanogenerator (TENG); poly(sodium 4-styrenesulfonate) (PSS); cellulose nanofibrils (CNFs); carboxymethyl cellulose (CMC); glucose adaptive hydrogel (GAH); glucose oxidase (GOx); functionalized multiwalled carbon nanotubes (f-MWCNTs); polyvinyl alcohol/poly(2-methoxyethylacrylate) (PVA/PMEA); U.S. Food and Drug Administration (FDA); reduced graphene oxide (rGO); bilirubin/morin nanoparticles (BMn); poly(N-isopropylacrylamide) (poly(NIPAM), PNIPAM); hydroxymethyl acrylamide (NMA); microfibrillated cellulose (MFC); ionic liquid (IL); konjac glucomannan (KGM), iron-based metal–organic framework (Fe-MOF); polypyrrole (PPy).
